# BRD4-mediated transcriptional activation of PDLIM4 enhances p21 stability and chemosensitivity in lung adenocarcinoma independent of p53

**DOI:** 10.1186/s12915-026-02511-z

**Published:** 2026-01-17

**Authors:** Qingwei Wang, Liangsheng Guo, Shuai Wang, Chengdan Guan, Junhao Pan, Shaoping Zhu, Lei Zheng, Xuehua Wu, Yonghui Gu, Tao Shu, Lianxiang Luo, Tianwen Lai, Xiao Gao

**Affiliations:** 1 Zhanjiang Key Laboratory of Hepatobiliary-Related Diseases, The Affiliated Hospital of Guangdong Medical University, Zhanjiang, 524023 China; 2 Institute of Respiratory Diseases, The Affiliated Hospital of Guangdong Medical University, Zhanjiang, 524023 China; 3 Institute of Laboratory Animal Center, Guangdong Medical University, Zhanjiang, 524023 China; 4 The Marine Biomedical Research Institute of Guangdong Zhanjiang, School of Ocean and Tropical Medicine, Guangdong Medical University, Guangdong, 524023 China; 5 Department of Respiratory and Critical Care Medicine, Key Laboratory of Immune Inflammation and Metabolism, The First Dongguan Affiliated Hospital, Guangdong Medical University, Dongguan, 523710 China; 6 Zhanjiang Key Laboratory of Tumor Microenvironment and Organoid Research, Zhanjiang, 524023 China

**Keywords:** PDLIM4, BRD4, P21, P53 independent, Lung adenocarcinoma

## Abstract

**Background:**

Understanding p53-independent regulatory mechanisms is crucial for predicting outcomes in lung adenocarcinoma (LUAD) and developing improved therapeutic strategies.

**Results:**

We found that PDLIM4 is highly expressed in LUAD tumor tissues, where it induces G2/M phase cell cycle arrest and suppresses cell proliferation, suggesting its potential role in improving patient prognosis. Our study identified BRD4, a bromodomain and extraterminal (BET) family protein, as a key transcriptional regulator of PDLIM4, acting through its BD1 domain. Further analysis revealed that wild-type PDLIM4 stabilizes p21 by blocking its RNA degradation, leading to p21 protein accumulation and subsequent inhibition of cell proliferation. In contrast, the S116 mutation in PDLIM4 abrogates this regulatory effect. Notably, activation of the BRD4/PDLIM4/p21 pathway enhanced chemosensitivity to doxorubicin in both LUAD cells and xenograft tumor models.

**Conclusions:**

Given the high mutation frequency of PDLIM4 recorded in the TCGA cancer database, our findings reveal a critical regulatory signaling pathway that suppresses LUAD progression and augments chemotherapy efficacy.

**Supplementary Information:**

The online version contains supplementary material available at 10.1186/s12915-026-02511-z.

## Background

Lung adenocarcinoma (LUAD) is one of the most prevalent and aggressive types of lung cancer, characterized by its high malignancy, rapid progression, and frequent distant metastases at the time of diagnosis [1]. Despite advances in understanding its molecular mechanisms, the pathogenesis of LUAD remains incompletely understood, and its prognosis is still poor. Therefore, identifying new therapeutic targets is crucial for improving diagnosis and prognostic prediction in LUAD.

The tumor suppressor gene p53 plays a pivotal role in regulating lung adenocarcinoma development [2]. Wild-type p53 activates p21, which subsequently inhibits the cell cycle, induces DNA repair, and promotes apoptosis in response to DNA damage, thus suppressing tumor growth [3]. P21 exerts its effect by inhibiting cyclin-dependent kinases (CDKs), thereby blocking cell cycle progression. This makes p21 a critical component of the p53-dependent growth control pathway in mammalian cells [4–6]. However, mutations and deletions of p53 are among the most common genetic alterations in LUAD, with mutation rates reaching as high as 50%–80% [7–11]. Mutant p53 causes dysregulation of p21 and disrupts cell cycle checkpoints, allowing cells harboring these mutations to evade normal growth control and proliferate uncontrollably, thus promoting tumor progression [12, 13]. Therefore, exploring p53-independent pathways that regulate p21 is critical for developing new therapeutic strategies for LUAD [14, 15].

PDLIM4 (PDZ and LIM domain 4) is a member of the PDLIM family of proteins, which contain both PDZ and LIM structural domains [16–18]. These proteins are involved in anchoring signaling molecules to the actin cytoskeleton through their PDZ domains and in interacting with kinases via their LIM domains [19–21]. It has been reported that PDLIM4 has the ability to regulate tumor progression. In colon cancer, PDLIM4 overexpression inhibits cell growth and clone formation [22]; in prostate cancer, PDLIM4 is lowly expressed and inhibits the cell cycle [23]; and in ovarian cancer, low PDLIM4 expression suggests a poor prognosis, and PDLIM4 regulates tumor progression by inhibiting STAT3 [24]. However, the specific role of PDLIM4 in lung adenocarcinoma and its mechanism remain unclear.

Members of the bromodomain and extraterminal (BET) protein family, including BRD4, are associated with acetylated chromatin and play key roles in gene transcription regulation by binding to the transcription start sites of mitosis-associated genes [25–27]. JQ1, a small molecule inhibitor of BET proteins, suppresses the expression of downstream genes by competitively binding to BET proteins and preventing their attachment to acetylated lysines on chromatin [28]. BRD4, a critical member of the BET family, regulates gene expression by interacting with chromatin and recruiting transcriptional machinery to target promoters [29, 30]. Several oncogenes, including MYC, BCL2, WNT5A, KIT, FOSL, p53 and RUNX2, have been identified as BRD4 substrates, underscoring its importance in tumorigenesis [30–36].

In this study, we investigated the expression of PDLIM4 in LUAD and its potential prognostic significance. Our findings show that PDLIM4 is highly expressed in LUAD tumor tissues. Both in vitro and in vivo experiments confirmed that PDLIM4 overexpression inhibits cell proliferation, induces cell cycle arrest, and suppresses xenograft tumor growth in mice, suggesting that PDLIM4 expression may be associated with a favorable prognosis. We further demonstrated that BRD4 directly regulates the transcription of PDLIM4, which, through a p53-independent mechanism, prevents the degradation of p21 RNA, leading to p21 accumulation. Interestingly, mutant PDLIM4 (S116) loses this regulatory function. Moreover, activation of the BRD4/PDLIM4/p21 axis enhances chemosensitivity to doxorubicin in LUAD. These results suggest that targeting the BRD4/PDLIM4/p21 pathway could provide a promising therapeutic strategy for LUAD, with potential implications for prognosis prediction and chemotherapy response.

## Results

### Upregulation of PDLIM4 expression is correlated with a good prognosis in LUAD

**Fig. 1:**
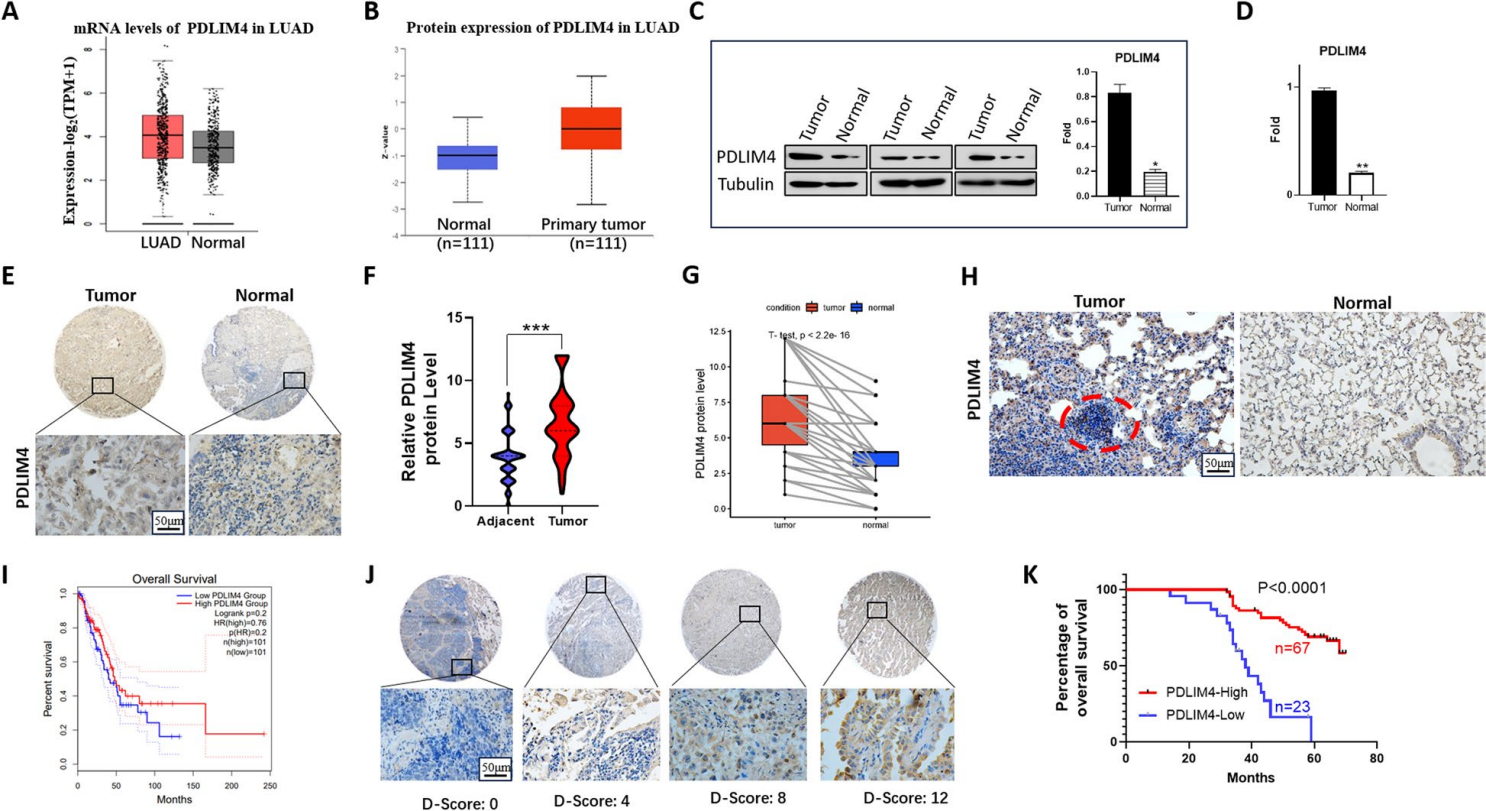
PDLIM4 is highly expressed in LUAD and may be associated with a good prognosis. **A** The mRNA expression levels of PDLIM4 in normal and LUAD tissues were evaluated via the GEPIA webtool. **B** The protein levels of PDLIM4 in LUAD and normal tissues from CPTAC were analyzed by UALCAN portal. **C** Western blotting showed PDLIM4 expression in fresh clinical lung adenocarcinoma tissue and adjacent tissue (*n* = 10, left). The relative protein levels were quantified by gray value and normalized to tubulin (right). Data are represented as the mean ± SEM. *p < 0.05. **D** Real-time PCR showed PDLIM4 expression in human LUAD tissues (*n* = 10) and adjacent tissues. Data are represented as the mean ± SEM. **p < 0.01. **E** Immunohistochemistry (IHC) staining of PDLIM4 expression in LUAD tissues and adjacent normal tissues. Scale bars, 50 μm. **F** Statistical analysis was performed using the paired two-tailed Student’s *t*-test. ***p < 0.001. **G** The relative protein level of PDLIM4 in LUAD tissues or paired adjacent tissues was determined by IHC. **H** IHC staining for PDLIM4 was performed in mouse lung cancer tissues and normal lung tissues. **I** Analysis of relapse-free survival of patients in the TCGA dataset. **J** Representative results of IHC staining of PDLIM4 on LUAD tissues and paired adjacent tissues (*n* = 90). Based on staining scores of PDLIM4 (D-scores) suggested by the pathologist, patients were separated into two groups: high PDLIM4 (D-scores > = 6) and low PDLIM4 (D-scores < 6). Metastatic events are characterized by regional or distant metastasis. **K** Kaplan–Meier analysis showing the relationship between overall survival of LUAD patients with PDLIM4 expression

To investigate the implications of PDLIM4 in LUAD, we analyzed the available public dataset TCGA and found that, compared with normal lung tissues, PDLIM4 mRNA expression was significantly increased in tumor tissues (Fig. 1A). Then, we explored the expression of PDLIM4 protein in LUAD using the Clinical Tumor Proteomics Analysis Consortium (CPTAC) database and found that its protein level was also elevated in tumors (Fig. 1B). Subsequently, fresh lung cancer tissues and adjacent normal tissues were collected to detect the expression of PDLIM4 by RT-PCR and Western blotting. The results showed that the expression of PDLIM4 protein and mRNA in LUAD tissues was significantly higher than in adjacent tissues (*n* = 10, Fig. 1C, D). Ninety patients with adenocarcinoma and the corresponding adjacent tissues were analyzed by immunohistochemical staining with PDLIM4 antibody; clinicopathological correlation analysis revealed that the expression of PDLIM4 was obviously increased compared with adjacent tissues in LUAD, further verifying our above results (Fig. 1E, F, G). To analyze the expression pattern of PDLIM4 in mouse lung tumors, we induced a mouse model of lung cancer with urethane, and immunohistochemical assays showed elevated expression of PDLIM4 in the tumors compared with normal mouse lung tissues (Fig. 1H). We further explored the correlation between patient survival and PDLIM4 expression using the publicly available TCGA database and 90 pairs of clinical lung adenocarcinoma and paraneoplastic samples, respectively. Kaplan–Meier analysis revealed a significant correlation between elevated PDLIM4 levels and prolonged overall survival as well as disease-free survival (Fig. 1I, J, K). These data demonstrated that PDLIM4 is a potential indicator of a good prognosis for LUAD.

### PDLIM4 acts as a tumor suppressor in LUAD

**Fig. 2:**
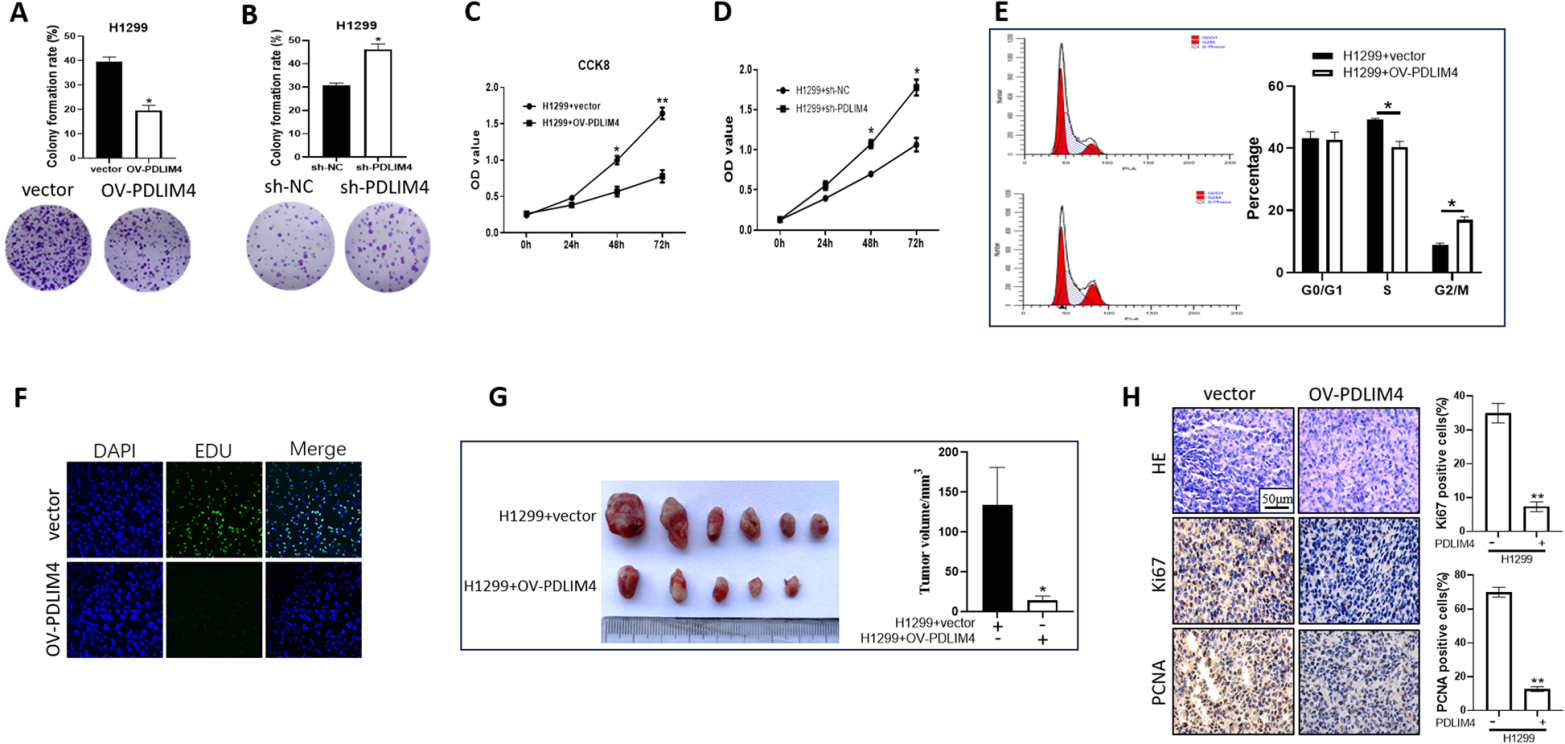
PDLIM4 inhibits cell growth and arrests cell cycle in LUAD. **A**, **B** The representative images (lower) and colony numbers quantitation (> 1 mm, upper) were performed in H1299 stable overexpression of PDLIM4 (OV-PDLIM4) or PDLIM4 depletion (sh-PDLIM4) cell lines. Data are represented as the mean ± SEM. *p < 0.05. **C**, **D** CCK-8 assays were displayed after overexpression of PDLIM4 or PDLIM4 depletion in H1299 cells. Data are represented as the mean ± SEM. **p < 0.01, *p < 0.05. **E** Stable overexpression of PDLIM4 in H1299 cell lines was stained with propidium iodide (PI) and the flow cytometry assayed for cell cycle. *p < 0.05. **F** The representative images of EdU incorporation in vector and OV-PDLIM4 cells. **G** In vivo tumorigenicity in nude mice evaluated by subcutaneous injection of vector and OV-flag-PDLIM4 H1299 cells, showing representative images of xenograft (left) and the statistical results of tumor volume (right). Data are represented as mean ± SEM (analysis of variance, *n* = 6, *p < 0.05). **H** Representative images of HE and IHC (left) and statistical results (right) were presented by Ki67 or PCNA staining of **G**. All data in this figure are representatives of three independent repeats

PDLIM4 has been reported to suppress the growth of colon cancer, prostate cancer, and ovarian cancer [23, 24, 29]. To explore the potential function of PDLIM4 in the development of lung adenocarcinoma (LUAD), we utilized a recombinant plasmid containing the PLVX-EF1a-IRES-Puro-flag-PDLIM4 sequence to successfully establish PDLIM4-overexpressing models in H1299 and A549 cells while employing pLKO.1-shPDLIM4 to achieve knockdown of endogenous PDLIM4 in 293 T, H1299, and A549 cell lines. Western blot analysis demonstrated that PDLIM4 protein expression was significantly increased in the overexpression cell lines (Supplementary Fig. 1A, B), whereas it was notably reduced in the knockdown groups (Supplementary Fig. 1C, D, E). Compared with the control cells, the H1299 and A549 cells with PDLIM4 overexpression exhibited significantly decreased cell clone formation (Fig. 2A, Supplemental Fig. 2A) and cell proliferation (Fig. 2C, Supplemental Fig. 2C); however, the cells with PDLIM4 knockdown significantly promote cell clone formation (Fig. 2B, Supplemental Fig. 2B) and proliferation (Fig. 2D, Supplemental Fig. 2D). Additionally, flow cytometric assays showed that overexpression of PDLIM4 markedly increased the percentage of G2/M phase cells while decreasing the percentage of S phase cells in H1299 and A549 cells (Fig. 2E, Supplemental Fig. 2E). Besides, Edu incorporation assay was performed as illustrated in Fig. 2F. Upregulation of PDLIM4 dramatically decreased the percentage of H1299 cells that incorporated Edu.

To further explore the possible role of PDLIM4 in tumorigenesis in vivo, xenograft experiments were performed by injecting control (vector) or cells stably overexpressing PDLIM4 (OV-PDLIM4) subcutaneously into the right anterior armpit of BALB/c nude mice. Images and quantitative analyses showed that the cells formed smaller tumor volumes compared to the control group (Fig. 2G, Supplemental Fig. 2F). Moreover, immunohistochemical staining demonstrated that OV-PDLIM4 xenografts had a marked decrease of Ki67 and PCNA-positive nuclei than that in the control group (Fig. 2H, Supplemental Fig. 2G). In summary, these results suggested an inhibitory effect of PDLIM4 on LUAD cells by inducing cell cycle arrest.

### BRD4 regulates the transcription of PDLIM4 in LUAD cells

**Fig. 3:**
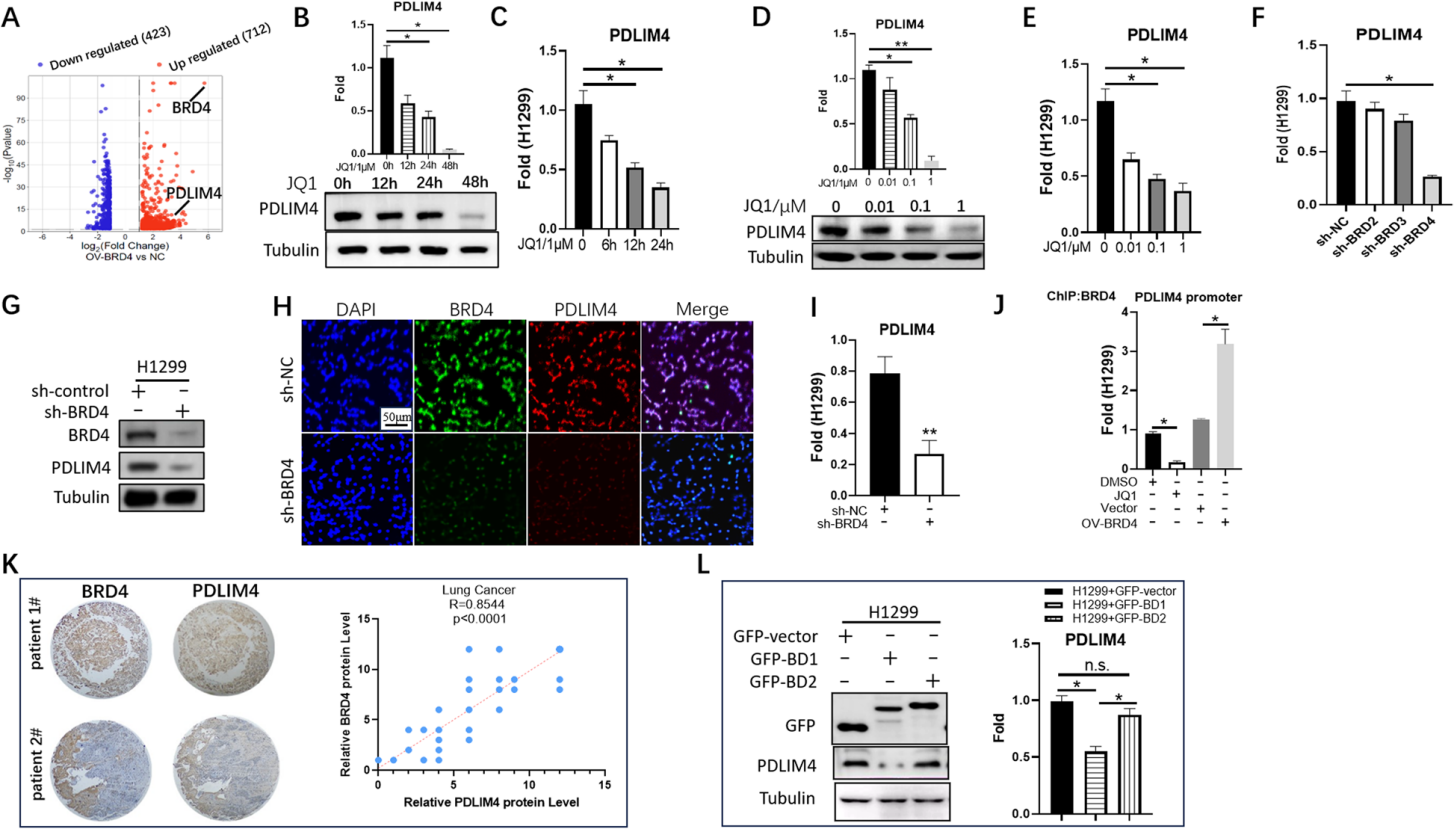
Identification of BRD4 acts as a direct upstream of PDLIM4. **A** The volcano plot of the differently expressed genes in RNA-seq. **B** Western blot analysis of the expression of PDLIM4 in H1299 cells treated with 1 μM JQ1 for 12 to 48 h. Data are represented as the mean ± SEM. *p < 0.05. **C** Real-time PCR analysis of the expression of PDLIM4 in H1299 cells treated with 1 μM JQ1 for 6 to 24 h. Data are represented as the mean ± SEM. *p < 0.05. **D** Western blot analysis of the expression of PDLIM4 in H1299 cells treated with the indicated concentration of JQ1 for 48 h. Data are represented as the mean ± SEM **p < 0.01, *p < 0.05. **E** Real-time PCR analysis of the expression of PDLIM4 in H1299 cells treated with the indicated concentration of JQ1 for 48 h. Data are represented as the mean ± SEM. *p < 0.05. **F** The expression of PLDIM4 was detected by real-time PCR after stable knockdown of BRD2 or BRD3 or BRD4 in H1299 cells. Data are represented as the mean ± SEM. *p < 0.05. **G** Western blot analysis of PDLIM4 in BRD4 knockdown cells. **H** Immunofluorescence detection of PDLIM4 (red) and BRD4 (green) expression following BRD4 deletion. Scale bar: 50 μm. **I** Real-time PCR analysis of the expression PDLIM4 in BRD4 stable knockdown cells. **J** H1299 cells were treated with either JQ1 for 48 h or overexpression BRD4 as indicated and harvested for ChIP assay subsequently. *p < 0.05. **K** Representative plots of immunohistochemistry (IHC) staining for BRD4 and PDLIM4 in clinical LUAD samples (left), correlation between BRD4 and PDLIM4 protein expression in LUAD (right), displayed by scatter plot. *n* = 90, *p* < 0.0001. Scale bar, 200 μm (magnification, × 10). **L** Western blot analysis of the expression of PDLIM4 after transient transfection of BRD4-BD1 or BRD4-BD2 in H1299 cells. Data are represented as the mean ± SEM. *p < 0.05. Data are represented as the mean ± SEM of three independent experiments

Our study indicated that high expression of PDLIM4 can significantly inhibit tumor proliferation; therefore, exploring upstream regulators would be crucial for tumor therapy. Our team previously performed RNA-seq analysis to screen for potential target genes of BRD4 in the hepatocellular carcinoma cell line PLC-5 (the original data is in Supplemental Table 3). Compared with the control groups, 260 genes were upregulated and 182 genes were downregulated after overexpression of BRD4 (OV-BRD4), among which PDLIM4 presented heightened regulation (Supplemental Fig. 2H); to further validate this, we performed RNA-seq analysis using control and BRD4-overexpressing H1299 cells. Among the 712 genes showing upregulated expression trends upon BRD4 overexpression, PDLIM4 was identified as one such candidate (Fig. 3A) (the original data is in Supplemental Table 4). The BET bromodomain inhibitors (BETi) have been evaluated and shown efficacy in several models of cancer, and JQ1 has been proven to be a first-in-class BETi, which can downregulate the transcription of multiple genes by targeting BRD4, such as MYC, IL7R, and E2F1 in different cancer cells [27]. Herein, we found that the protein levels (Fig. 3B, D, Supplemental Fig. 3A, C) and mRNA levels (Fig. 3C, E, Supplemental Fig. 3B, D) of PDLIM4 decreased dramatically in a dose-dependent and time-dependent manner in H1299 and A549 cells after JQ1 treatment, suggesting that the expression of PLDIM4 is inhibited at the transcriptional level by BETi.

To explore the pathway and BET proteins more deeply, we undertook a specific siRNA-knockdown strategy to investigate the requirement for each BET protein separately, due to JQ1 was a pan inhibitor for all BET family members [37]. AZD5153 is a potent selective and orally available BET/BRD4 bromodomain inhibitor that can simultaneously bind to both bromodomains of BRD4 [38]. Treatment with AZD5153 in time and concentration gradients revealed that the protein and RNA levels of PDLIM4 could both be correspondingly reduced (Supplemental Fig. 3E, F). These results suggest that the expression of PDLIM4 may be regulated by BRD4. To further validate, we measured mRNA expression of each BET gene to validate knockdown specificity and efficiency for each siRNA. We confirmed that each siRNA only ablates its targeted sequence and does not reduce mRNA of other BET family members (Supplemental Fig. 3G, H, I, J, K, L) in H1299 and A549 cell lines. We corroborated that only BRD4 knockdown downregulated both PDLIM4 mRNA and protein (Fig. 3F, G, H, I, Supplemental Fig. 3M, N, O, P, Q, R, S). Notably, knocking down BRD2 or BRD3 alone does not have a significant regulatory effect on PDLIM4. Only when BRD4 is knocked down simultaneously can induce a significant reduction in the protein and RNA levels of PDLIM4 (Supplemental Fig. 3M, N, O, P and Supplemental Fig. 3T, U, V, W). Immunofluorescence detection revealed that BRD4 was colocalized with PDLIM4 (Fig. 3H), and chromatin immunoprecipitation (ChIP) assays showed recruitment of BRD4 to the PDLIM4 promoter following JQ1 treatment or overexpression of PDLIM4 (Fig. 3J, Supplemental Fig. 3X), demonstrating that PDLIM4 is a BRD4-specific target gene. Furthermore, 90 patients with adenocarcinoma and the corresponding adjacent tissues were analyzed by immunohistochemical staining with PDLIM4 or BRD4 antibody, and clinicopathological correlation analysis revealed that there was a positive correlation between the expression level of PDLIM4 and the expression level of BRD4 in LUAD (Fig. 3K). These results provided a mechanistic explanation for the control of PDLIM4 expression by BRD4.

BRD4 has two tandem N-terminal bromodomain (BD), specifically BD1 and BD2. Differential BD affinity among BET inhibitors may determine their functional effects [39]. PDLIM4 was detected in cells transiently transfected with BRD4-BD1 or BRD4-BD2, respectively, and it was found that overexpression of BD1 was able to inhibit the promotional effect of BRD4 on the expression of PDLIM4 (Fig. 3L, Supplemental Fig. 3Y), indicating that BRD4 exerted its regulation of PDLIM4 through the BD1 domain.

### Identification of p21 as a downstream regulatory substrate for PDLIM4

**Fig. 4:**
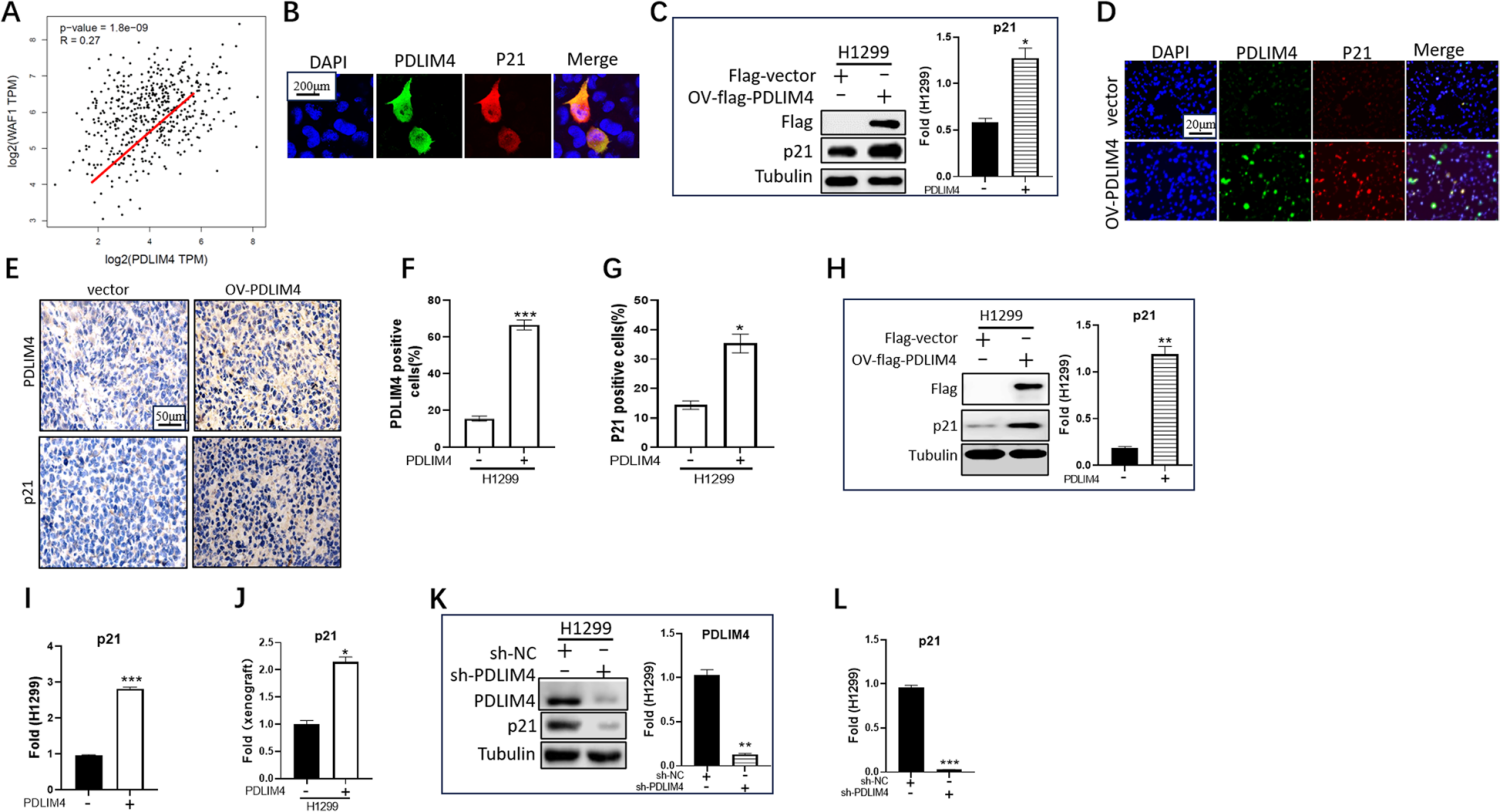
Cell cycle is regulated by PDLIM4 through p21. **A** Using the GEPIA2 approach (http://gepia2.cancer-pku.cn/), the expression correlation between PDLIM4 and p21 (WAF1) was analyzed in TCGA LUAD tumor projects (*R* = 0.27, *p*-value = 1.8e-09). **B** Immunofluorescence-stained endogenous PDLIM4 (green) and p21 (red) in H1299 cells were visualized by confocal microscopy. DAPI staining was included to visualize the cell nucleus (blue). Scale bar: 200 μm (magnification, × 40). DAPI, 4′,6‐diamidino‐2 phenylindole. **C** The relative expression levels of p21 and flag-PDLIM4 were analyzed by Western blotting following stable overexpression of PDLIM4. Data are represented as mean ± SEM. *p < 0.05. **D** Immunofluorescence-stained endogenous PDLIM4 (green) and p21 (red) in vector or OV-PDLIM4 of H1299 cells were visualized by confocal microscopy. Scale bars: 50 µm. **E**, **F**, **G** Representative plots of immunohistochemistry (IHC) staining for PDLIM4 and p21 in mice xenograft tumor derived from vector or OV-PDLIM4 of H1299 cells. **H** The relative expression levels of p21 and flag-PFLIM4 were analyzed by Western blotting in mice xenograft tumor derived from vector or OV-PDLIM4 of H1299 cells. Data are represented as mean ± SEM. *p < 0.05. **I** Real-time PCR assay analysis of the expression of p21 expression in vector or OV-PDLIM4 of H1299 cells. Data are represented as mean ± SEM. ***p < 0.001. **J** Real-time PCR assay analysis of the expression of p21 expression in mice xenograft tumor. Data are represented as mean ± SEM. *p < 0.05. **K** The relative expression levels of p21 and PDLIM4 (left) were analyzed by Western blotting after stable knockdown of PDLIM4 in H1299 cells. Quantitative data (right) are represented as mean ± SEM. **p < 0.01. **L** Real-time PCR assay analysis of the expression of p21 after stable knockdown of PDLIM4 in H1299 cells. Data are represented as mean ± SEM. ***p < 0.001. All experiments were repeated at least three times

Since our preliminary results showed that PDLIM4 was able to regulate LUAD proliferation by inducing cell cycle arrest (Fig. 2E), we tried to find the downstream of PDLIM4 among the cell cycle-related proteins. We obtained the available genes in TCGA LUAD tumor projects and analyzed the expression correlation between PDLIM4 and selected targeting genes, including p21 and others (not shown). This scatter plots showed that p21 expression was positively correlated with PDLIM4 expression (Fig. 4A). Immunofluorescence staining revealed that PDLIM4 co-localized with p21 in the nuclei and cytoplasm and any stage during cell cycle (Fig. 4B, Supplemental Fig. 4A). We used Western blot and immunofluorescence experiments to examine the protein expression level of p21 in cells stably overexpression of PDLIM4 (OV-PDLIM4) as well as in control cells (vector) and found that the p21 protein level had an obvious increase together with high PDLIM4 expression (Fig. 4C, D, Supplemental Fig. 5A). Using immunohistochemistry and Western blot to detect xenograft tumors from the stable-transfected cells in nude mice, it was found that the high expression of PDLIM4 was accompanied by a corresponding increase in the level of p21 protein (Fig. 4E, F, G, H, Supplemental Fig. 5B, C, D, E). Then, real-time quantitative PCR experiments showed that the p21 mRNA level was also increased after the elevation of PDLIM4 (Fig. 4I, J, Supplemental Fig. 5F, G). In contrast, in the case of knockdown of PDLIM4, p21 protein and mRNA levels decreased accordingly (Fig. 4K, L, Supplemental Fig. 5H, I). Therefore, we conclude that the expression of p21 was regulated by PDLIM4, which could possibly account for the ability of PDLIM4 to regulate the cell cycle and thus affect the progression of LUAD.

**Fig. 5:**
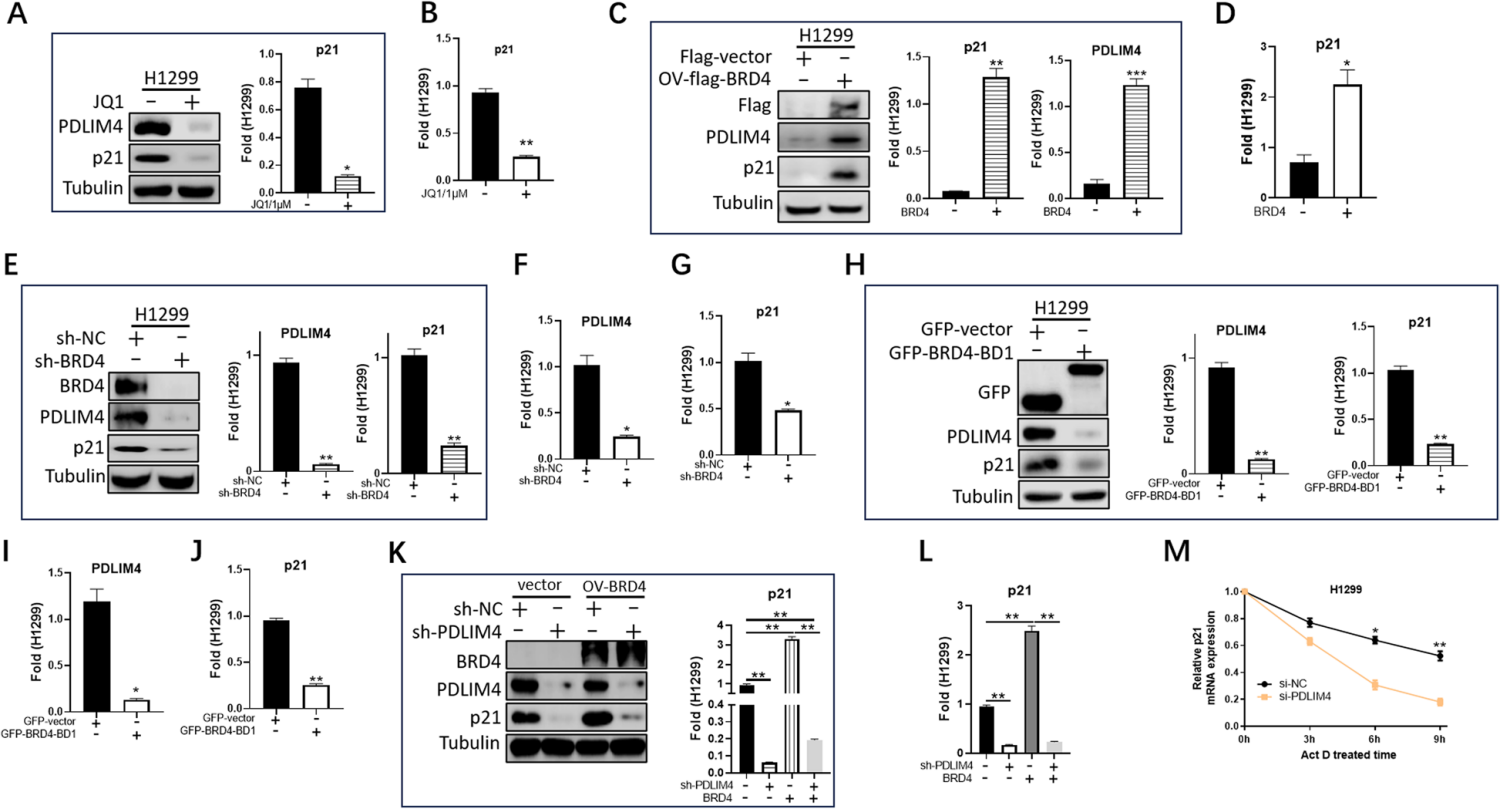
BRD4 promotes p21 signaling via transcriptional regulation of PDLIM4. **A** Western blot analysis of the PDLIM4 and p21 expression (left) in H1299 cells treated with JQ1 (1 μM) for 24 h. Quantitative data (right) represented mean ± SEM. *p < 0.05. **B** The p21 mRNA level was checked by real-time PCR in H1299 cells with JQ1 (1 μM) treatment for 6 h. Data represented mean ± SEM. **p < 0.01. **C** Immunoblotting demonstrated the expression of PDLIM4 and p21 after stable overexpression of BRD4. Data represented mean ± SEM. ***p < 0.001, **p < 0.01. **D** The mRNA level of PLDLIM4 and p21 was checked by real-time PCR after stable overexpression of BRD4. Data represented mean ± SEM. *p < 0.05. **E**, **F**, **G** The expression of PDLIM4 and p21 was detected by real-time PCR and immunoblotting after stable knockdown of BRD4 (sh-BRD4) in H1299 cells. Data represented mean ± SEM. **p < 0.01, *p < 0.05. **H**, **I**, **J** Testing the expression of PDLIM4 and p21 in control (sh-vector) and overexpression of BD1 H1299 cells by real-time PCR and immunoblotting. Data represented mean ± SEM. **p < 0.01. **K**, **L** Knockdown PDLIM4 in control and stable overexpression of BRD4 cells and testing the expression of p21 by real-time PCR and immunoblotting. Data represented mean ± SEM. **p < 0.01. **M** To maintain control or PDLIM4 knockdown cells in media containing actinomycin D (Act D, 10 μg/mL, Selleck), we utilized quantitative PCR to examine intracellular mRNA level of p21 at 0, 3, 6, and 9 h. Data represented mean ± SEM. **p < 0.01, *p < 0.05. All the experiments were repeated at least three times

### BRD4-PDLIM4 regulates the expression of p21 by inhibiting its RNA degradation

With the findings that BRD4 drives PDLIM4 transcription (Fig. 3) and p21 is regulated by PDLIM4 (Fig. 4), we hypothesized that the regulatory signal could be transmitted from BRD4 to p21. Statistical results of Western blot and real-time PCR experiments showed that PDLIM4 and p21 had a significant reduction in both protein and RNA levels following JQ1 treatment (Fig. 5A, B, Supplemental Fig. 5J, K). In contrast, both protein and RNA levels of PDLIM4 and p21 were significantly elevated after stable overexpression of BRD4 (Fig. 5C, D, Supplemental Fig. 5L, M). To confirm that BRD4 is an upstream regulator of PDLIM4-p21, we next depleted BRD4 expression by shRNA. Successful knockdown of BRD4 caused a significant reduction of PDLIM4 and p21 at both the protein and RNA levels (Fig. 5E, F, G, Supplemental Fig. 5N, O. P).

Our results showed that the BD1 domain of BRD4, rather than BD2, was responsible for the function of BRD4 in controlling PDLIM4 expression in LUAD (Fig. 3L, Supplemental Fig. 4C). Therefore, the BD1 domain was overexpressed in H1299 or A549 cells by transient transfection, and the expression levels of both PDLIM4 and p21 were significantly suppressed (Fig. 5H, I, J, Supplemental Fig. 5Q, R, S). In order to strengthen our hypothesis that the BRD4-PDLIM4-p21 signaling pathway exists in LUAD cells, overexpression of BRD4 in stable PDLIM4 knockdown cells was performed; the experimental data showed that both protein and mRNA levels of p21 did not respond to the high expressive regulation caused by BRD4 in PDLIM4 knockdown cells (Fig. 5K, L, Supplemental Fig. 5T, U). These results suggest that BRD4 could regulate p21 accumulation dependent on PDLIM4. As is well known, p53 is a crucial transcriptional regulator of p21. To determine whether the PDLIM4-p21 pathway is regulated by p53, we compared the upregulation of p21 in H1299 cells (which naturally lack p53) and A549 cells (expressing wild-type p53) following the overexpression of PDLIM4. We found that in H1299 cells, the protein level of p21 increased by approximately 5.36-fold, while in A549 cells it increased by about 5.05-fold. At the RNA level, p21 expression rose by 2.22-fold in H1299 cells and 1.98-fold in A549 cells (Supplemental Fig. 5V, W). These results suggest that the PDLIM4-p21 pathway is not dependent on p53 regulation.

Next, we tried to explore whether PDLIM4 regulates p21 directly at the protein or RNA level. Immunoprecipitations experiments revealed that enrichment of PDLIM4 was able to be pulled down p21 at the same time; on the contrary, in the enrichment of p21, PDLIM4 was not able to be pulled down (data not shown). This result shows that PDLIM4 is not tightly bound to p21, which determines that PDLIM4 may have a role in regulating the protein level of p21, but not a large regulatory role necessarily; moreover, the results of the cycloheximide (CHX) experiment indicated that the half-life of p21 protein level was not significantly affected by the high expression of PDLIM4 (Supplemental Fig. 5X). To determine if PDLIM4 affects the production or stability of p21 transcript variants in LUAD, we determined mRNA half-lives of p21 mRNA with actinomycin D which is widely used in mRNA stability assays to inhibit the synthesis of new mRNA. Under treatment of actinomycin D (Act D), p21 mRNA stability was weakened by PDLIM4 consumption (Fig. 5M, Supplemental Fig. 5Y). In summary, these data support that PDLIM4 controls the stability of p21 mRNA by affecting mRNA decay and suggests a novel cell signaling pathway BRD4-PDLIM4-p21 in LUAD.

### The BRD4-PDLIM4-p21 axis promotes sensitivity to chemotherapy

**Fig. 6:**
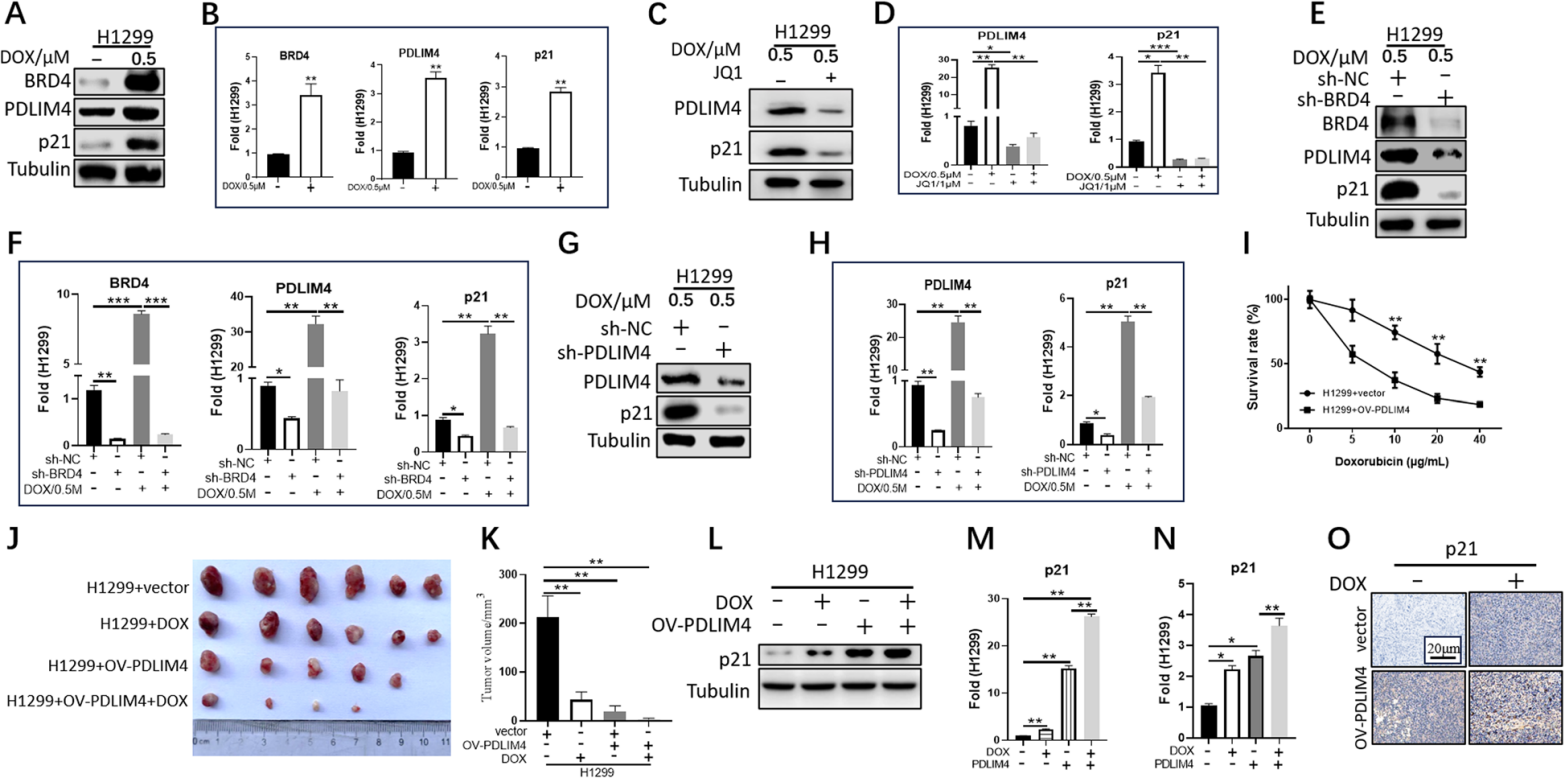
BRD4-PDLIM4 enhance chemotherapeutic sensitivity in p53-deficient cells. **A**, **B** The protein and mRNA levels of BRD4, PDLIM4, and p21 were detected in H1299 cells after 0.5-μM DOX treatment for 2 h. Data represented mean ± SEM. **p < 0.01. **C**, **D** Western blot and real-time PCR analyses of the expression of PDLIM4 and p21 in H1299 cells after JQ1 or DOX treatment. Data represented mean ± SEM. ***p < 0.001, **p < 0.01, *p < 0.05. **E**, **F** Western blot and real-time PCR analyses of the expression of BRD4, PDLIM4, and p21 in control H1299 cells or BRD4 knockdown cells following DOX treatment. Data represented mean ± SEM. ***p < 0.001, **p < 0.01, *p < 0.05. **G**, **H** Western blot and real-time PCR analyses of the expression of PDLIM4 and p21 in control H1299 cells or PDLIM4 knockdown cells following DOX treatment. Data represented mean ± SEM. **p < 0.01, *p < 0.05. **I** H1299 cells stably overexpressing PDLIM4 and control cells were treated with DOX for 48 h followed by CCK8 assays. Data represented mean ± SEM. **p < 0.01. **J** Xenograft tumors were generated by injecting H1299 vector or H1299 overexpressing PDLIM4 cells into dorsal flanking sites of nude mice. Two weeks later, mice were treated with DOX (3 mg/kg, three times per week) for 2 weeks. **K** Quantitative results for Fig. 6J. Data represented mean ± SEM. **p < 0.01. **L** Western blot was used to detect the expression of p21. **M** Quantitation data for **L**. **p < 0.01. **N**, **O** Real-time PCR and IHC staining were used to examine the expression of p21. 100 × plots with a scale bar of 200 μm. Data represented mean ± SEM. **p < 0.01, *p < 0.05. All the experiments were repeated at least three times

Increased p21 responsiveness is one of the most important mechanisms of cellular chemoresistance. Given that BRD4-PDLIM4 stabilizes the p21 RNA, we next explored whether the BRD4-PDLIM4-p21 pathway could respond to chemotherapy. Compared to controls, when cells were treated with the chemotherapeutic drug doxorubicin (DOX, 0.5 μM, 2 h), both BRD4 protein and RNA levels were significantly elevated, and its downstream PDLIM4 and p21 levels were elevated accordingly (Fig. 6A, B, Supplemental Fig. 6A, B). When cells were pretreated with JQ1 or BRD4 was knocked down by shRNA, the elevation of the protein and RNA levels of PDLIM4 and p21 was inhibited upon addition of DOX (Fig. 6C, D, E, F, Supplemental Fig. 6C, D, E, F). After stable knockdown of PDLIM4 using shRNA and subsequent treatment with DOX, the protein and RNA levels of p21 were not elevated (Fig. 6G, H, Supplemental Fig. 6G, H). Collectively, these results indicated that chemotherapeutic agents can activate the BRD4-PDLIM4-p21 pathway.

We further evaluated whether PDLIM4 regulates chemosensitivity through modulation of p21. Overexpression of PDLIM4 caused hypersensitivity to DOX in LUAD cells (Fig. 6I, Supplemental Fig. 6I). Tumor xenograft models were used to further examine the role of the BRD4-PDLIM4-p21 axis in regulating sensitivity to chemotherapy in vivo. Consistent with the in vitro results, enhanced chemosensitivity to DOX was observed in PDLIM4-overexpressing LUAD cells (Fig. 6J, K, Supplemental Fig. 6J, K). IHC staining showed that the protein levels of BRD4, PDLIM4, and p21 increased following DOX treatment (Supplemental Fig. 6P, Q). Since increased p21 is the underlying cause contributing to enhanced sensitivity to tumor drugs, we examined p21 expression using Western blot, real-time PCR, and IHC staining in xenograft tumors. Protein and RNA levels of p21 were significantly upregulated after overexpression of PDLIM4 and DOX treatment (Fig. 6L, M, N, O, Supplemental Fig. 6L, M, N, O). Taken together, these data demonstrate that BRD4-PDLIM4 promotes p21 expression to diminish chemoresistance in LUAD tumor cells.

### PDLIM4 S116 mutation displays loss of function in regulating p21 expression

**Fig. 7:**
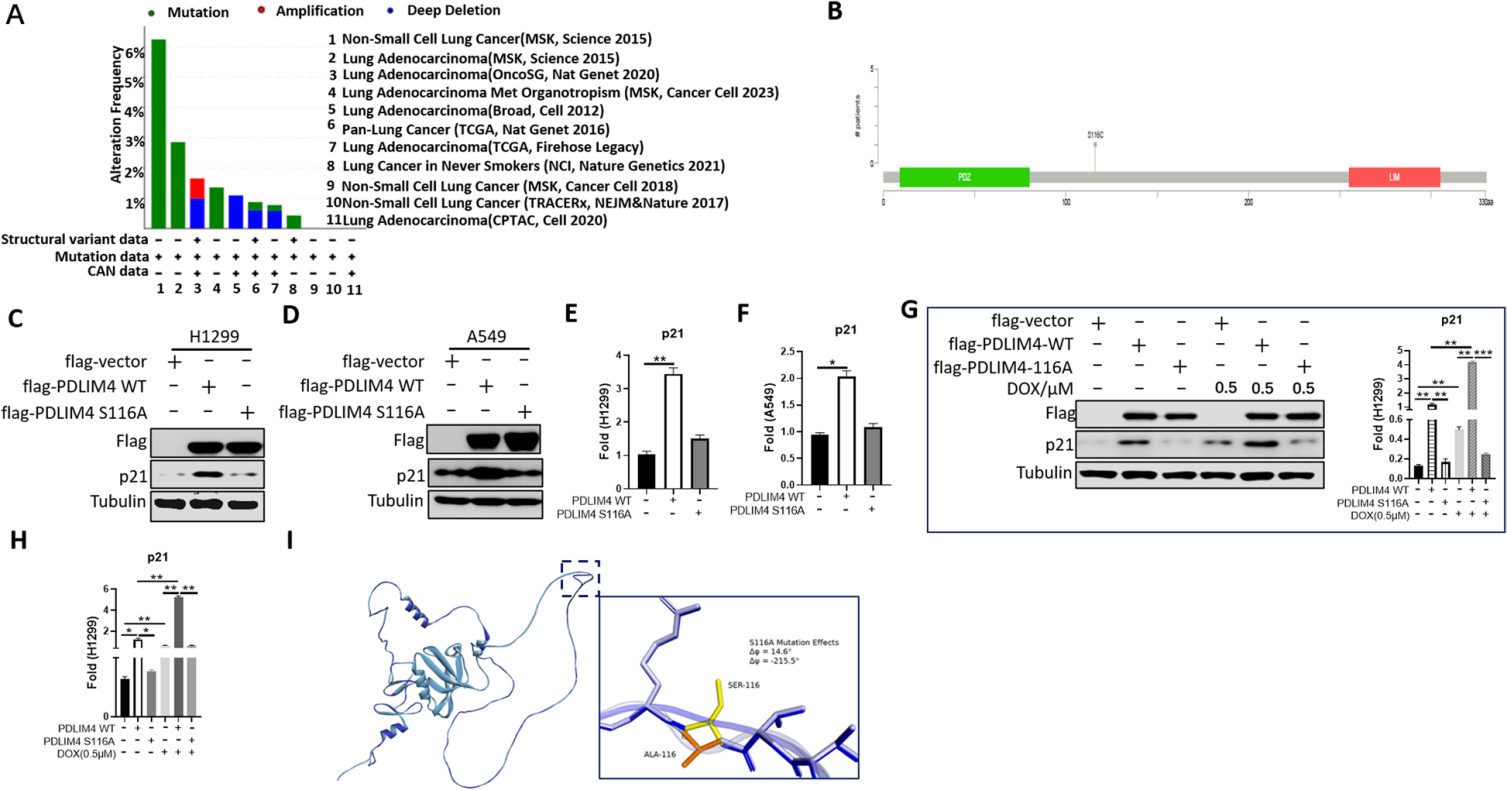
Mutant PDLIM4 S116 site losses control of p21 expression. **A** Summary of alterations for PDLIM4 in different cancer types form the LUAD TCGA project. The alteration types include mutation (green), amplification (red), and deep deletion (bule). **B** Distribution of the S116 point mutation of PDLIM4 in LUAD TCGA project. **C**, **D**, **E**, **F** The expression protein and mRNA levels of p21 were analyzed by Western blotting or real-time PCR in control cells (vector), stable overexpression flag-PDLIM4 cell (OV-PDLIM4), and stable overexpression flag-PDLIM4 S116A cell (OV-PDLIM4 S116A). Data represented mean ± SEM. ***p < 0.001, **p < 0.01. **G**, **H** H1299 cells were treated with 0.5 μM DOX for 2 h, and the protein (**G**, left) and mRNA (**H**) levels of p21 were detected. Quantitative data (**G**, right) represented mean ± SEM. **p < 0.01, *p < 0.05. All the experiments were repeated three times. **I** Superposition comparison of the structures of PDLIM4 wild-type and PDLIM4 S116A mutant. Structural superposition (WT, purple blue; S116A, light blue) with inset showing local conformational change

To verify the potential tumor-suppressive function of PDLIM4, we examined the genetic alteration status of PDLIM4 in LUAD tumor samples from the TCGA cohorts. Among adenocarcinoma patients, the highest alteration frequency of PDLIM4 was primarily of the “mutation” type (Fig. 7A). Specifically, the mutation rate at the 116th site (serine to cysteine) was 0.26% (Fig. 7B). Given the tumor-suppressive role and sensitizing chemotherapeutic action of PDLIM4, we are eager to know the function of the PDLIM4 mutation at the S116 site. Interestingly, after PDLIM4 was mutated from 116 from serine (S) to alanine (A), unlike overexpression of wild-type PDLIM4 (WT), which was able to regulate p21 expression, overexpression of PDLIM4 S116A lost the regulation of p21, both at the protein and RNA levels (Fig. 7C, D, E, F). Compared with PDLIM4 S116A cells, p21 accumulation was only found in cells stably overexpressing PDLIM4 (WT) (Fig. 7G, H). The rescue experiments showed that in H1299 and A549 cells overexpressing the PDLIM4 S116A mutant, the reintroduction of wild-type PDLIM4 led to an increase in p21 levels, both at the protein and RNA levels (Supplemental Fig. 7A, B), and the DOX sensitivity was also restored (Supplemental Fig. 7C, D). Although loss of function by the S116A mutation has been experimentally confirmed, it is unclear why this one amino acid substitution disrupts the RNA-stabilizing function. We downloaded the structure of the PDLIM4 protein from the AlphaFold database and used the Discovery Studio platform to mutate the residue SER116 in the linker region of the protein to alanine (ALA116). The mutated protein model is referred to as “S116A.” Our crystallographic studies show that the overall architecture of PDLIM4 remains intact (global backbone RMSD = 0.000 Å), but the S116A mutation induces a localized conformational change in the interdomain linker region (residues 113–120, *RMSD* = 0.050 Å). Specifically, the mutation site exhibits a dramatic 215.5° rotation in the *ψ* dihedral angle (from − 166.9 to − 83.6°) and a 14.6° shift in the *φ* angle, transitioning the backbone conformation from a *β*-sheet favored to an α-helix-favored state (Fig. 7I). This results in a 3.2 Å displacement of the Cα atom and subtle distortions in adjacent residues 115–117. No direct hydrogen bond partners were found within 4.0 Å of S116 in the wild-type structure. We propose that this increased flexibility may allosterically affect the positioning of the PDZ and LIM domains, explaining the altered interaction profiles observed in functional studies. Thus, the structural data provide a mechanistic basis for the mutation-induced functional changes, showing how localized linker perturbations can modulate distal interaction interfaces without compromising overall fold integrity.

**Fig. 8:**
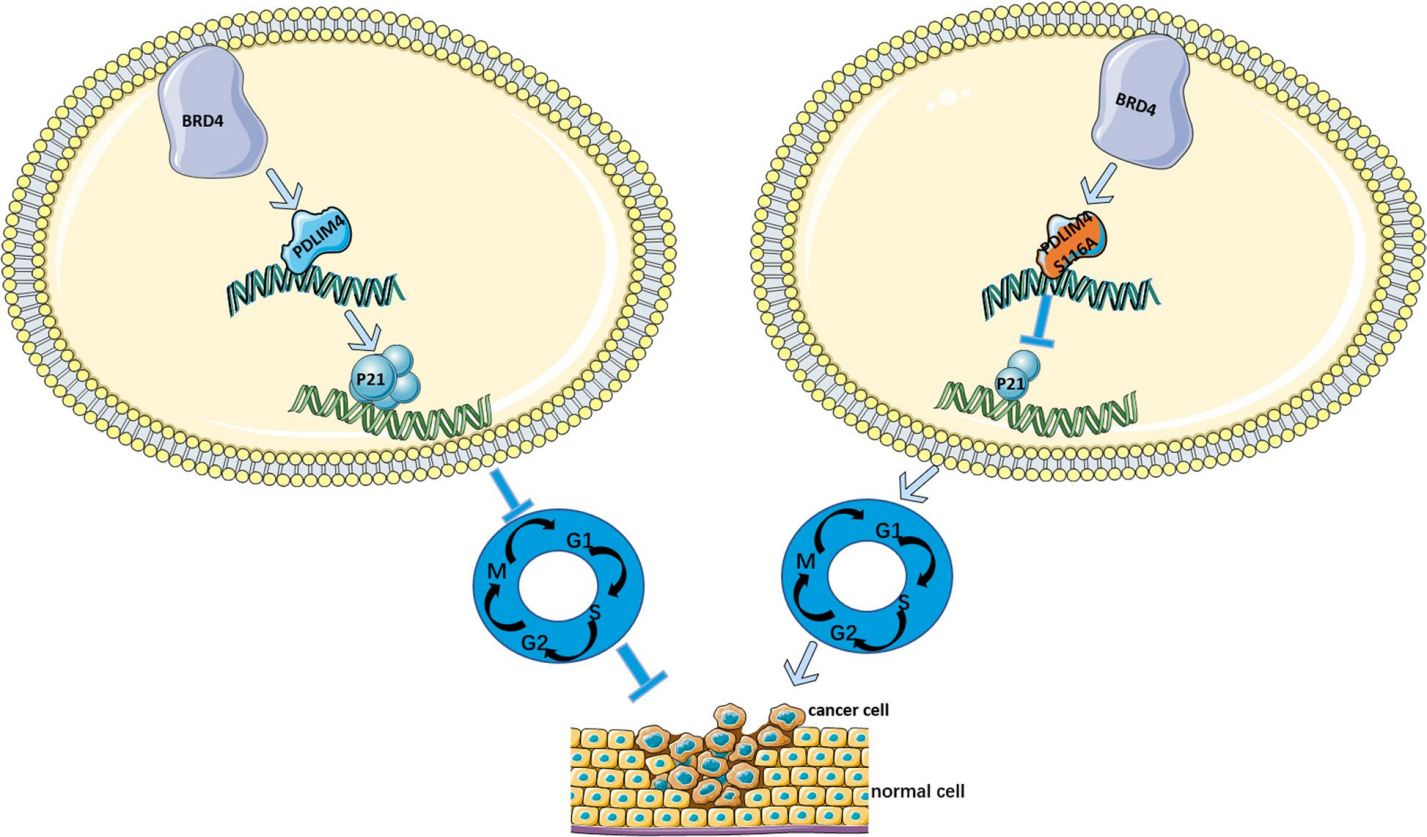
A model depicting the BRD4-PDLIM4-p21 pathway in the regulation of the cell cycle and carcinogenesis

We propose a model in which either PDLIM4 WT or the PDLIM4 S116 mutation leads to regulation of p21 levels in a p53-independent manner, thereby controlling cell proliferation (Fig. 8A). Overall, this study defines PDLIM4 as a putative tumor suppressor, and the BRD4-PDLIM4-p21 regulatory pathway significantly contributes to LUAD cell arrest and chemosensitivity.

## Discussion

PDLIM4, a member of the PDLIM family, which includes conserved PDZ and LIM domains, has been implicated in the progression of various cancers [18]. Previous studies have shown that PDLIM4 overexpression suppresses tumor growth and clonogenicity in colon cancer cells [22]. Additionally, PDLIM4 expression is reduced in prostate cancer tissues compared to adjacent benign tissues, and its loss is associated with poor prognosis in ovarian cancer [23, 24]. In this study, we investigated the role of PDLIM4 in lung adenocarcinoma (LUAD). Analysis of clinical LUAD samples and data from the TCGA database revealed that PDLIM4 is highly expressed in tumor tissues, and its upregulation correlates with better patient prognosis. Furthermore, our experimental data indicate that overexpression of PDLIM4 significantly inhibits LUAD cell proliferation and colony formation and arrests the cell cycle at the G2/M phase. In vivo, PDLIM4 overexpression leads to reduced growth of xenograft tumors derived from human LUAD cell lines, supporting the notion that PDLIM4 acts as a tumor suppressor in LUAD, as it does in other cancers like ovarian and prostate cancer. These results help explain the association between high PDLIM4 expression and favorable prognostic parameters in LUAD.

This “high expression yet tumor-suppressive” behavior may seem counterintuitive, but similar patterns have been reported: in clear cell renal carcinoma (KIRC), HHLA2 is highly expressed and associated with favorable prognosis [40], and upregulation of PD-L1 predicts better outcomes in colorectal, breast, and ovarian cancers [41]. Our findings confirm that the BRD4-PDLIM4-p21 axis is a tumor-suppressive pathway in LUAD, but its overall impact may be relatively modest and readily overshadowed by dominant oncogenic drivers. Thus, the final phenotypic output, whether tumor suppressive or tumor promoting, is complex. Our study, which focuses on LUAD and cell cycle control, does not address the role of PDLIM4 in other malignancies or biological processes, which is indeed a limitation of our research. Like other tumor suppressors, the prognostic value of PDLIM4 is likely to vary by cancer type and depend on multiple upstream and microenvironmental signals. Future work will dissect its upstream regulators, downstream substrates, and functions across diverse tumor contexts to fully elucidate its complex role in cancer biology.

The progression of the cell cycle is regulated by cyclins and cyclin-dependent kinases (CDKs), with key transitions controlled by the activation of these CDKs [42, 43]. P21, a universal inhibitor of cyclin-CDK complexes, inhibits the activity of Cdk2/cyclin E complexes, leading to G1/S and G2/M phase arrest [38, 44]. P21 transcription is commonly regulated by the tumor-suppressor gene p53 [38]. However, p53 is mutated or absent in approximately 50% of human cancers, including more than 80% of LUAD cases; in these cases, p53 loses its regulatory control over p21 [9–11]. Thus, identifying novel upstream regulators of p21 is an important area of cancer research. Our findings provide evidence that PDLIM4 is a p53-independent regulator of p21 in LUAD, but further studies are needed to elucidate how PDLIM4 prevents p21 mRNA degradation.

This study shows that the BRD4-PDLIM4-p21 axis is activated by 0.5-µM doxorubicin treatment for 2 h in A549 or H1299 cells and concomitantly increases tumor chemoresistance. Notably, the biological output of p21 is exquisitely dose and context dependent: in colorectal cancer models, low-dose doxorubicin relies on p21 to preserve mitotic fidelity, whereas high-dose treatment converts p21 into an obligatory pro-apoptotic factor [45]; in hepatocellular carcinoma, the same dose gradient dictates a binary cell-fate decision, with low concentrations precipitating mitotic catastrophe and high concentrations triggering canonical apoptosis [46]. Multiple reports characterize p21 as a prototypical double-edged sword during chemotherapy. On one side, it serves as a canonical cell-cycle brake: under conditions of intact p53 or robust DNA-damage signaling, nuclear p21 inhibits cyclin-CDK complexes and cooperates with PCNA to facilitate DNA repair, thereby sensitizing tumor cells to agents such as cisplatin [47]. On the other side, sustained cytoplasmic retention of p21, especially in p53-null backgrounds or when the PI3K/Akt pathway is hyperactivated, can enforce an irreversible senescence-like arrest, blunt caspase activation, or bolster STAT3/NF-κB prosurvival circuits, ultimately conferring resistance to cisplatin, 5-FU, and related drugs [48]. These findings underscore the urgent need to stratify patients according to p21 functional status and to develop precision interventions that manipulate p21 nucleo-cytoplasmic trafficking or selectively target oncogenic p21 subpopulations, thereby recalibrating the balance between chemosensitization and resistance reversal.

Bromodomain-containing protein 4 (BRD4) is a key member of the bromodomain and extraterminal domain (BET) family, which binds to acetylated lysine residues on histone tails and regulates gene transcription [27]. BRD4 has been implicated in regulating genes important for cell growth and survival, including MYC, BCL2, CCND1, and p53 [30–36]. Our results show that BRD4 can directly bind to the promoter of PDLIM4, leading to increased PDLIM4 RNA levels. Knockdown of BRD4 or treatment with the BET inhibitor JQ1 decreased PDLIM4 RNA levels. Further investigation revealed that the BD1 domain of BRD4 plays a critical role in regulating PDLIM4 expression. Importantly, we found that BRD4 regulates p21 expression through PDLIM4, and both endogenous and exogenous experiments showed that chemotherapeutic treatment elevated BRD4, which in turn promoted p21 expression via PDLIM4, enhancing chemosensitivity in LUAD cells. In order to exclude that BRD4 plays a regulatory role in p21 through p53 [49, 50], we verified all the conclusions in H1299 (p53-deficient) and A549 (expressing wild-type p53) cells. Therefore, we suggest that BRD4-PDLIM4-p21 is a potential natural tumor suppressive pathway in tumor cells independent of p53.

Interestingly, our analysis of LUAD samples from the TCGA database revealed a significant number of mutations in PDLIM4, including the serine at position 116. Although the S116 mutation in PDLIM4 is relatively uncommon in LUAD (0.26%, less than 1%), the results presented in Fig. 7C, D, E, and F and G and H indicate that this mutation may lead to the loss of PDLIM4’s regulatory function over p21 while also causing the loss of tumor-suppressive activity and the development of chemotherapy resistance. These findings offer valuable insights for the therapeutic strategies and prognosis of patients with the S116 mutation. Given the large number of patients with lung adenocarcinoma, even a mutation frequency as low as 0.26% corresponds to a substantial number of cases. Therefore, our discovery holds considerable clinical value and significance. Although it remains to be verified and explored whether the mutant PDLIM4 is directly regulated by BRD4 in cancer cells and whether it has functions such as enhanced invasion, motility, transcriptional inhibition, or activation, our research suggests that the study of PDLIM4 mutants has the potential to promote cancer. Future studies will integrate these signal pathways to elucidate PDLIM4 functions in LUAD carcinogenesis. Comprehensive analysis of TCGA data reveals that PDLIM4 is consistently elevated across all stages of lung adenocarcinoma (LUAD) (figure not shown), without significant stage-dependent fluctuations. Our functional assays conducted in both patient-derived tissues and murine xenograft models demonstrate that this elevated PDLIM4 promotes the accumulation of p21, thereby exerting a cell cycle-arresting effect.

## Conclusions

In summary, our data show that PDLIM4 is upregulated in LUAD and correlates with improved survival in patients. Overexpression of PDLIM4 significantly enhances proliferation, tumorigenesis, and chemotherapy sensitivity in LUAD. Mechanistic analysis reveals that wild-type PDLIM4, but not its mutant form, regulates the mRNA level of p21. Furthermore, we identify BRD4 as a novel positive regulator of PDLIM4, with the BRD4-PDLIM4-p21 pathway exerting anti-tumor effects in LUAD. These findings suggest that PDLIM4 could serve as a promising therapeutic target for LUAD.

## Methods

### Antibodies and reagents

All primary antibodies were diluted with 3% bovine serum albumin in PBS containing 0.5% Tween 20 for Western blot analysis. Primary antibodies which included anti-GAPDH (Cat. No. 60004–1-1 g), anti-BRD2 (Cat. No. 22236–1-AP), anti-BRD3 (Cat. No. 11859–1-AP), anti-BRD4 (Cat. No. 67374–2-1 g), anti-TUBULIN (Cat. No. 10094–1-AP), anti-FLAG (Cat. No. 66008–4-Ig), anti-HA (Cat. No. 51064–2-AP), anti-GFP (Cat. No. 50430–2-AP), and anti-P21 (Cat. No. 10355–1-AP) were purchased from Proteintech Group; anti-PDLIM4 (Cat. No. A20463) was purchased from ABclonal. Peroxidase-conjugated anti-mouse secondary antibody (SSA004) and anti-rabbit secondary antibody (SSA007) were purchased from SinoBiological. Doxorubicin (Cat. No. HY-15142) and AZD5153 (Cat. No. 1869912–39-9) were purchased from MedChemExpress; JQ1 (Cat. No. SML1524-5MG) was purchased from Sigma.

### Plasmids

shRNA for BRD2 (shBRD2, Cat. No. L18324), BRD3 (shBRD3, Cat. No. L18325), BRD4 (shBRD4, Cat. No. L18326), PDLIM4 (shPDLIM4-1, Cat. No. L18321), and PDLIM4 (shPDLIM4-2, Cat. No. L16578) were purchased from YoubaoBio. 3xflag-BRD4 (Cat. No. P1162) was obtained from MiaolingBio, China. GFP-BRD4-BD1 and GFP-BRD4-BD2 were generated by cloning the corresponding cDNA into the pcdna3.0 vector. All shRNA sequences are shown in Supplementary Table 1.

### Cell culture

Human renal epithelial cell line 293 T and lung adenocarcinoma cell lines A549 and H1299 were purchased from the American Type Culture Collection (ATCC, Rockville, MD, USA) and cultured according to the ATCC’s directions.

### Western blot

Cells were homogenized in cold RIPA lysis buffer (50 mM Tris–HCl pH 7.4, 1 mM EDTA, 150 mM NaCl, 1% NonidetP-40, 0.25% sodium deoxycholate, 0.1% SDS and protease inhibitors) and SDS-PAGE gels subsequently. After transfer to PVDF membranes and blocking with 5% skim milk, immunoblots were analyzed using primary antibodies at 4 °C overnight. They were then incubated for 1 h with the corresponding HRP-conjugated secondary antibodies and detected using ImageQuant LAS 500 or Tanon 5200.

### RNA extraction and quantitative PCR assays

Total RNA was extracted using AG RNAex Pro Reagent (Accurate Biotechnology) and dissolved in RNase-free water. Reverse transcription was performed using HiScript II Q RT SuperMix for cDNA. Reverse transcription was performed using HiScript II Q RT SuperMix for cDNA, and quantitative PCR assays were carried out by using ChamQ Universal SYBR qPCR Master Mix (Vazyme). All the primers (BRD4, PDLIM4, p21, GAPDH, and so on) were purchased from Tsingke. The GAPDH gene was used as an internal reference gene. Each experiment was repeated three times. Primer sequences for quantitative PCR assays are shown in Supplementary Table 2.

### Construction of stable expression cells

The PDLIM4 coding sequence was found on the website of the National Center for Biotechnology Information. PLVX-EF1a-IRES-Puro was selected as the vector, and Primer Premier 5 software was used to design suitable PCR primers with FLAG protein sequences according to the PDLIM4 sequence and the vector cleavage site. The PDLIM4 fragment was amplified by PCR and integrated into the vector. The vector containing the PDLIM4 fragment, the purchased PLVS-shPDLIM4-PURO, and the packaging vectors (pCMV-dR8.9 and pCMV-VSV-G) were transiently transfected into 293 T cells. The supernatant containing the *Lentivirus* was collected after incubation at 37 °C for 72 h. After infecting the lung adenocarcinoma cell lines A549 and H1299 with virus-containing supernatant for at least 48 h, stable expression cell lines were obtained by screening with puromycin at a concentration of 1 μg/mL for at least 48 h.

### Flow cytometry analysis

Lung adenocarcinoma cell lines A549 and H1299 were cultured in 6-well plates for 48 h, For detecting the proportion of cells at each stage, lung adenocarcinoma cell lines A549 and H1299 were stained with propyl iodide in 37 °C incubator away from light for 30 min. Flow cytometry was used to detect red fluorescence (PE channel) at the excitation wavelength of 488 mm, and cells were obtained at a low speed, which ensured that the differences between cells were as small as possible in order to ensure higher accuracy. The DNA content of cells was analyzed using appropriate analysis software such as ModFit software.

### Chromatin immunoprecipitation (ChIP) assay

Nuclear proteins were crosslinked to genomic DNA after 1% formaldehyde treatment for 10 min. Crosslinking was stopped by adding 0.125-M glycine and incubating for 5 min at room temperature on a rocking platform. Then, the cells were washed twice with cold PBS (137 mM NaCl, 2.7 mM KCl, 10.1 mM Na2HPO4, and 1.8 mM KH2PO4). The cells were collected by scraping in solution A (10 mM Tris–HCl (pH 7.9), 0.25% Triton-X 100, 10 mM EDTA, and protease inhibitor cocktail). After centrifugation, the cell pellets were resuspended in solution C (50 mM Tris–HCl (pH 7.5), 150 mM NaCl, 0.1% SDS, 1% Triton-X 100, 1 mM EDTA, 0.1% deoxycholic acid sodium, 2 mM PSMF, and protease inhibitor cocktail). Then, the cross-linked chromatin was sheared by sonication, providing fragments of 200–1000 bp in length. Cellular debris was removed by centrifugation, and salmon sperm DNA (final concentration of 200 μg/mL), 50 μL of protein A/G agarose, and BSA (1 mg/mL) slurry were added to the lysates for 2 h at 4 °C with agitation to remove nonspecific background. After centrifugation, the supernatant was collected to incubate with 3–5 μg of the indicated antibodies, 8–10 μL of protein A/G agarose, and BSA (1 mg/mL) overnight at 4 °C with rotation. The beads were pelleted by centrifugation for 1 min at 4 °C and washed successively with the following buffers at 4 °C for 10 min by rotation: chip 1 buffer (50 mM Tris–HCl (pH 7.5), 150 mM NaCl, 1% Triton-X 100, 2 mM EDTA, 0.1% deoxycholic acid sodium and 1 mM DTT), chip 2 buffer (50 mM Tris–HCl (pH 7.5), 500 mM NaCl, 1% Triton-X 100, 2 mM EDTA, 0.1% deoxycholic acid sodium, and 1 mM DTT), chip 3 buffer (10 mM Tris–HCl (pH 8.0), 250 mM LiCl, 0.5% NP40, 1 mM EDTA, 0.5% deoxycholic acid sodium, and 1 mM DTT), and TE buffer (1 mM EDTA and 10 mM Tris–HCl (pH 8.0)). The immunocomplexes were then incubated with 200-μL elution buffer (0.5% SDS, 0.1 M NaHCO3, and 5-μg protein K) and incubated at 65 °C with rotation for 6 h. The DNA was extracted by phenol/chloroform/isoamyl alcohol (25/24/1) extractions and precipitated with 3 volumes of ethanol and 0.1 volume of 3-M sodium acetate (pH 5.2) using glycogen as a carrier. PCR amplification of the genomic DNA was performed with specific primers. The UCSC Genome Browser was used to find the CpG domain near the transcription start site (TSS) on chr5:132,249,816–132,281,333 (GRCh38). Then, the CpG domain sequence was obtained and used to design the chip primers for the PDLIM4 promoter by primer 5 software. Human PDLIM4 primers are as follows: forward, 5′-CCCAGATCCCCTGTGTATCC-3′ and reverse, 5′-AAGCCGCCTCTACAGCATG-3′.

### Immunohistochemical (IHC) staining and scoring

Tissues were fixed in 4% paraformaldehyde and embedded in paraffin wax for histological examination which cut into 5-μm thick slices and stained with haematoxylin and eosin. For immunohistochemistry, the tissue sections were quenched endogenous peroxidase with 3% hydrogen peroxide and sealed with 3% fetal bovine serum before incubating with specific primary antibodies, followed by HRP-conjugated secondary anti-rat or anti-rabbit antibody. Diaminobenzidine was used for staining and terminated with water. Lastly, counterstaining was performed with hematoxylin. The staining results were evaluated by two pathologists without knowledge of the clinical outcome. The staining intensities were graded from 0 to 3, where 0 was defined as negative, 1—weak, 2—moderate, and 3—strong. The percentage of immune-reactive cells was graded from 1 to 4. Specifically, < 25% of cells stained as 1, 25–50% as 2, 50–75% as 3, and > 75% as 4. The total score was determined as the product of staining intensity and percentage scores, ranging from 0 to 12. An IHC score of 6 or greater was considered high expression, while an IHC score of < 6 was regarded as low expression.

### Cell proliferation assay

Lung adenocarcinoma stably transfected cell lines (A549 and H1299) were cultured for 24, 48, or 72 h in a 96-well plate. The medium was removed, and the new medium and 20 μL of Cell Counting Kit‐8 reagents were added. The cells were incubated at 37 °C for 1 h, then the absorbance was measured using Microplate Manager 6 at a wavelength of 450 nm, and statistical analysis was performed. All experiments were performed in triplicate.

### Colony-forming assay

The clone formation rate reflects the ability of cell proliferation. Cells that grew to the logarithmic stage were digested with 0.25% trypsin. A total of 200–500 cells were cultured in a 6-well plate for about 1 week. The culturing was stopped when the right size clones were observed in the 6-well plate. Cells were washed twice with PBS after removing the medium, then fixed with 1 mL of 4% paraformaldehyde for 20 min, and stained with 0.5% crystal violet for 15 min after the fixative was removed. Cells were washed with water several times until the stain was clean after removing the 0.5% crystal violet and then dried in air. The clones on the 6-well plate were photographed and compared.

### Xenograft tumorigenicity analysis

Male BALB/c-nu mice (nude mice) were purchased from Rise Mice Biotechnology Co., Ltd. (Zhaoqing, China) and SCXK (Guangdong) 2025–0053. Lung adenocarcinoma cell lines A549 and H1299, stably expressing target genes, were cultured to the logarithmic growth phase and then digested with trypsin. After washing twice with PBS, each male BALB/c-nu mouse was subcutaneously inoculated with × 10^6^ cells in the axillary region. Half of the mice in each treatment group received intraperitoneal injections of doxorubicin at a dose of 8 mg/kg body weight. Subcutaneous tumor growth was monitored every 2–3 days by measuring the major and minor axes with vernier calipers. Tumor-bearing mice were euthanized when the longest tumor diameter reached 20 mm, defined as the “tumor endpoint.” Mice without tumor development were euthanized at the end of the study period, defined as the “cure endpoint.” Following euthanasia, tumor tissues were collected for measurement and comparative analysis. All anesthesia and euthanasia procedures were strictly performed in accordance with the Guide for the Care and Use of Laboratory Animals and in accordance with the recommendations of the International Veterinary Association. All protocols were reviewed and approved by the Institutional Animal Care and Use Committee (IACUC) of the Affiliated Hospital of Guangdong Medical University (Protocol Approval No.: AHGDMU-LAC-B-202410–0256). Mice were deeply anesthetized in an induction chamber with 5% isoflurane until complete unresponsiveness was confirmed. While under deep anesthesia, euthanasia was immediately performed via cervical dislocation. Death was verified by the absence of spontaneous respiration.

### 5-Ethynyl-2′-deoxyuridine (EdU) and an immunofluorescence assay

The lung adenocarcinoma cells in the logarithmic growth stage were inoculated into the 6-well plate after digestion so that the cells were uniformly attached to the cover glass in the 6-well plate. Transfection was performed when the cell density reached 60%. The cells were then continuously cultured at 37 °C for 24 h. For the 5-ethynyl-2′-deoxyuridine (EdU) assay, the cells were incubated with 50 μM EdU and cultured for 6 h. A total of 50 μL of 4% paraformaldehyde was used to fix the cells at room temperature for 30 min. The cells were penetrated by adding 200-μL penetrant (PBS of 0.5% Triton X-100) for 30 min. The cells were incubated for 10 min with DAPI solution away from light, then the cover glass was placed on the slide, and the proliferating cells were observed under a confocal fluorescence microscope. The proportion of EdU-positive cells was compared and statistically analyzed. Cells were sealed with 3% fetal bovine serum after fixation and penetration for the immunofluorescence assay. Cells were incubated with a specific primary antibody at 4 °C overnight and with the corresponding secondary antibody for 1 h away from light. The cover glass was placed on the slide, and the proliferating cells were observed under a confocal fluorescence microscope.

### RNA sequencing

Total RNA isolated from *BRD4* overexpression and control cells was qualitatively assessed before RNA sequencing (RNA-seq) library construction. The RNA-seq library was sequenced on an Illumina HiSeq platform. Adaptor sequences were trimmed from the raw reads, and sequencing quality was evaluated with FastQC software (version 0.11.2). Quality scores, sequence duplication, adaptor content, and other metrics were used to determine whether additional filtering was needed before genome mapping. Clean reads were mapped onto the reference genome (GRCh38.p13) with the HISAT2 alignment program (version 2.1.0). The mappable reads were assembled into transcripts or genes with the StringTie transcriptome assembler (version 1.3.5). Only coding genes were retained for further analysis. To identify genes differentially expressed between *BRD4* overexpression and control cells, we conducted a differential gene expression analysis using the DESeq2 package (version 1.28.1) in R. Fold changes were log2-transformed, and adjusted *p*-values (padj) were calculated using the Benjamini–Hochberg procedure to control the false discovery rate. Genes in the *PDLIM4* overexpression cells were considered to be significantly upregulated if the log2 fold-change was ≥ 1 and the padj was < 0.05 or considered to be significantly downregulated if the log2 fold-change was ≤ − 1 and the padj was < 0.05. Differentially expressed genes were selected for further analysis.

### Bioinformatics analysis

Mutational data from NSCLC patients were retrieved from the TCGA dataset using the cBioportal portal (http://www.cbioportal.org/). Survival analysis and Kaplan–Meier representations were performed using R version 3.5.1 and the package “Survival.” A log-rank test was applied to compare survival curves and calculate *p*-values.

Gene Expression Profiling Interactive Analysis 2 (GEPIA2; http://gepia2.cancer-pku.cn/) was used to evaluate the differential expression of PDLIM4 between norma and LUAD tissues. A total of LUAD samples with RNA-seq data, together with the corresponding clinical information, were derived from the TCGA database (https://portal.gdc.com). PDLIM4 gene expression levels from CPTAC were obtained from UALCAN portal (http://ualcan.path.uab.edu).

### Statistical analysis

All data were shown as mean ± SEM of at least three independent experiments. Statistical analyses were performed using the help of GraphPad Prism software (version 8.0). The differences between groups were analyzed using two Student’s *t*-test or one-way ANOVA. Statistical significance was indicated as follows: n.s., no significance, *p < 0.05, **p < 0.01, and ***p < 0.001.

## Supplementary Information

Additional file 1: Figure S1. Stable overexpression or knockdown PDLIM4 cells were constructed. Figure S2. PDLIM4 inhibits proliferation and arrests cell cycle in LUAD cells. Figure S3. BRD4 controls the expression of PDLIM4. Figure S4. PDLIM4 co-localizes with p21 at various period during cell cycle. Figure S5. The regulation of the BRD4/PDLIM4/p21 axis in LUAD cells. Figure S6. BRD4-PDLIM4-p21 pathway sensitizes cells to chemotherapy. Figure S7. Rescue experiment. Table S1. All shRNA and primer sequences used in this paper. Table S2. Sequences of homo primers used for RT-PCR. Original Data.

Additional file 2: Table S3. Potential target genes.

Additional file 3: Table S4. 712 genes showing upregulated expression trends upon BRD4 overexpression.

## Data Availability

All data generated or analyzed during this study are included in this published article and its supplementary information file. The primer information and unprocessed images of WB are included in Additional file 1.

## Abbreviations

LUAD: Lung adenocarcinoma
BET: Bromodomain and extraterminal
TCGA: The Cancer Genome Atlas
CPTAC: The Clinical Tumor Proteomics Analysis Consortium database
EdU: 5-Ethynyl-2′-deoxyuridine
BETi: BET bromodomain inhibitors
Act D: Actinomycin D
DOX: Doxorubicin
