# Supplementary material for: BRD4-mediated transcriptional activation of PDLIM4 enhances p21 stability and chemosensitivity in lung adenocarcinoma independent of p53
Source: BMC Biol. 2026 Jan 17;24:37. doi: 10.1186/s12915-026-02511-z (PMC12896191; doi:10.1186/s12915-026-02511-z)

Supplementary Figure Legends 1-7

Figure S1. Stable overexpression or knockdown PDLIM4 cells were constructed. **A-B** Western blot analysis of the expression of PDLIM4 protein in H1299 or A549 cells transduced with virus expression flag-PDLIM4 or flag-vector. **C** Western blot analysis of the expression of PDLIM4 protein in 293T cell transduced with lentivirus expression PDLIM4 shRNA1#, shRNA 2# or control shRNA. The relative protein levels quantified by gray value and normalized to tubulin. **D-E** Western blot analysis of the expression of PDLIM4 protein in H1299 or A549 cell transduced with lentivirus expression PDLIM4 shRNA1# or control shRNA. The relative protein levels quantified by gray value and normalized to tubulin. ***P <0.001, *P <0.05.

Figure S1

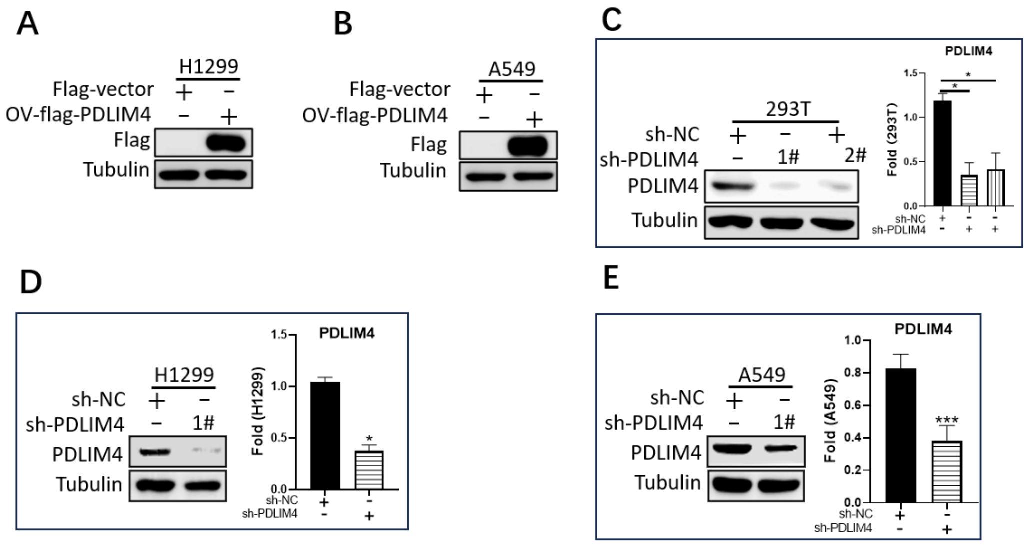

Figure S2. PDLIM4 inhibits proliferation and arrests cell cycle in LUAD cells. A-B The representative images (lower) and colony numbers quantitation (>1mm, upper) were performed in A549 stable overexpression of PDLIM4 (OV- PDLIM4) or PDLIM4 depletion (sh-PDLIM4) cell lines. Data are represented as the mean ± SEM. **P <0.01. **C-D** CCK-8 assays were displayed after overexpression of PDLIM4 or PDLIM4 depletion in A549 cells. Data are represented as the mean ± SEM. **P <0.01, *P <0.05. **E** Stable overexpression of PDLIM4 in A549 cell lines were stained with propidium iodide (PI) and the Flow cytometry assayed for cell cycle. *P <0.05. **F** In vivo tumorigenicity in nude mice evaluated by subcutaneous injection of NC and OV-flag-PDLIM4 H1299 cells, showing representative images of xenograft (left) and the statistical results of tumor volume(right). Data represented mean ± SEM (Analysis of Variance, n=6, **P<0.01). **G** Representative images of HE and IHC (left) and statistical results (right) were presented by Ki67 or PCNA staining of Figure 2G. All data in this figure are representatives of three independent repeats. **H** The volcano plot of the differently expressed genes in RNA-seq. **P <0.01, *P <0.05.

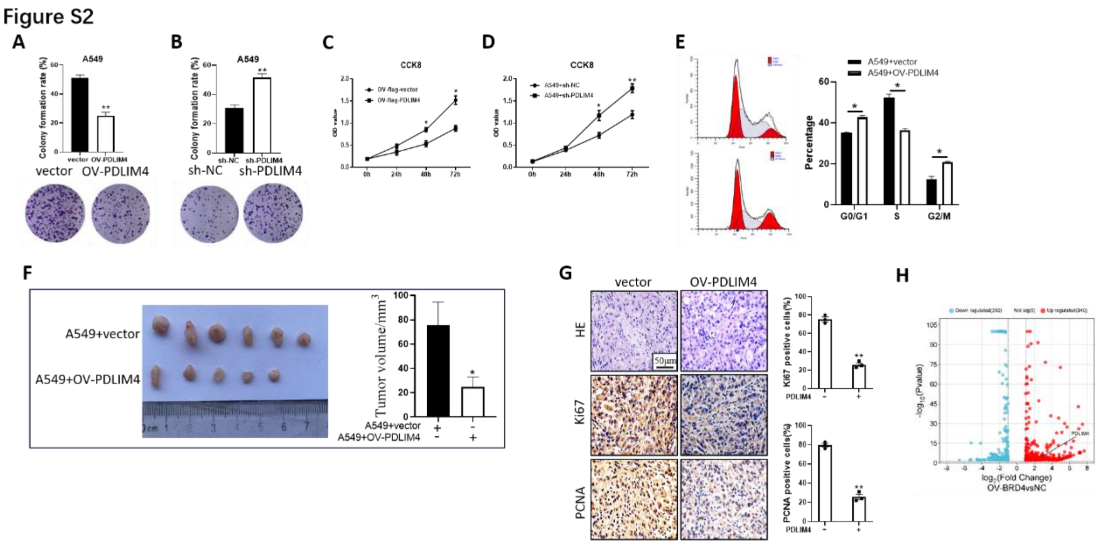

Figure S3. BRD4 controls the expression of PDLIM4. A Western blot analysis of the expression of PDLIM4 in A549 cells treated with 1μM JQ1 for 12 to 48h. Data are represented as the mean ± SEM. *P < 0.05. **B** Real-time PCR analysis of the expression of PDLIM4 in A549 cells treated with 1μM JQ1 for 6 to 24h. Data are represented as the mean ± SEM. *P < 0.05. **C** Western blot analysis of the expression of PDLIM4 in A549 cells treated with the indicated concentration of JQ1 for 48h.**D** Real-time PCR analysis of the expression of PDLIM4 in A549 cells treated with the indicated concentration of JQ1 for 48h. Data represented mean ± SEM **P < 0.01, *P < 0.05. **E** Western blot (right) and Rear-time PCR (left) analysis of the expression of PDLIM4 in H1299 and A549 cells treated with the indicated concentration of AZD5153 12h for 48h. **F** Western blot (right) and Rear-time PCR (left) analysis of the expression of PDLIM4 in H1299 and A549 cells treated with the indicated concentration of AZD5153 for 48h. **G-L** Real-time PCR analysis of the expression of BRD2, BRD3 and BRD4 respectively in H1299 or A549 cells after stable knockdown of BRD2 or BRD3 or BRD4. **M-P** Western blot analysis of the expression of PDLIM4 in H1299 or A549 cells after stable knockdown of BRD2 or BRD3. **Q** The expression of PLDIM4 was detected by real-time PCR after stable knockdown of BRD2 or BRD3 or BRD4 in A549 cells. Western blot analysis of PDLIM4 expression in BRD4 knockdown cells. **S** Real-time PCR analysis of the expression PDLIM4 in BRD4 stable knockdown cells. **T-W** Western blot (right) and Rear-time PCR (left) analysis of the expression of PDLIM4 in H1299 and A549 cells Single knockdown of BRD2/BRD3 or dual knockdown of BRD2/3 and BRD4. **X** A549 cells were treated with either JQ1 for 48h or overexpression BRD4 as indicated and harvested for ChIP assay subsequently. **Y** Western blot analysis of the expression of PDLIM4 after transient transfection of BRD4-BD1 or BRD4-BD2 in A549 cells. Data are represented as the mean ± SEM. *P < 0.05. Data are represented as the mean ± SEM. **P < 0.01. All the experiments were repeated at least three times.

Figure S3

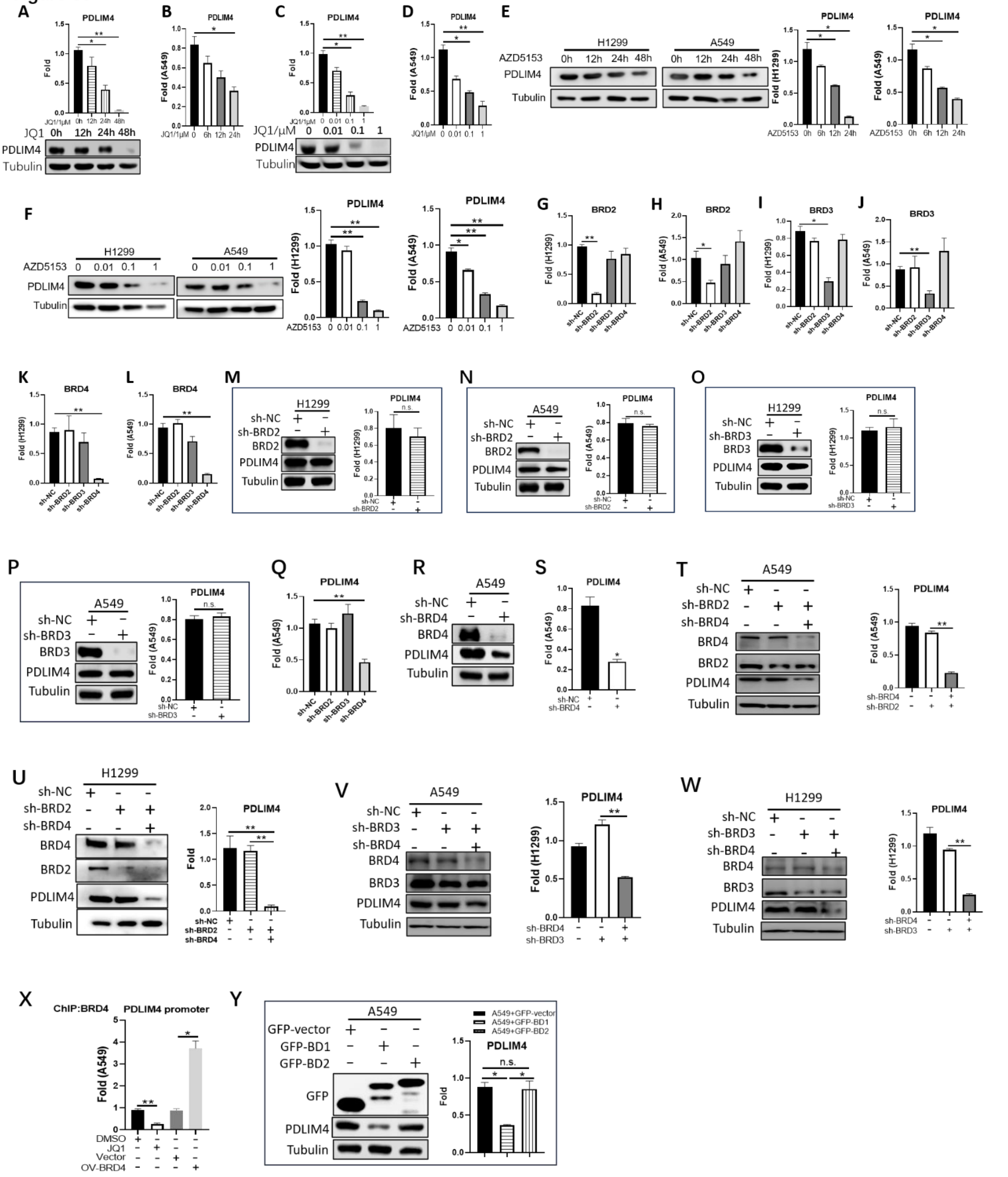

A
PDLIM4
Fold
JQ1 0h 12h 24h 48h
PDLIM4
Tubulin
B
PDLIM4
Fold (A549)
C
PDLIM4
Fold
JQ1/μM 0 0.01 0.1 1
PDLIM4
Tubulin
D
PDLIM4
Fold (A549)
E
H1299
A549
AZD5153 0h 12h 24h 48h 0h 12h 24h 48h
PDLIM4
Tubulin
PDLIM4
Fold (H1299)
PDLIM4
Fold (A549)
F
H1299
A549
AZD5153 0 0.01 0.1 1 0 0.01 0.1 1
PDLIM4
Tubulin
PDLIM4
Fold (H1299)
PDLIM4
Fold (A549)
G
BRD2
Fold (H1299)
H
BRD2
Fold (A549)
I
BRD3
Fold (H1299)
J
BRD3
Fold (A549)
K
BRD4
Fold (H1299)
L
BRD4
Fold (A549)
M
H1299
sh-NC
sh-BRD2
BRD2
PDLIM4
Tubulin
PDLIM4
Fold (H1299)
N
A549
sh-NC
sh-BRD2
BRD2
PDLIM4
Tubulin
PDLIM4
Fold (A549)
O
H1299
sh-NC
sh-BRD3
BRD3
PDLIM4
Tubulin
PDLIM4
Fold (H1299)
P
A549
sh-NC
sh-BRD3
BRD3
PDLIM4
Tubulin
PDLIM4
Fold (A549)
Q
PDLIM4
Fold (A549)
R
A549
sh-NC
sh-BRD4
BRD4
PDLIM4
Tubulin
S
PDLIM4
Fold (A549)
T
A549
sh-NC
sh-BRD2
sh-BRD4
BRD4
BRD2
PDLIM4
Tubulin
PDLIM4
Fold (A549)
U
H1299
sh-NC
sh-BRD2
sh-BRD4
BRD4
BRD2
PDLIM4
Tubulin
PDLIM4
Fold
V
A549
sh-NC
sh-BRD3
sh-BRD4
BRD4
BRD3
PDLIM4
Tubulin
PDLIM4
Fold (H1299)
W
H1299
sh-NC
sh-BRD3
sh-BRD4
BRD4
BRD3
PDLIM4
Tubulin
PDLIM4
Fold (H1299)
X
ChIP:BRD4 PDLIM4 promoter
Fold (A549)
DMSO
JQ1
Vector
OV-BRD4
Y
A549
GFP-vector
GFP-BD1
GFP-BD2
GFP
PDLIM4
Tubulin
A549+GFP-vector
A549+GFP-BD1
A549+GFP-BD2
PDLIM4
Fold

Figure S4. PDLIM4 co-localizes with p21 at various period during cell cycle. A Immunofluorescence stained endogenous PDLIM4(green) and p21(red) in H1299 cells were visualized by confocal microscopy. DAPI staining was included to visualize the cell nucleus(blue). DAPI, 4′,6-diamidino-2 phenylindole. All the experiments were repeated at least three times.

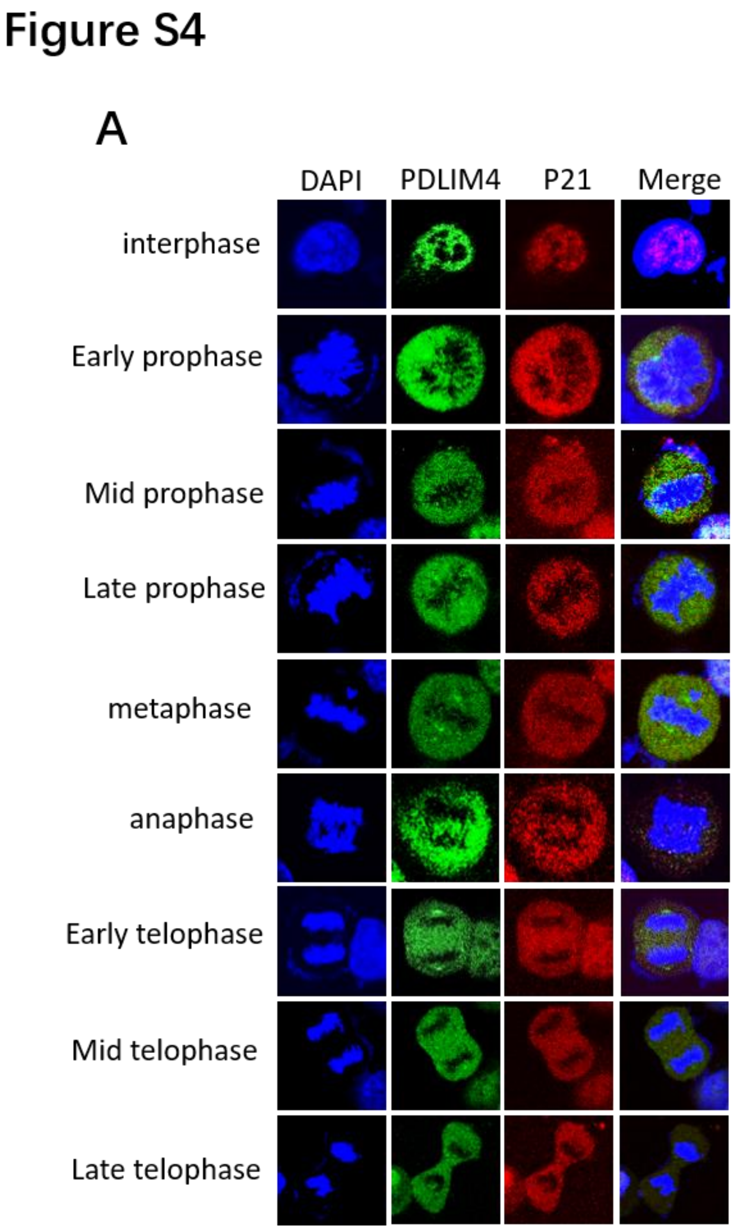

Figure S5. The regulation of BRD4/PDLIM4/p21 axis in LUAD cells. A The relative expression levels of p21 and flag-PDLIM4 were analyzed by western blotting following stable overexpression of PDLIM4. Data represented mean ± SEM. *p < 0.05. **B-D** Representative plots of immunohistochemistry (IHC) staining for PDLIM4 and p21 in mice xenograft tumor derived from vector or OV-PDLIM4 of A549 cells. Scale bar, 200μm (magnification, ×40). **E** The relative expression levels of p21 and flag-PDLIM4 were analyzed by western blotting in mice xenograft tumor derived from vector or OV-PDLIM4 of A549 cells. **F** Real-time PCR assay analysis of the expression of p21 expression in vector or OV-PDLIM4 of A549 cells. Data represented mean ± SEM. **p < 0.01. **G** Real-time PCR assay analysis of the expression of p21 expression in mice xenograft tumor. Data represented mean ± SEM. *p < 0.05. **H** The relative expression levels of p21 and PDLIM4 were analyzed by western blotting after stable knockdown of PDLIM4. Data represented mean ± SEM. *p < 0.05. **I** Real-time PCR assay analysis of the expression of p21 expression in A549 after stable knockdown of PDLIM4. Data represented mean ± SEM. *p < 0.05. **J** Western blot analysis of the expression of PDLIM4 and p21 in A549 cells treated with JQ1 (1μM) for 24h. **K** The mRNA level of PLDLIM4 and p21 were checked by real-time PCR in A549 cells with JQ1 (1μM) treatment for 6h. Data represented mean ± SEM. **p < 0.01. **L** Immunoblotting demonstrated the expression of PDLIM4 and p21 following stable overexpression of BRD4 in A549 cells. **M** The mRNA level of PLDLIM4 and p21 were checked by real-time PCR following stable overexpression of BRD4 in A549 cells. Data represented mean ± SEM. *p < 0.05. **N-P** The expression of PDLIM4 and p21 were detected by real-time PCR and immunoblotting after stable knockdown of BRD4 in A549 cells. Data represented mean ± SEM. *p < 0.05. **Q-S** The expression of PDLIM4 and p21 were measured in control (GFP-vector) and overexpression of BD1 of A549 cells by real-time PCR and immunoblotting. Data represented mean ± SEM. *p < 0.05. **T-U** After PDLIM4 knockdown in control and stable overexpression of BRD4 cells, the expression of p21 were examined by real-time PCR and immunoblotting in A549 cells. Data represented mean ± SEM. **p <

0.01. **V-W** Cells overexpressing wild-type PDLIM4 (WT) were reintroduced to assess changes in p21 protein and RNA levels. **X** Western blot analysis was performed to determine the protein levels of p21 in H1299 cells treated with 100μg/mL CHX (cycloheximide) for 3 to 12 hrs. **Y** Maintained control or knockdown PDLIM4 of A549 cells in media containing Actinomycin D (Act D, 10μg/mL, Selleck), quantitative PCR was utilized to examine intracellular mRNA level of p21 at 0, 3, 6, and 9 h. Data represented mean ± SEM. *p < 0.05. All the experiments were repeated at least three times.

Figure S5

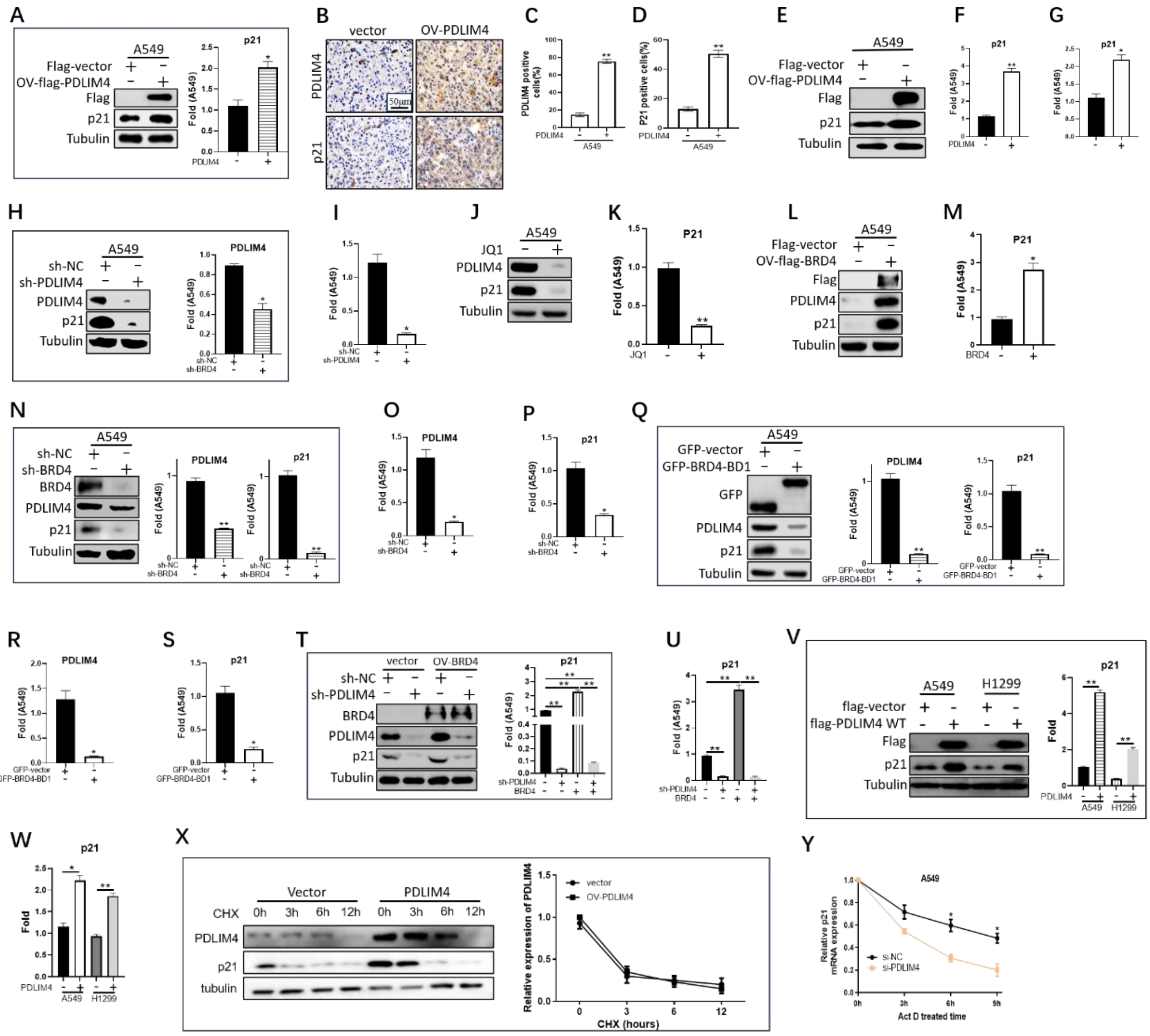

Figure S6 BRD4-PDLIM4-p21 pathway sensitizes chemotherapy. A-B The protein and mRNA level of BRD4, PDLIM4 and p21 were detected in A549 cells with 0.5μM DOX treatment for 2hrs, Data represented mean ± SEM. ***$p < 0.001$, **$p < 0.01$. **C-D** Western blot and real-time PCR analysis of the expression of PDLIM4 and p21 in A549 cells with JQ1 or DOX treatment. Data represented mean ± SEM. **$p < 0.01$, *$p < 0.05$. **E-F** Western blot and real-time PCR analysis of the expression of BRD4, PDLIM4 and p21 in control A549 cells or BRD4 knockdown cells following with DOX treatment. Data represented mean ± SEM. ***$p < 0.001$, **$p < 0.01$, *$p < 0.05$. **G-H** Western blot and real-time PCR analysis of the expression of PDLIM4 and p21 in control A549 cells or PDLIM4 knockdown cells following with DOX treatment. Data represented mean ± SEM. **$p < 0.01$. **I** A549 stably overexpressing PDLIM4 cells and control cells were treated with DOX for 48h followed by CCK8 assays. Data represented mean ± SEM. **$p < 0.01$. **J** Xenograft tumors were generated by injecting A549 vector or A549 overexpressing PDLIM4 cells into dorsal flanking sites of nude mice. Two weeks later, mice were treated with DOX (3mg/kg, three times per week) for 2 weeks. **K** Quantitative results for Figure 6J. Data represented mean ± SEM. **$p < 0.01$, *$p < 0.05$. L Western blot was used to detected the expression of p21. M Quantitation data for figure 6L. **$p < 0.01$. **N-O** Real-time PCR and IHC staining were used to examine the expression of p21. 100×plots with a scale bar of 200μm. **P-Q** Xenograft tumors were generated by injecting H1299 cells or A549 cells into dorsal flanking sites of nude mice. Two weeks later, mice were treated with DOX (3mg/kg, three times per week) for 2 weeks. Representative plots of IHC staining for BRD4, PDLIM4 and p21 (left). Quantitation of the results were shown on the right. Data are presented as the mean ± SEM. ***$p < 0.001$, **$p < 0.01$, *$p < 0.05$. All the experiments were repeated at least three times.

Figure S6

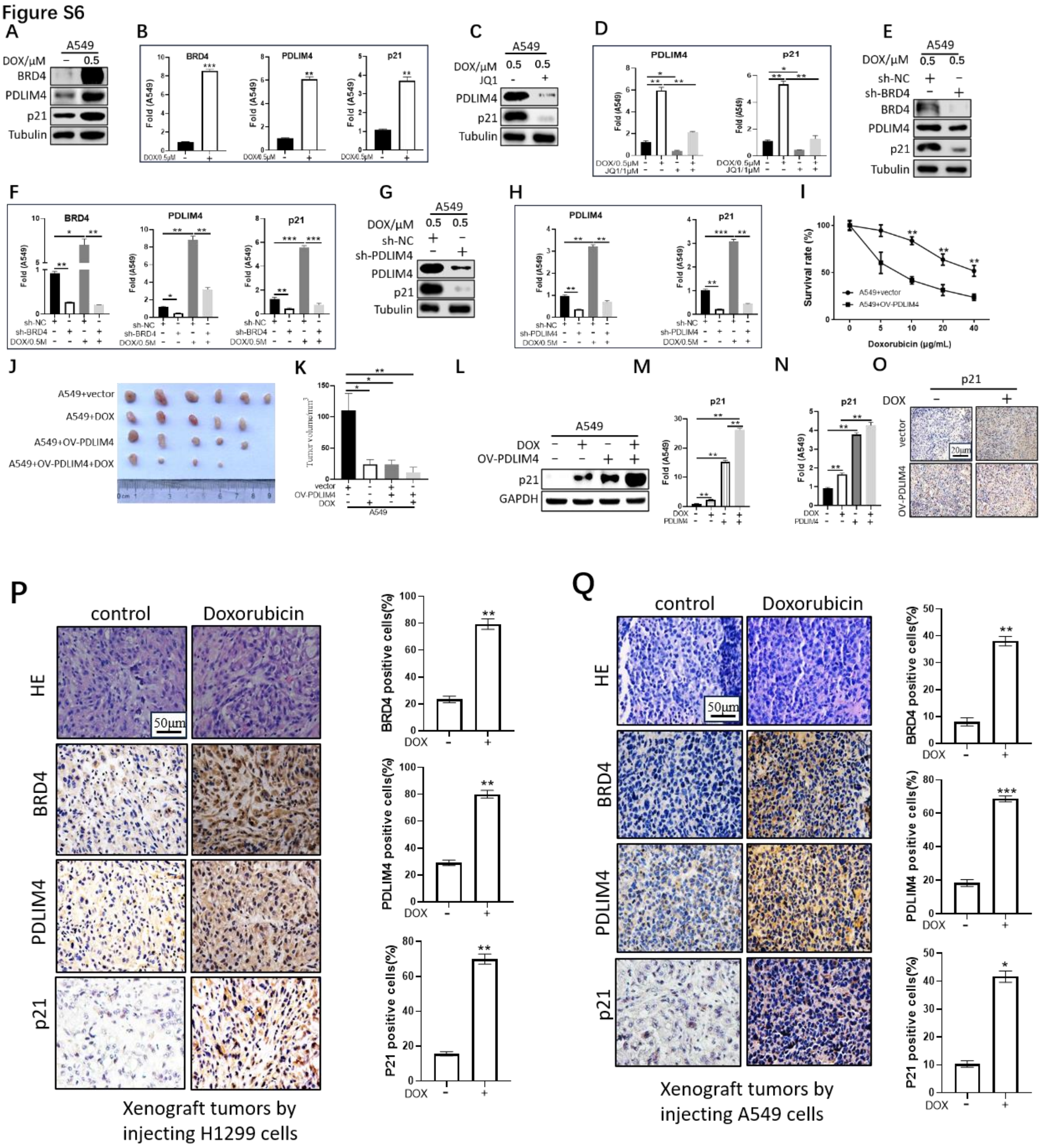

A
B
C
D
E
F
G
H
I
J
K
L
M
N
O
P
Q
A549
DOX/μM
BRD4
PDLIM4
p21
Tubulin
JQ1
sh-NC
sh-BRD4
sh-PDLIM4
GAPDH
Fold (A549)
Survival rate (%)
Doxorubicin (μg/mL)
A549+vector
A549+OV-PDLIM4
A549+DOX
A549+OV-PDLIM4+DOX
OV-PDLIM4
DOX
vector
control
Doxorubicin
HE
50μm
20μm
BRD4 positive cells(%)
PDLIM4 positive cells(%)
P21 positive cells(%)
Xenograft tumors by injecting H1299 cells
Xenograft tumors by injecting A549 cells

Figure S7 Rescue experiment. A-B Western blot and qPCR were performed to analyze p21 protein and RNA levels after PDLIM4 overexpression in H1299 (p53-null) and A549 (WT-p53) cells. **C-D** H1299 and A549 stably overexpressing PDLIM4 S116A cells or control cells or overexpression PDLIM4 WT were treated with DOX for 48h followed by CCK8 assays. Data represented mean ± SEM. **p < 0.01, *p < 0.05.

Figure S7

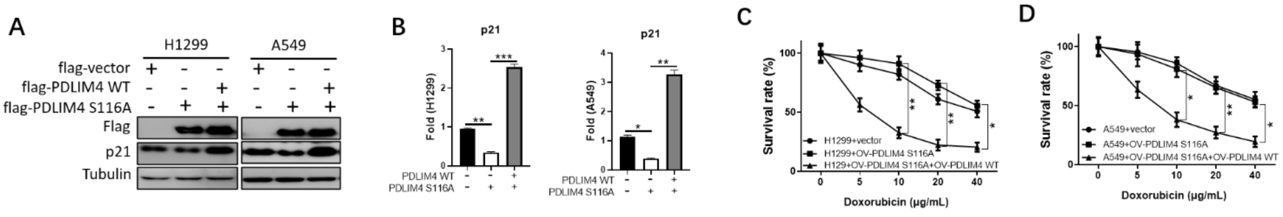

Supplemental Tables 1-2

Table S1: **All shRNA sequences of primers used in this paper.**

BRD2-shRNA: CCGGGCTGCTGATGTACGGCTTATGCTCGAGCATAAGCCGTACATCAGCAGCTTTTTT
BRD3-shRNA: CCGGGGGAGATGCTATCCAAGAAGCCTCGAGGCTTCTTGGATAGCATCTCCCTTTTTT
BRD4-shRNA: CCGGCAGAGTGATCTATTGTCAATACTCGAGTATTGACAATAGATCACTCTGTTTTTT
PDLIM4-shRNA-1: CCGGGCACACAGGATCCACATCGATCTCGAGATCGATGTGGATCCTGTGTGCTTTTTT
PDLIM4-shRNA-2: CCGGGAACCTCAAGCAGCGTGGTTACTCGAGTAACCACGCTGCTTGAGGTTCTTTTTT

Table S2: **Sequences of homo primers used for RT-PCR**.

Gene name	Primer sequences	
	Forward	Reverse
GAPDH	CTGGGCTACACTGAGCACC	AAGTGGTCGTTGAGGGCAATG
P21	TGTCCGTCAGAACCCATGC	AAAGTCGAAGTTCCATCGCTC
PDLIM4	CATCGTGGGCACCATCGTCAAG	GCCGCTCGTCCAGAAAGAAGTAA
BRD2	GAGGTGTCCAATCCCAAAAAGC	ATGCGAACTGATGTTTCCACA
BRD3	TCAAATTGAACCTGCCGGATT	TGCATACATTCGCTTGCACTC
BRD4	GAGCTACCCACAGAAGAAACC	GAGTCGATGCTTGAGTTGTGTT

BRD4-Mediated Transcriptional Activation of PDLIM4 Enhances p21 Stability and Chemosensitivity in Lung Adenocarcinoma Independent of p53

original data

figure1

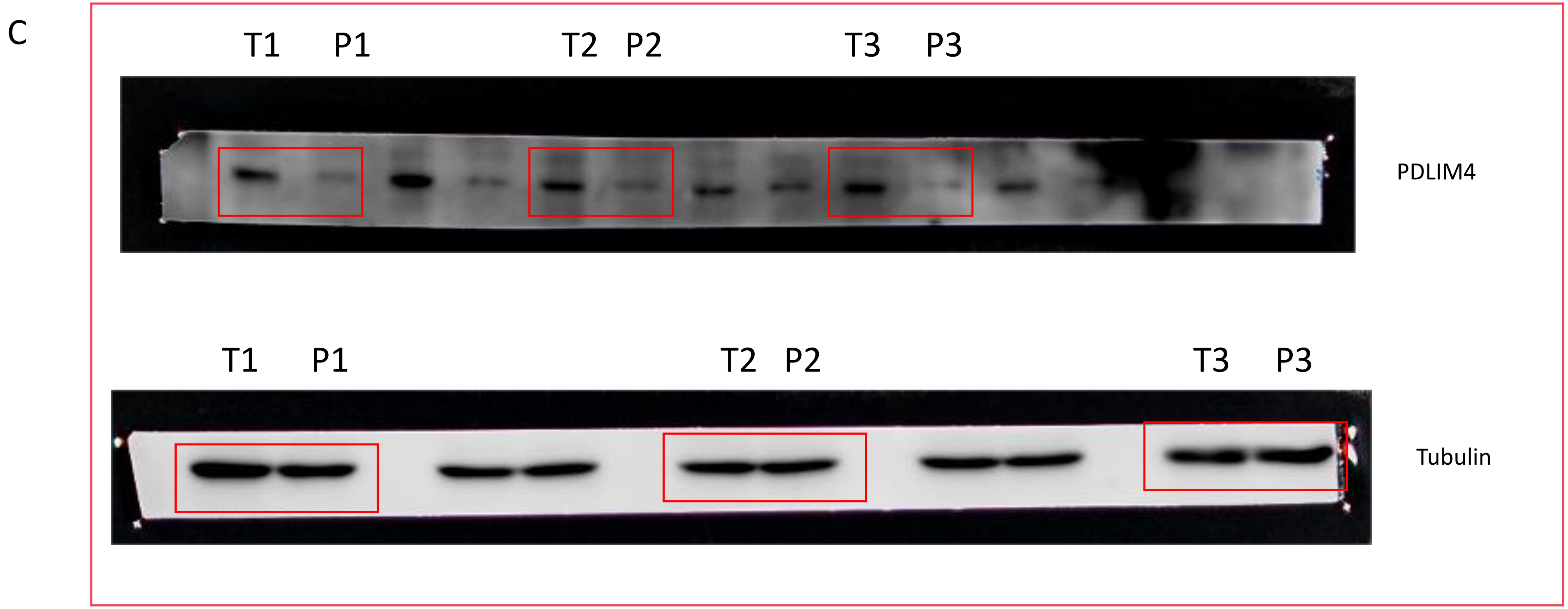

figure3

B

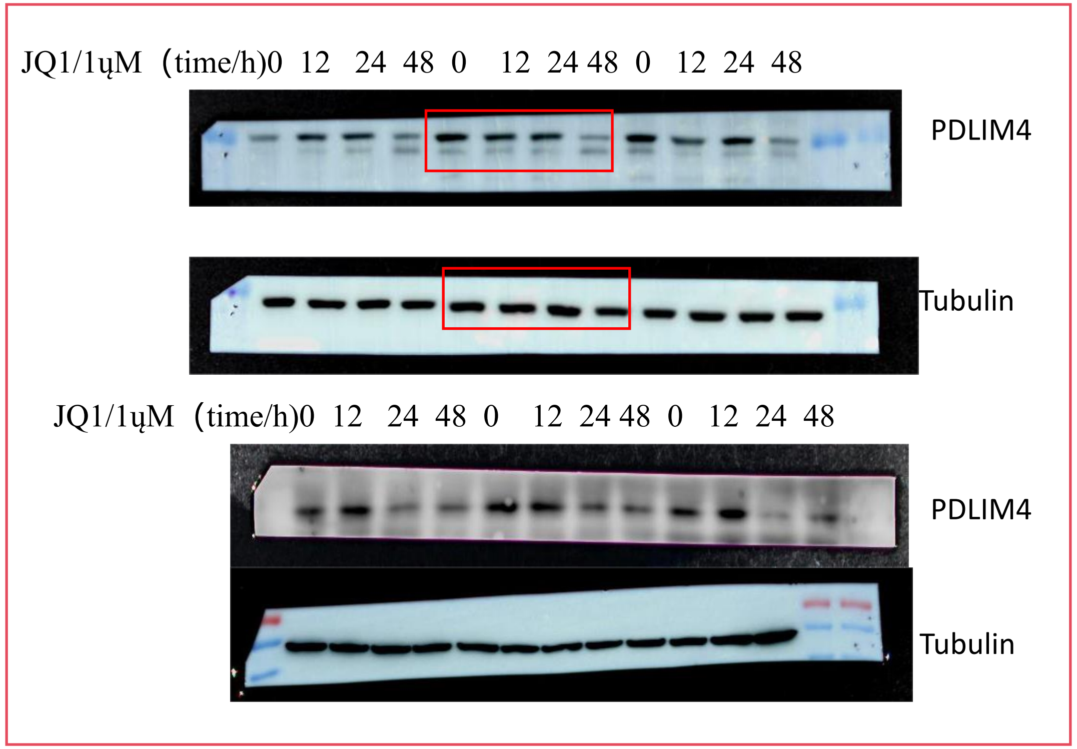
JQ1/1ųM (time/h)0 12 24 48 0 12 24 48 0 12 24 48
PDLIM4
Tubulin
JQ1/1ųM (time/h)0 12 24 48 0 12 24 48 0 12 24 48
PDLIM4
Tubulin

D

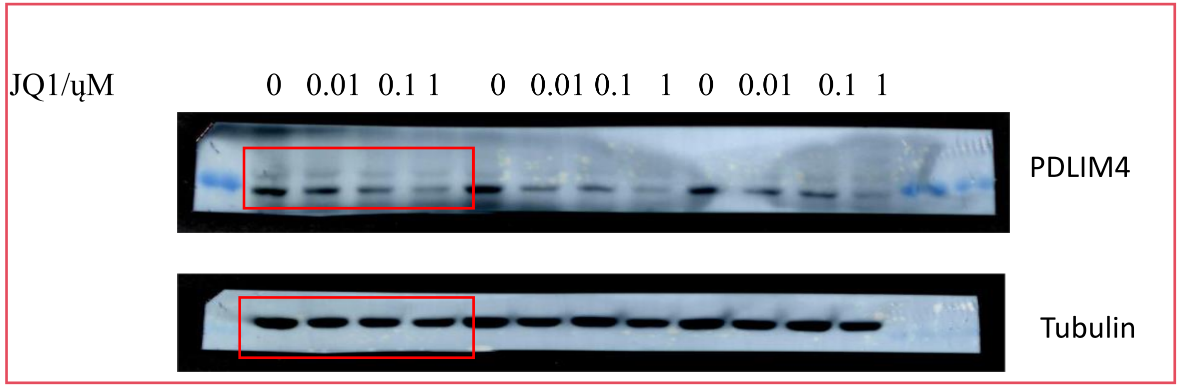
JQ1/ųM 0 0.01 0.1 1 0 0.01 0.1 1 0 0.01 0.1 1
PDLIM4
Tubulin

G

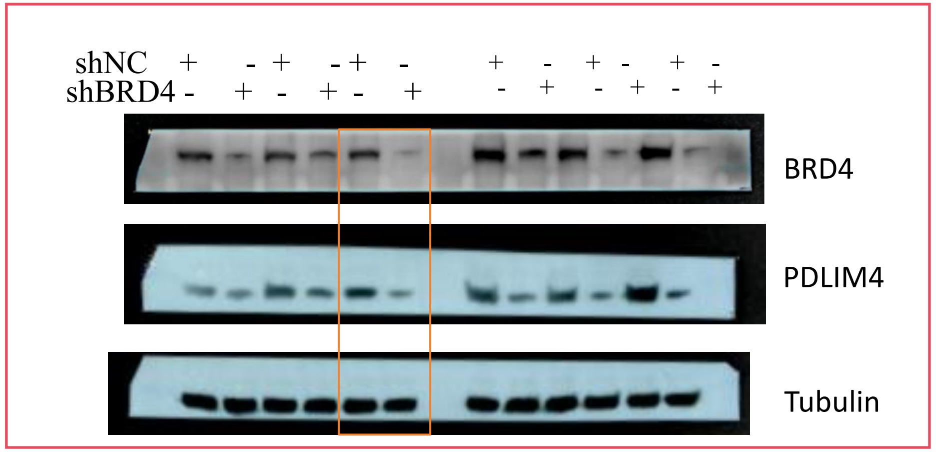
shNC + - + - + - + - + - + -
shBRD4 - + - + - + - + - + - +
BRD4
PDLIM4
Tubulin

L

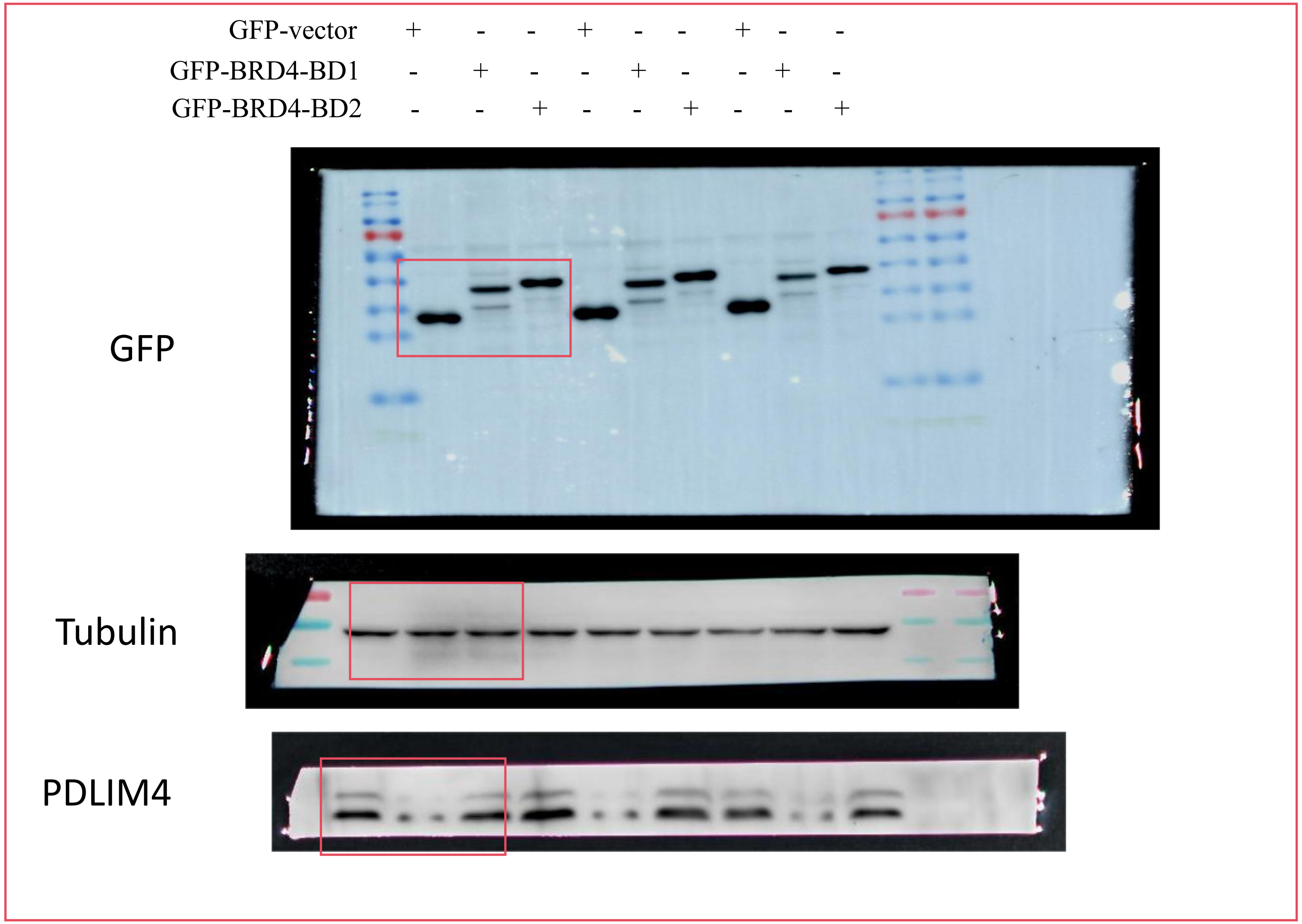

figure 4

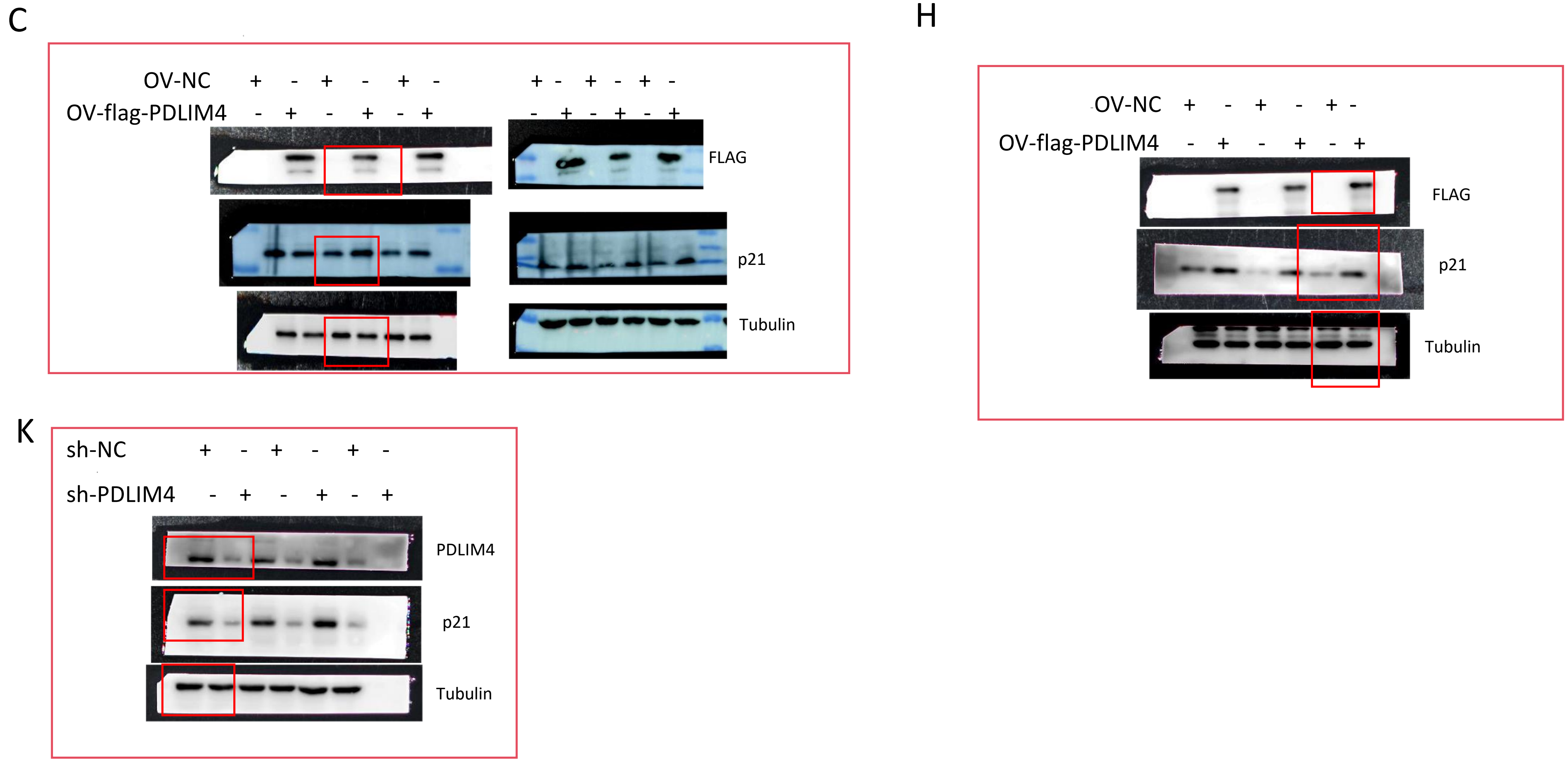
C
OV-NC + - + - + - + - + - + -
OV-flag-PDLIM4 - + - + - + - + - + - +
FLAG
p21
Tubulin
H
OV-NC + - + - + -
OV-flag-PDLIM4 - + - + - +
FLAG
p21
Tubulin
K
sh-NC + - + - + -
sh-PDLIM4 - + - + - +
PDLIM4
p21
Tubulin

figure 5

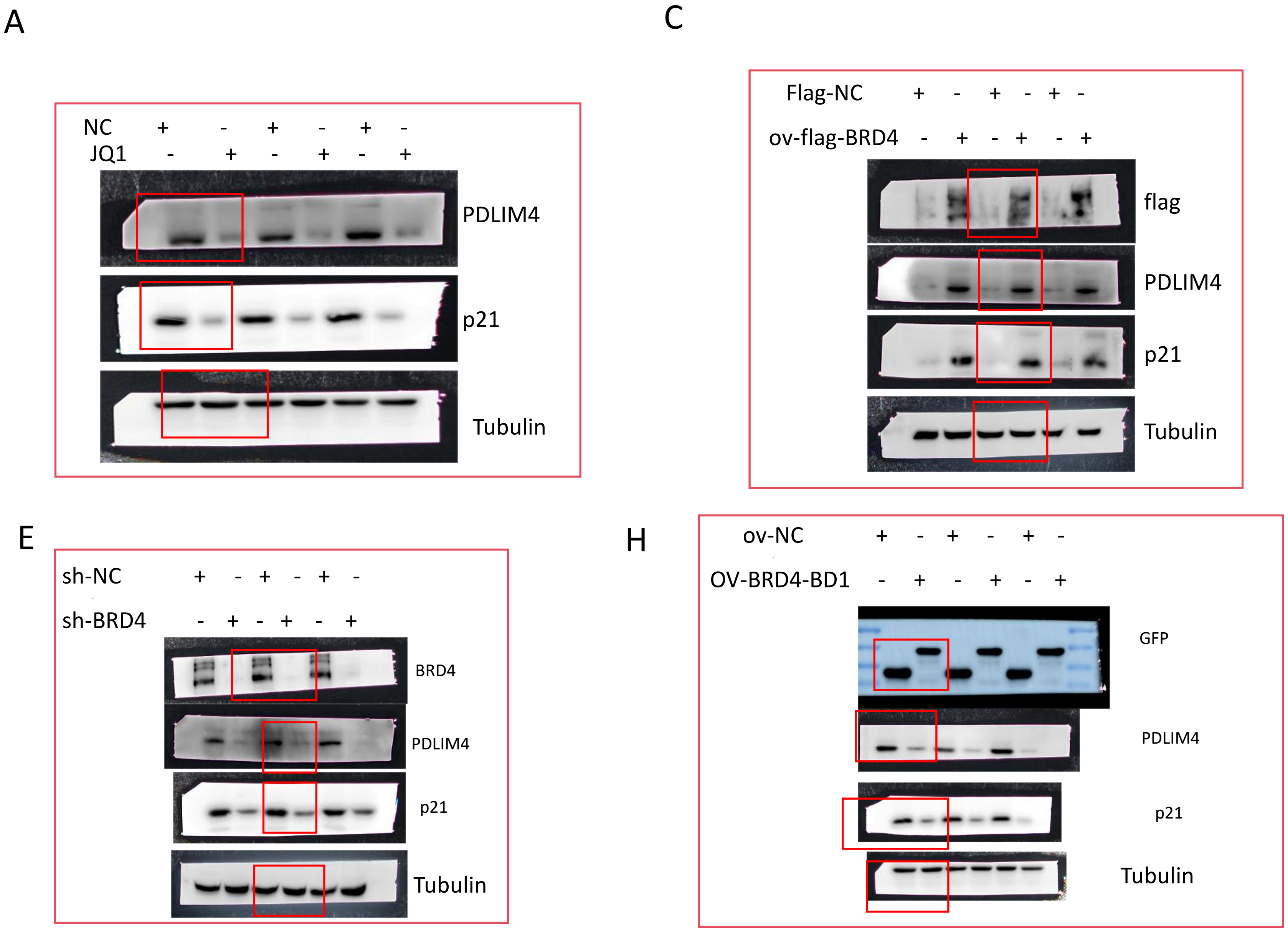
A
NC + - + - + -
JQ1 - + - + - +
PDLIM4
p21
Tubulin
C
Flag-NC + - + - + -
ov-flag-BRD4 - + - + - +
flag
PDLIM4
p21
Tubulin
E
sh-NC + - + - + -
sh-BRD4 - + - + - +
BRD4
PDLIM4
p21
Tubulin
H
ov-NC + - + - + -
OV-BRD4-BD1 - + - + - +
GFP
PDLIM4
p21
Tubulin

figure 5

K

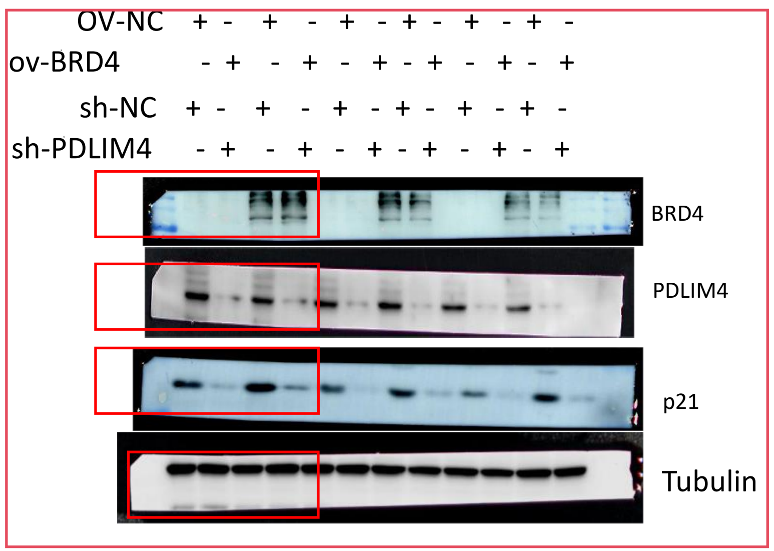

figure 6

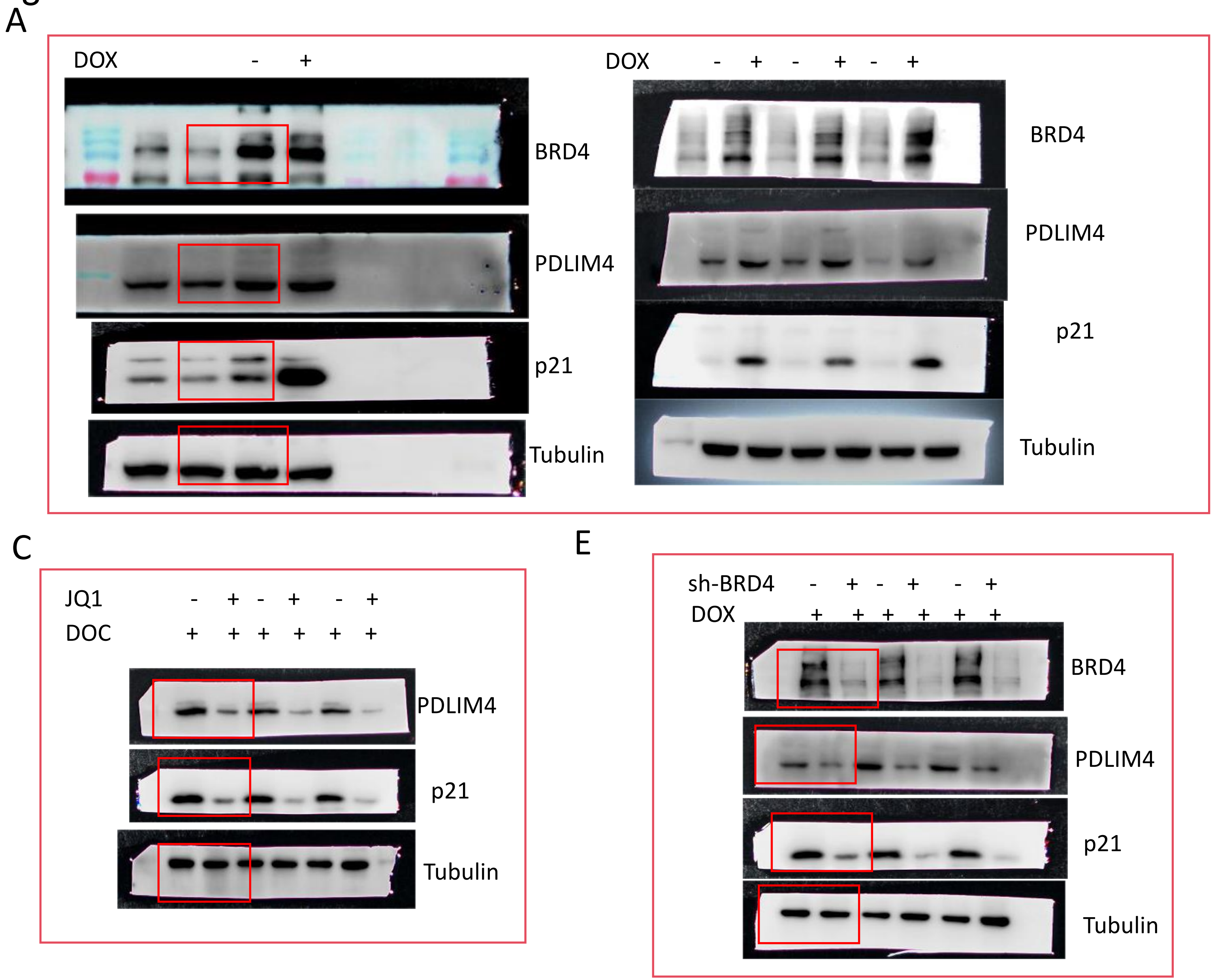
A
DOX - +
BRD4
PDLIM4
p21
Tubulin
DOX - + - + - +
BRD4
PDLIM4
p21
Tubulin
C
JQ1 - + - + - +
DOC + + + + + +
PDLIM4
p21
Tubulin
E
sh-BRD4 - + - + - +
DOX + + + + + +
BRD4
PDLIM4
p21
Tubulin

G

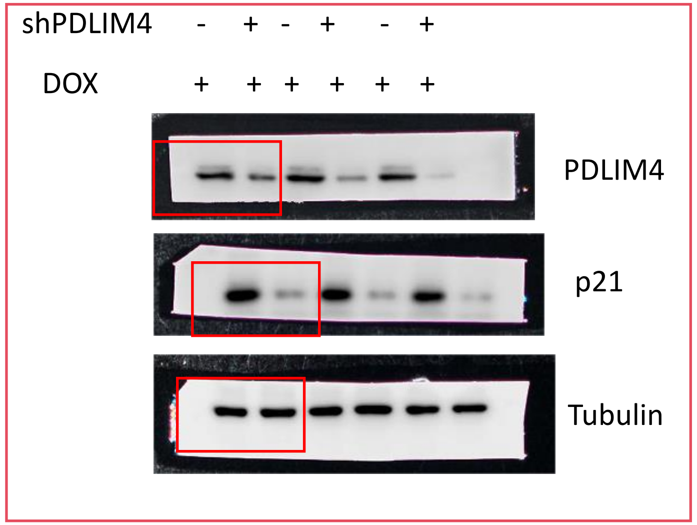

L

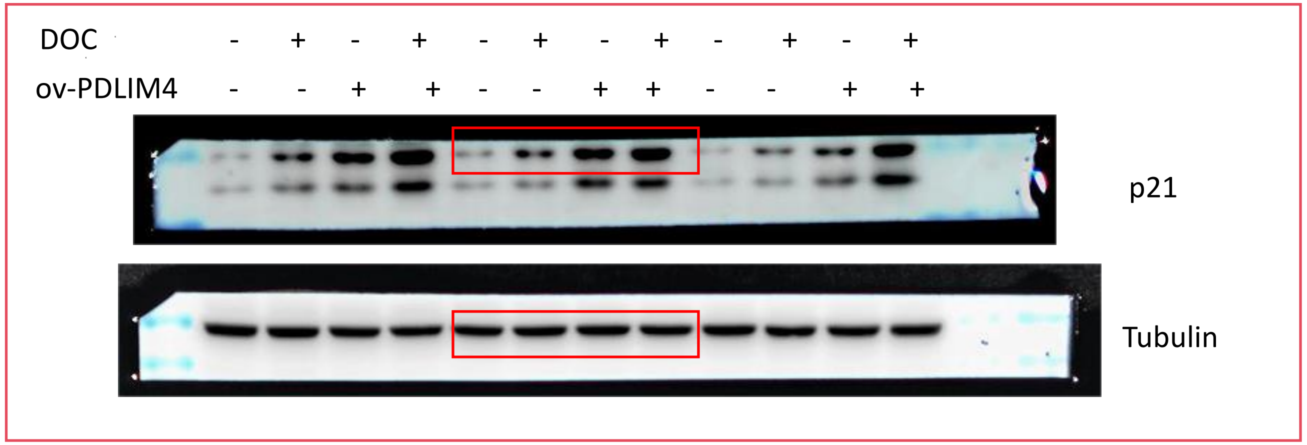

figure 7

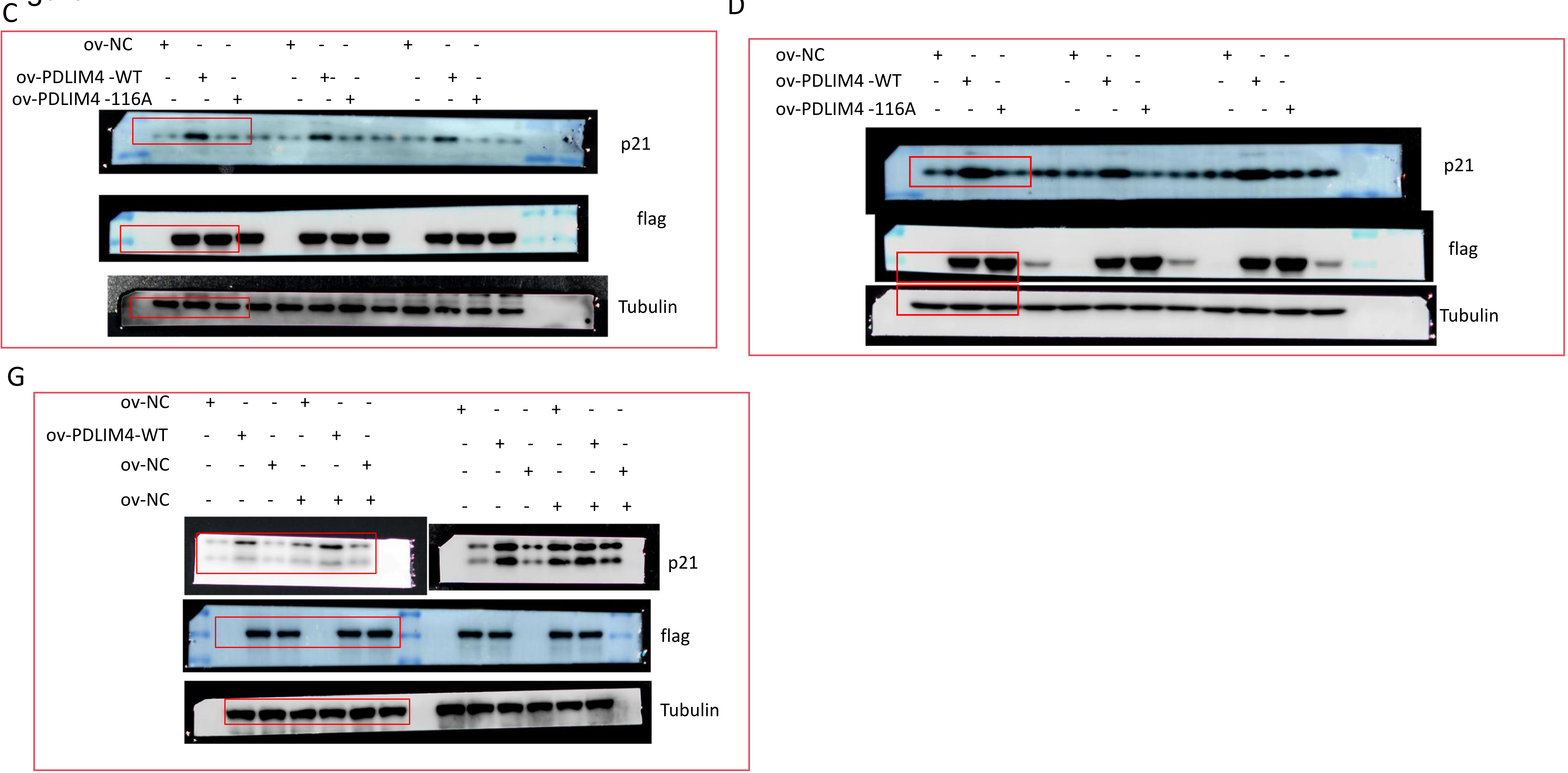
C
ov-NC + - - + - - + - -
ov-PDLIM4 -WT - + - - +- - - + -
ov-PDLIM4 -116A - - + - - + - - +
p21
flag
Tubulin
D
ov-NC + - - + - - + - -
ov-PDLIM4 -WT - + - - + - - + -
ov-PDLIM4 -116A - - + - - + - - +
p21
flag
Tubulin
G
ov-NC + - - + - - + - - + - -
ov-PDLIM4-WT - + - - + - - + - - + -
ov-NC - - + - - + - - + - - +
ov-NC - - - + + + - - - + + +
p21
flag
Tubulin

Supplemental data

Figure 1

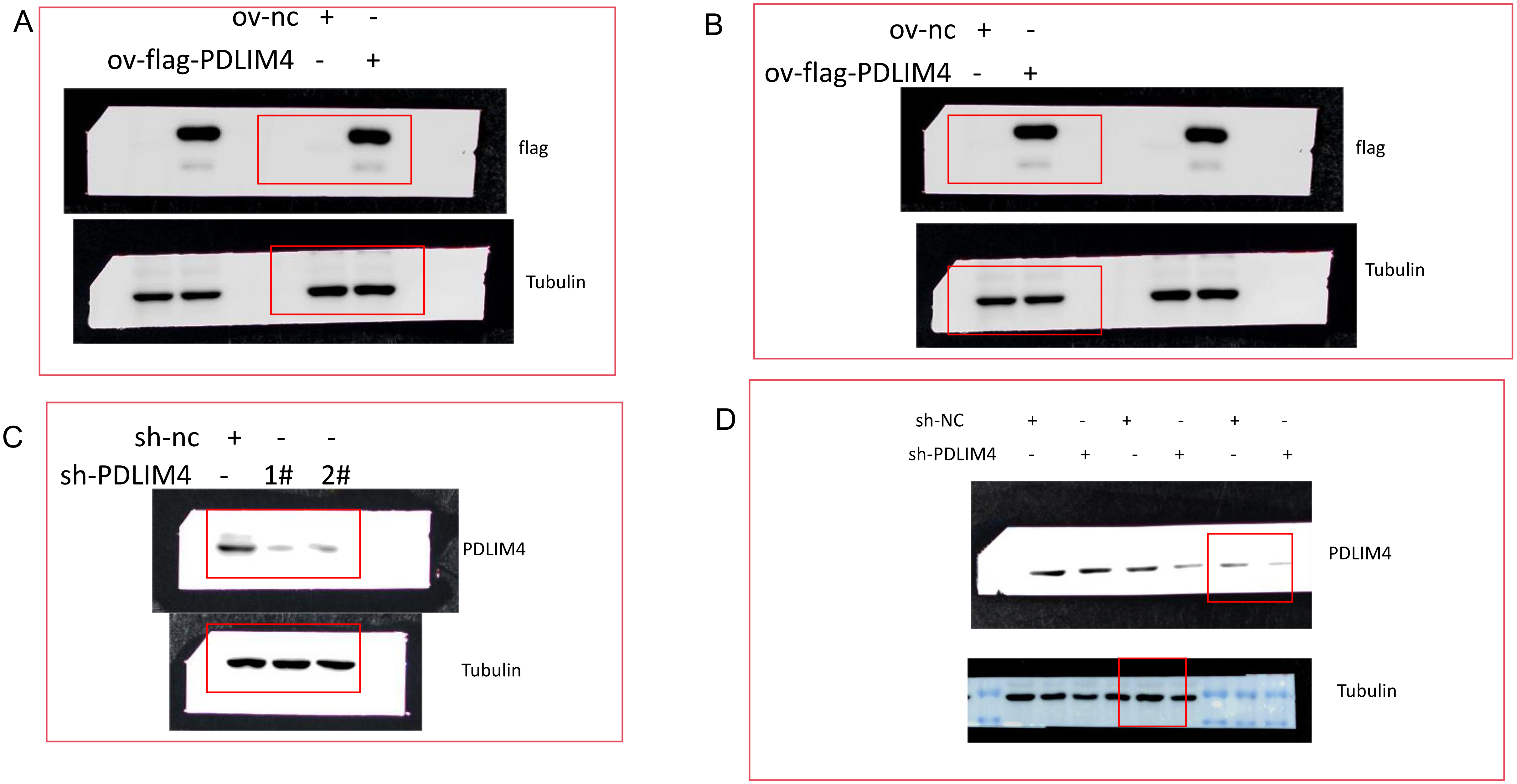

E

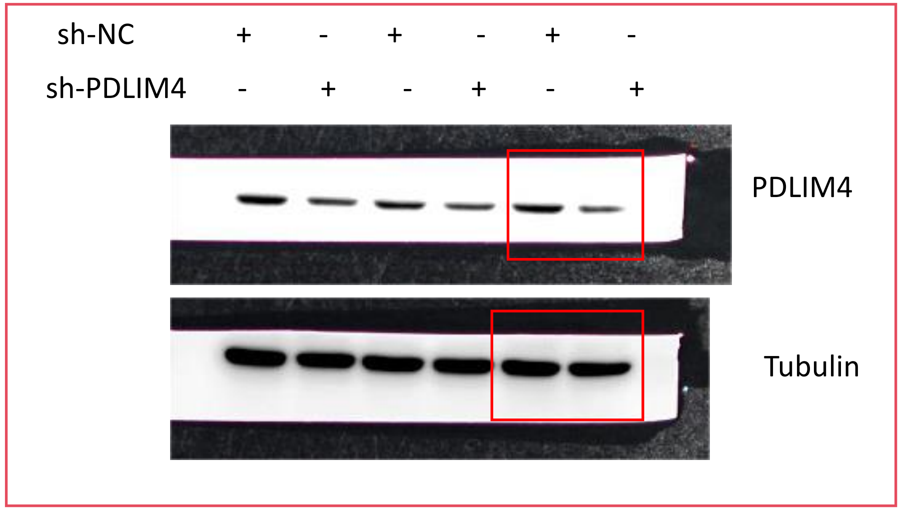

Supplemental data
figure 3

A

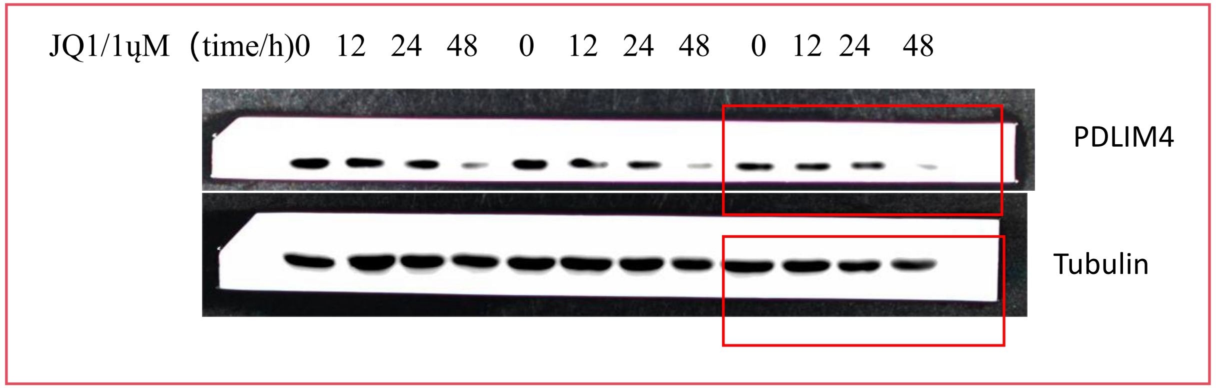

C

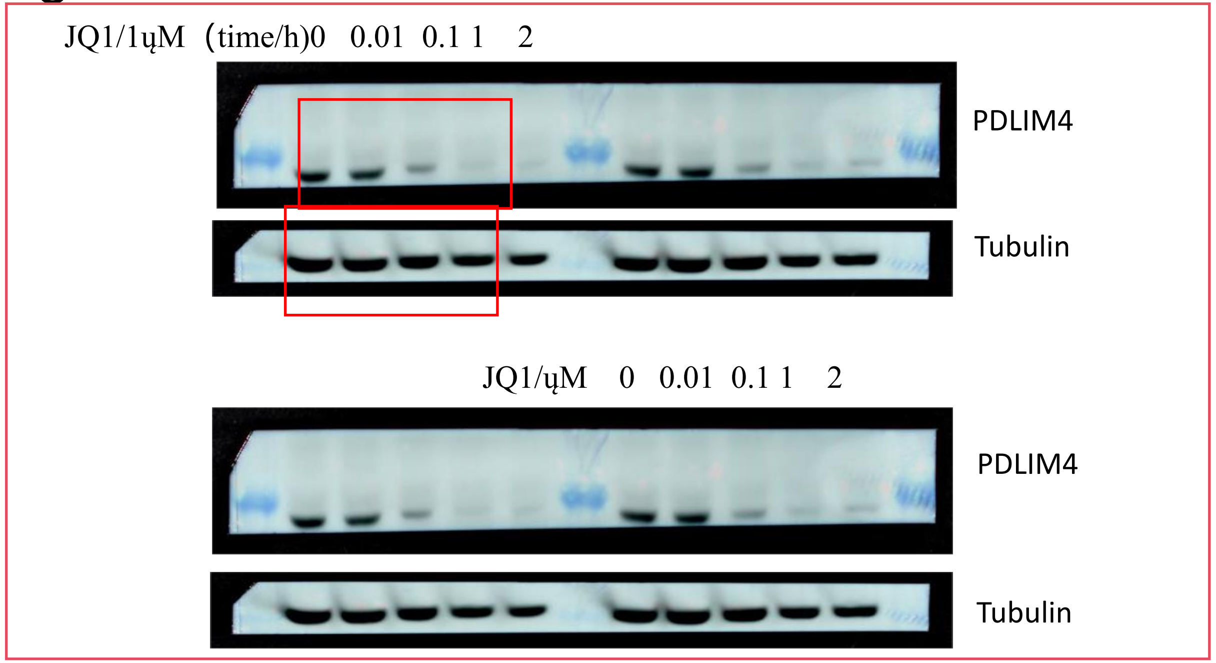

E

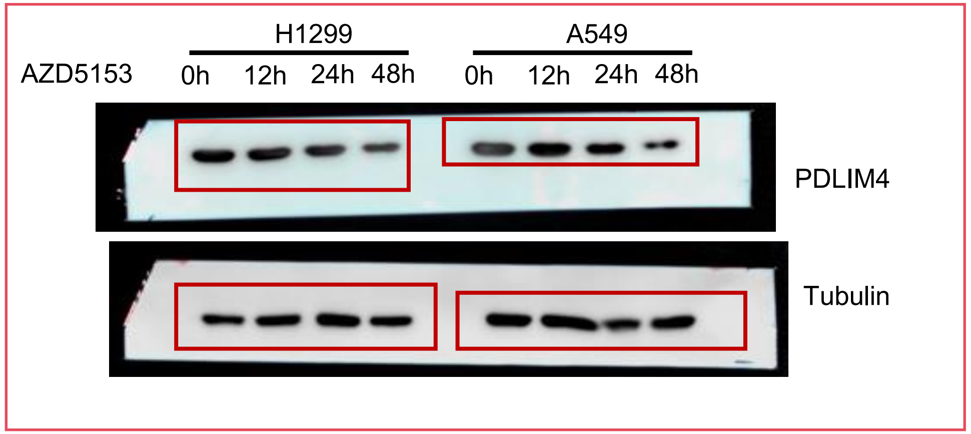

F

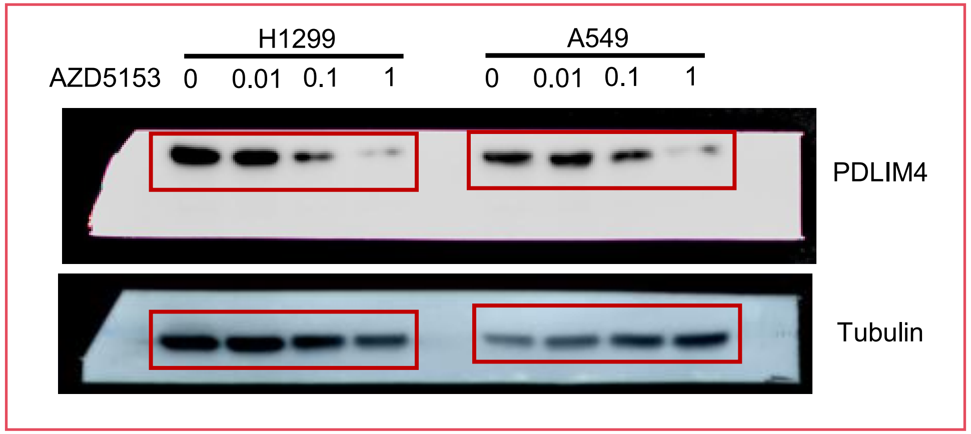

M

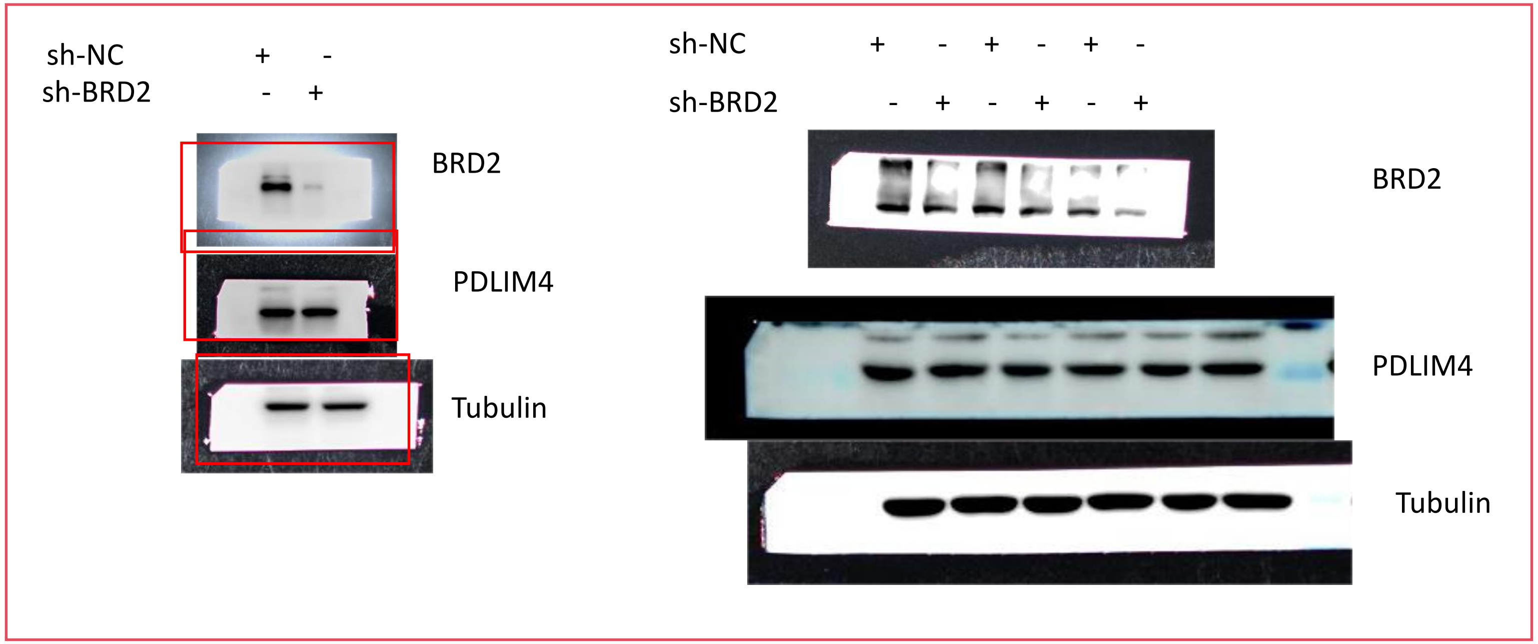
sh-NC + -
sh-BRD2 - +
BRD2
PDLIM4
Tubulin
sh-NC + - + - + -
sh-BRD2 - + - + - +
BRD2
PDLIM4
Tubulin

N

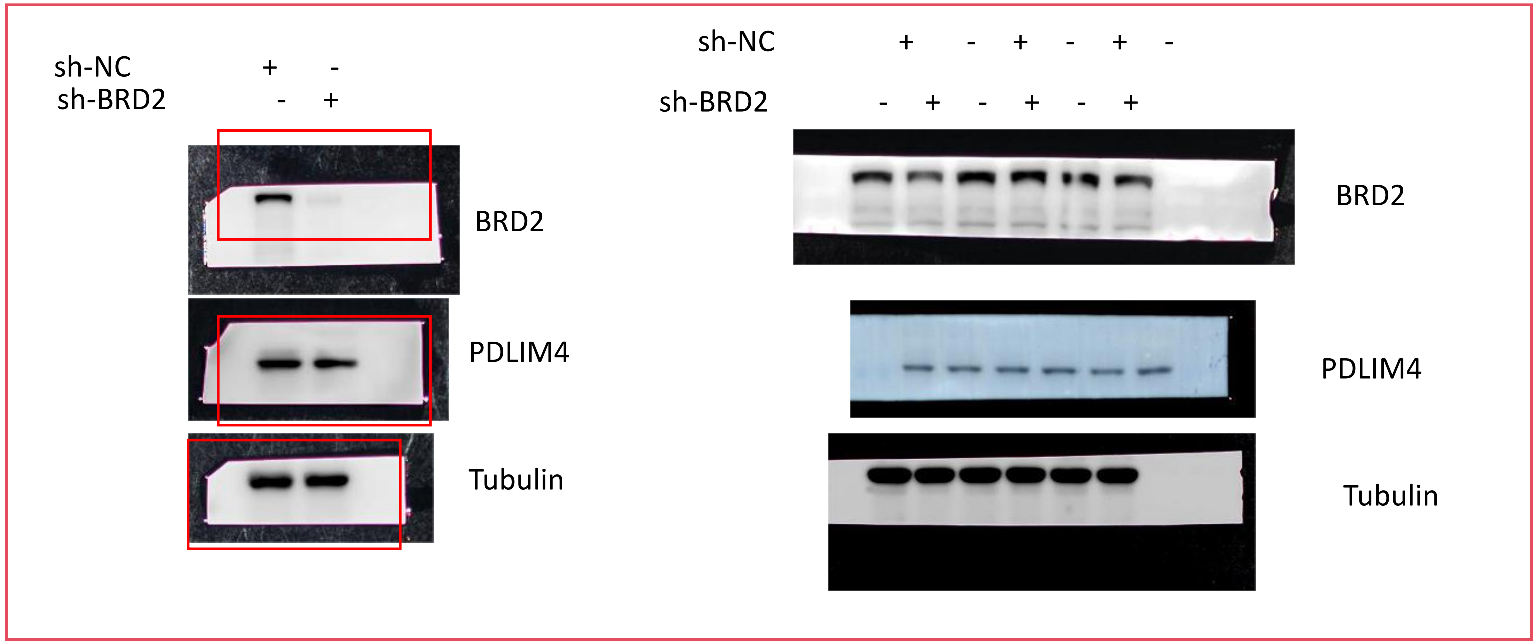
sh-NC + -
sh-BRD2 - +
BRD2
PDLIM4
Tubulin
sh-NC + - + - + -
sh-BRD2 - + - + - +
BRD2
PDLIM4
Tubulin

O

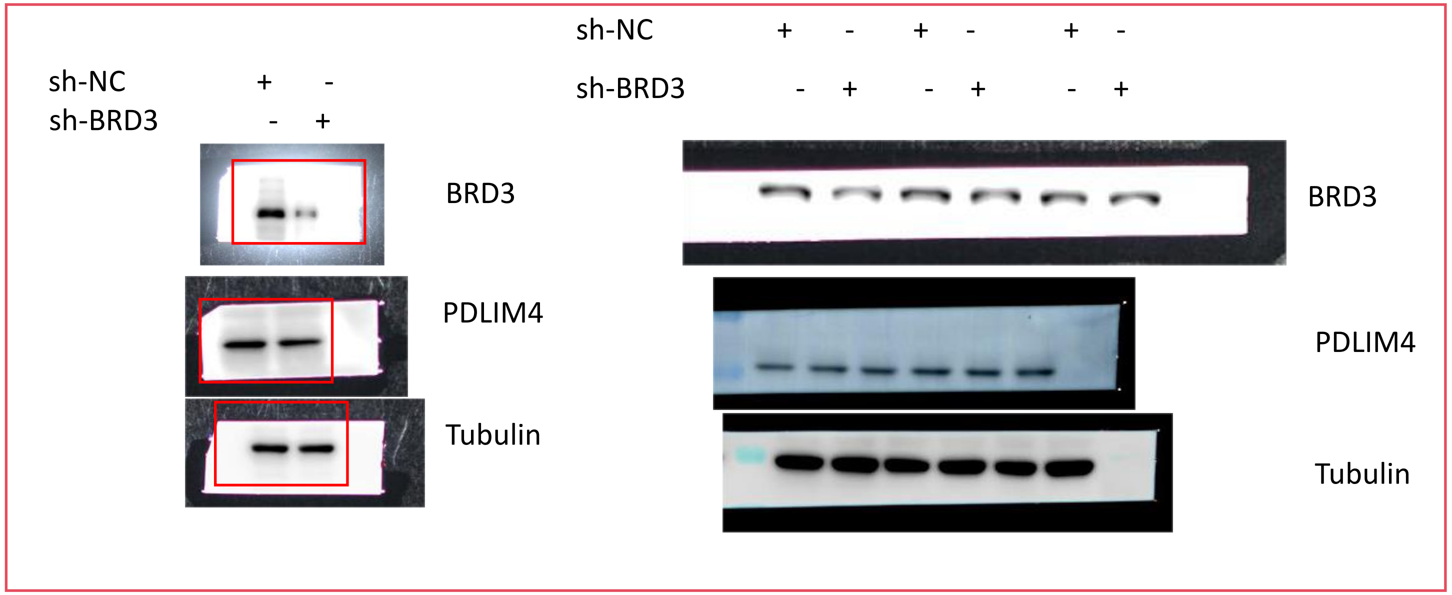
sh-NC + -
sh-BRD3 - +
BRD3
PDLIM4
Tubulin
sh-NC + - + - + -
sh-BRD3 - + - + - +
BRD3
PDLIM4
Tubulin

P

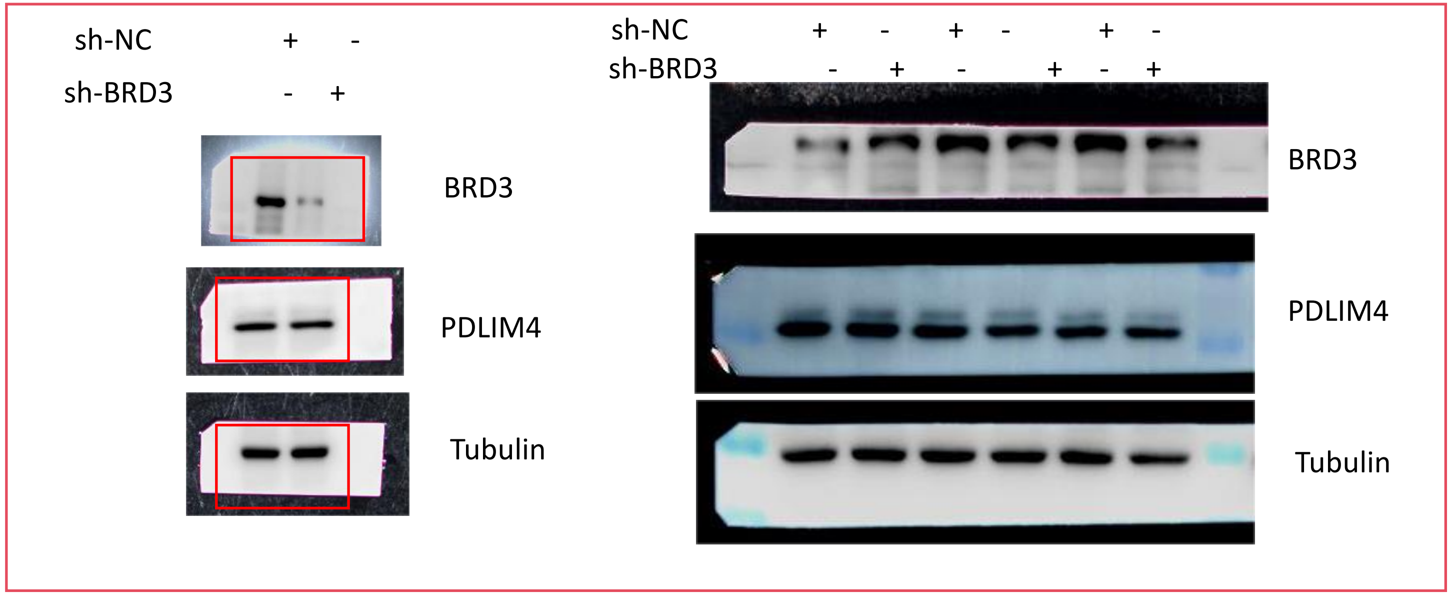
sh-NC + -
sh-BRD3 - +
BRD3
PDLIM4
Tubulin
sh-NC + - + - + -
sh-BRD3 - + - + - +
BRD3
PDLIM4
Tubulin

R

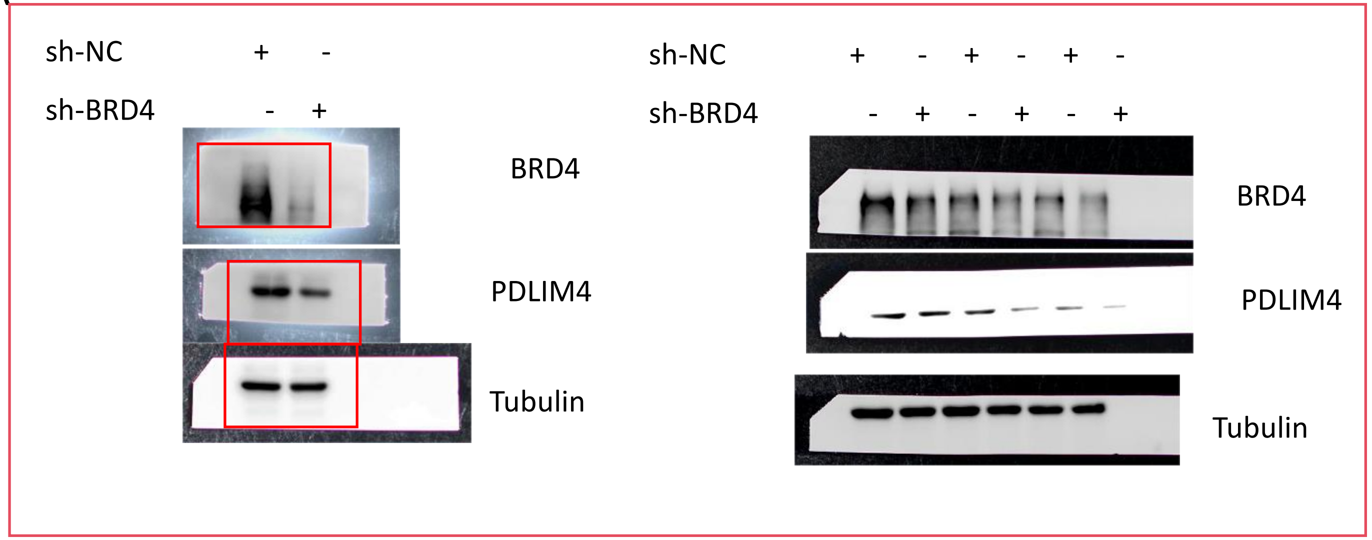
sh-NC + -
sh-BRD4 - +
BRD4
PDLIM4
Tubulin
sh-NC + - + - + -
sh-BRD4 - + - + - +
BRD4
PDLIM4
Tubulin

T

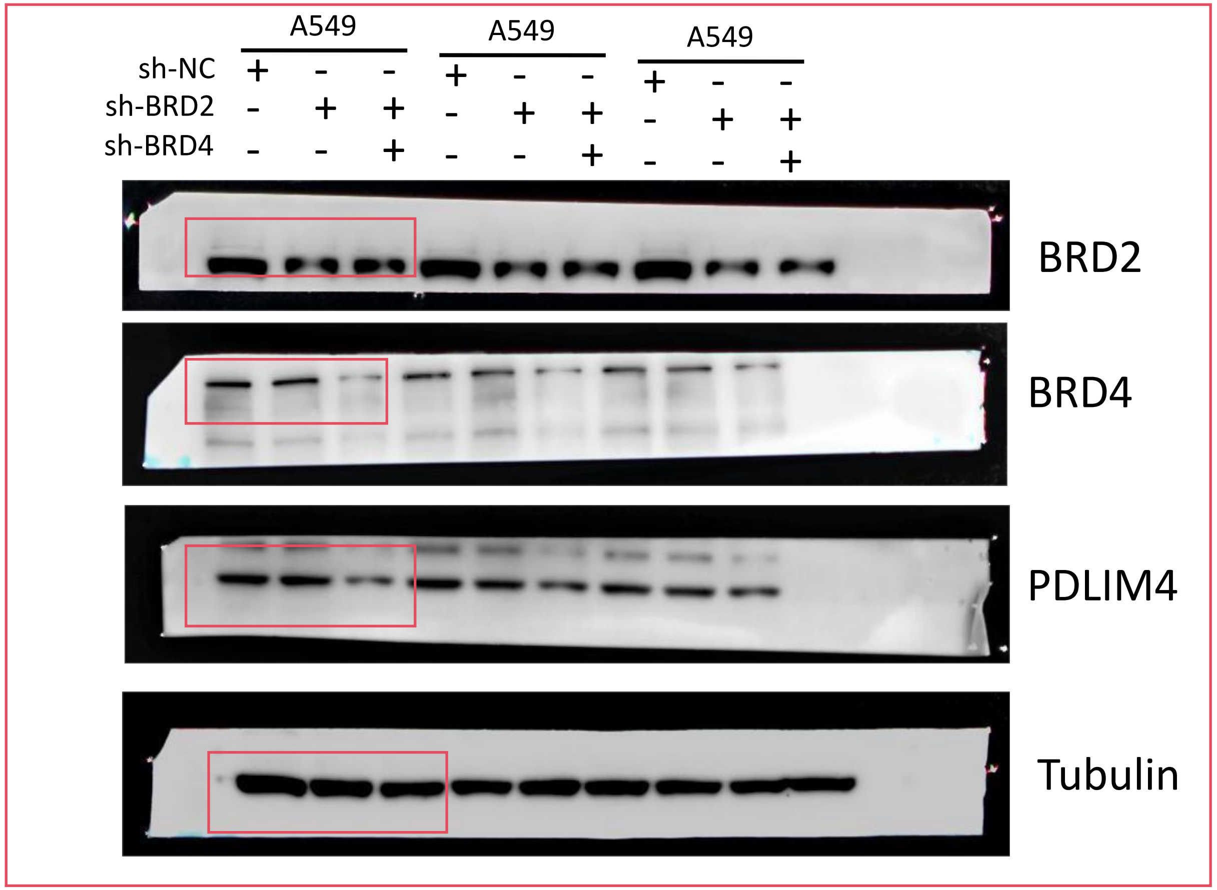
A549
A549
A549
sh-NC + - - + - - + - -
sh-BRD2 - + + - + + - + +
sh-BRD4 - - + - - + - - +
BRD2
BRD4
PDLIM4
Tubulin

U

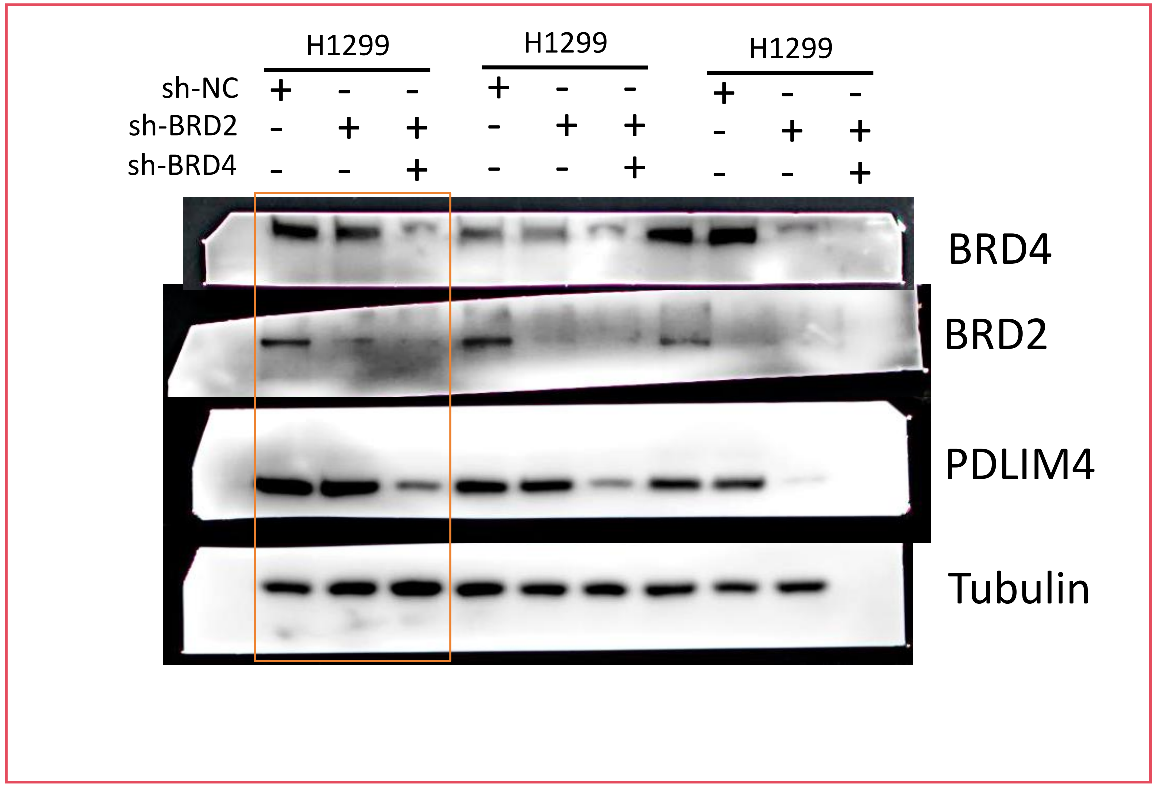
H1299
H1299
H1299
sh-NC + - - + - - + - -
sh-BRD2 - + + - + + - + +
sh-BRD4 - - + - - + - - +
BRD4
BRD2
PDLIM4
Tubulin

V

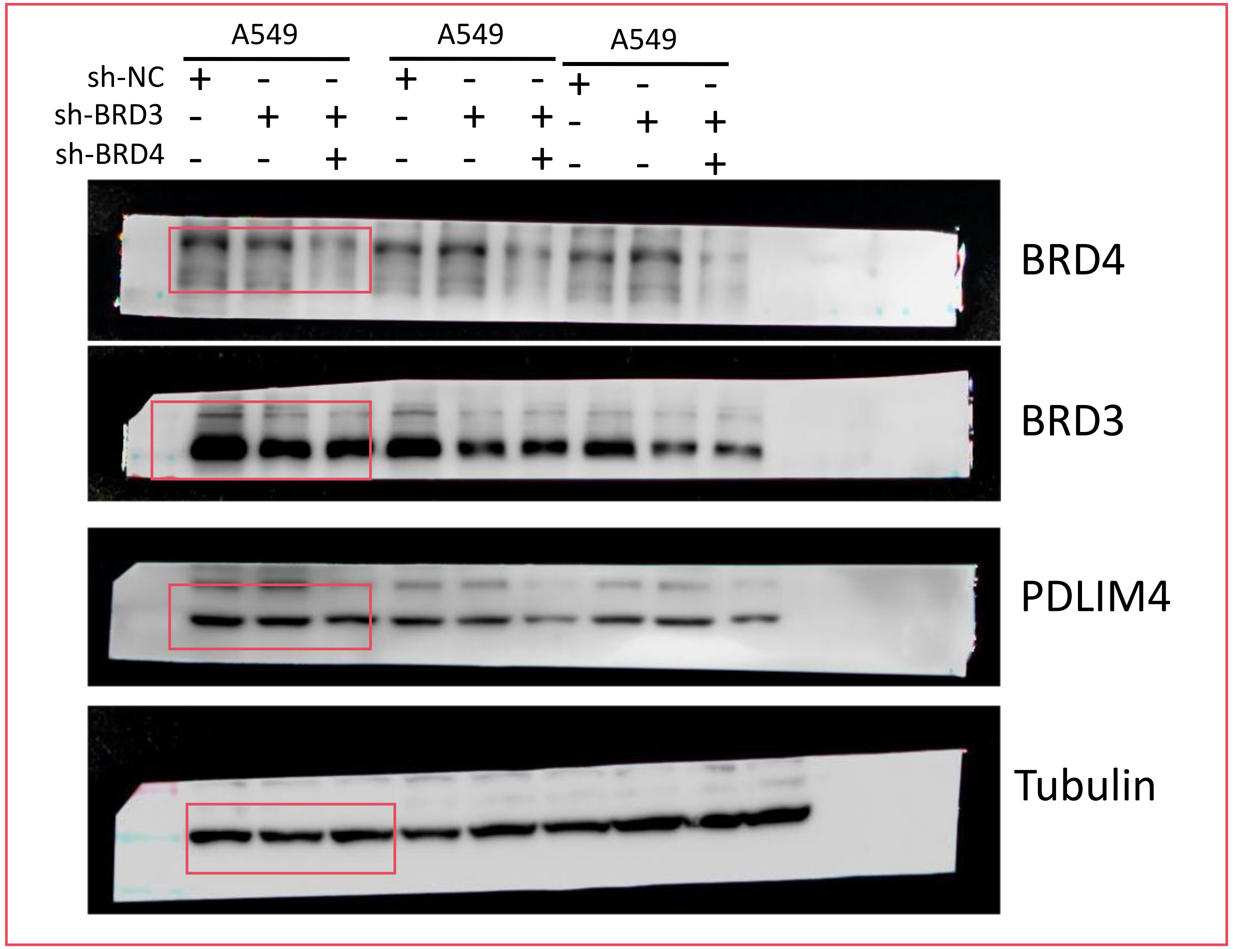

A549 A549 A549
sh-NC + - - + - - + - -
sh-BRD3 - + + - + + - + +
sh-BRD4 - - + - - + - - +
BRD4
BRD3
PDLIM4
Tubulin

W

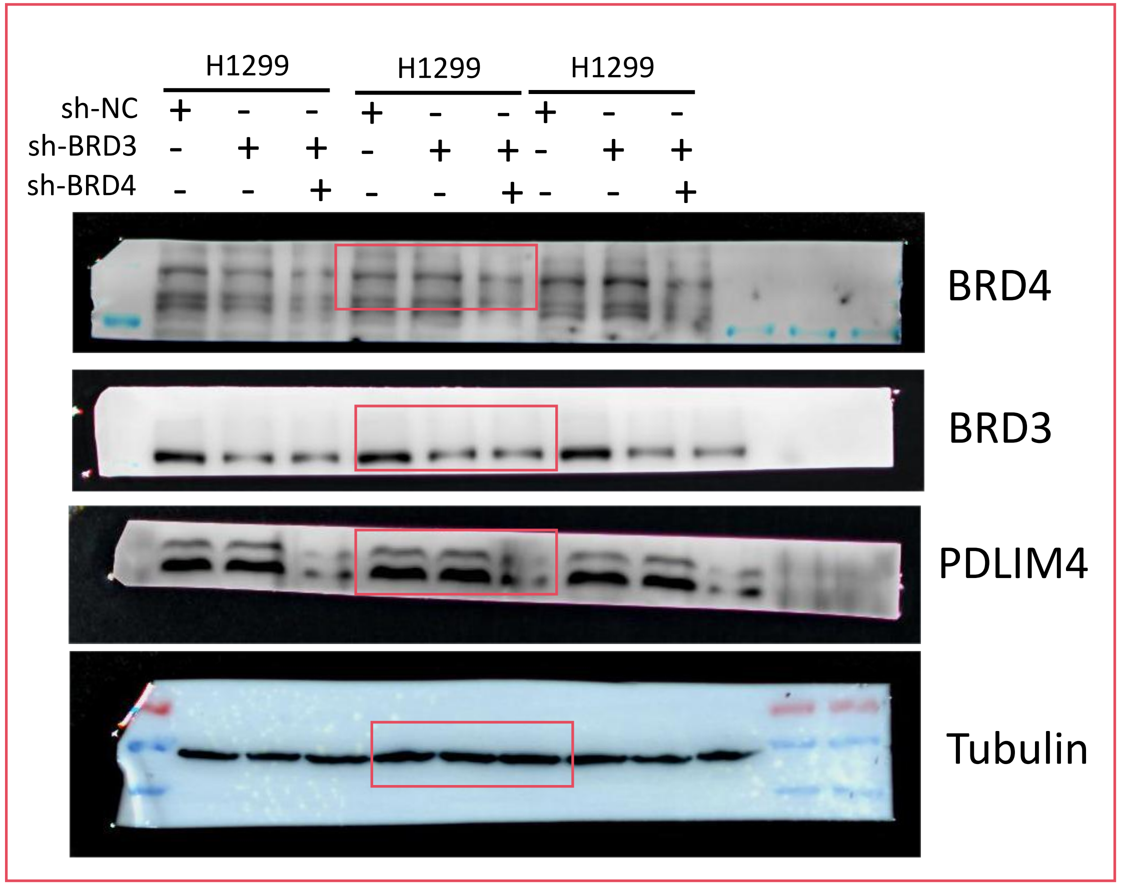

H1299 H1299 H1299
sh-NC + - - + - - + - -
sh-BRD3 - + + - + + - + +
sh-BRD4 - - + - - + - - +
BRD4
BRD3
PDLIM4
Tubulin

Y

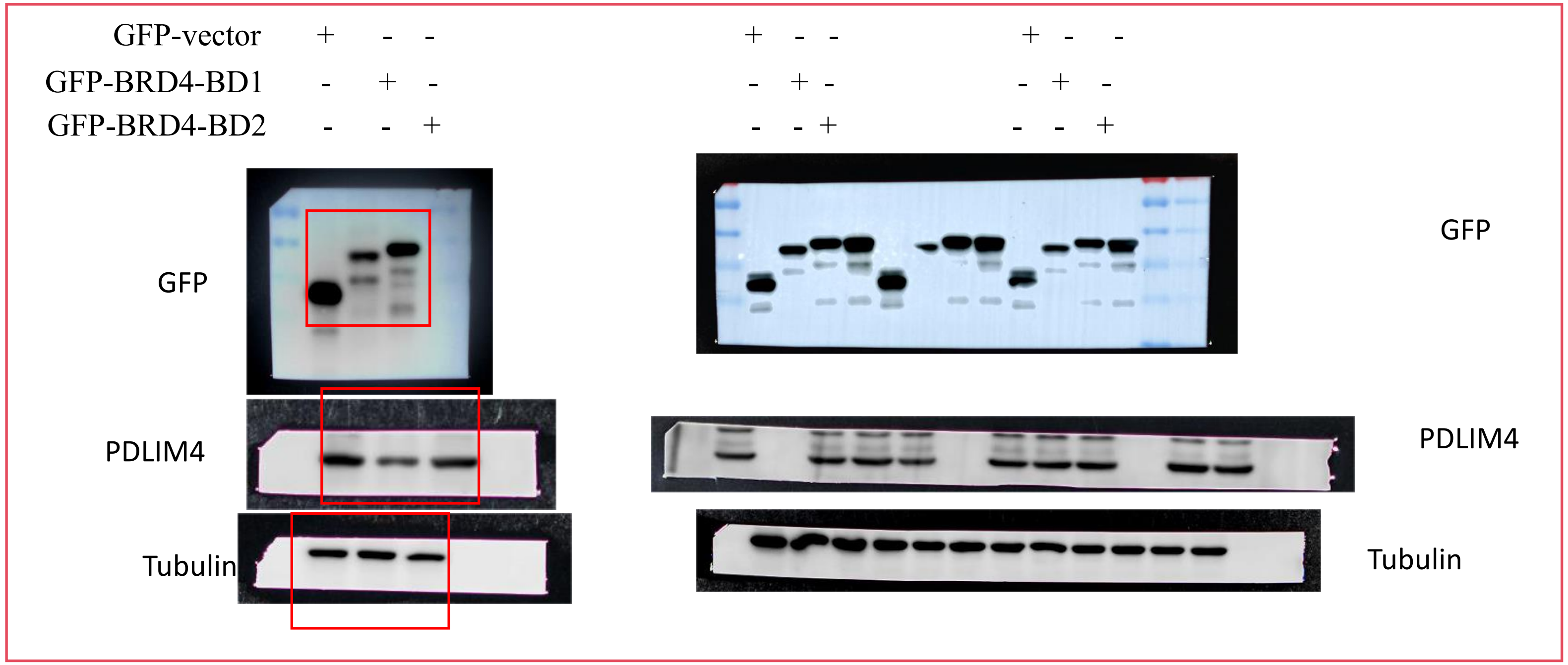
GFP-vector + - - + - - + - -
GFP-BRD4-BD1 - + - - + - - + -
GFP-BRD4-BD2 - - + - - + - - +
GFP
PDLIM4
Tubulin
GFP
PDLIM4
Tubulin

Supplemental data

figure 5

A

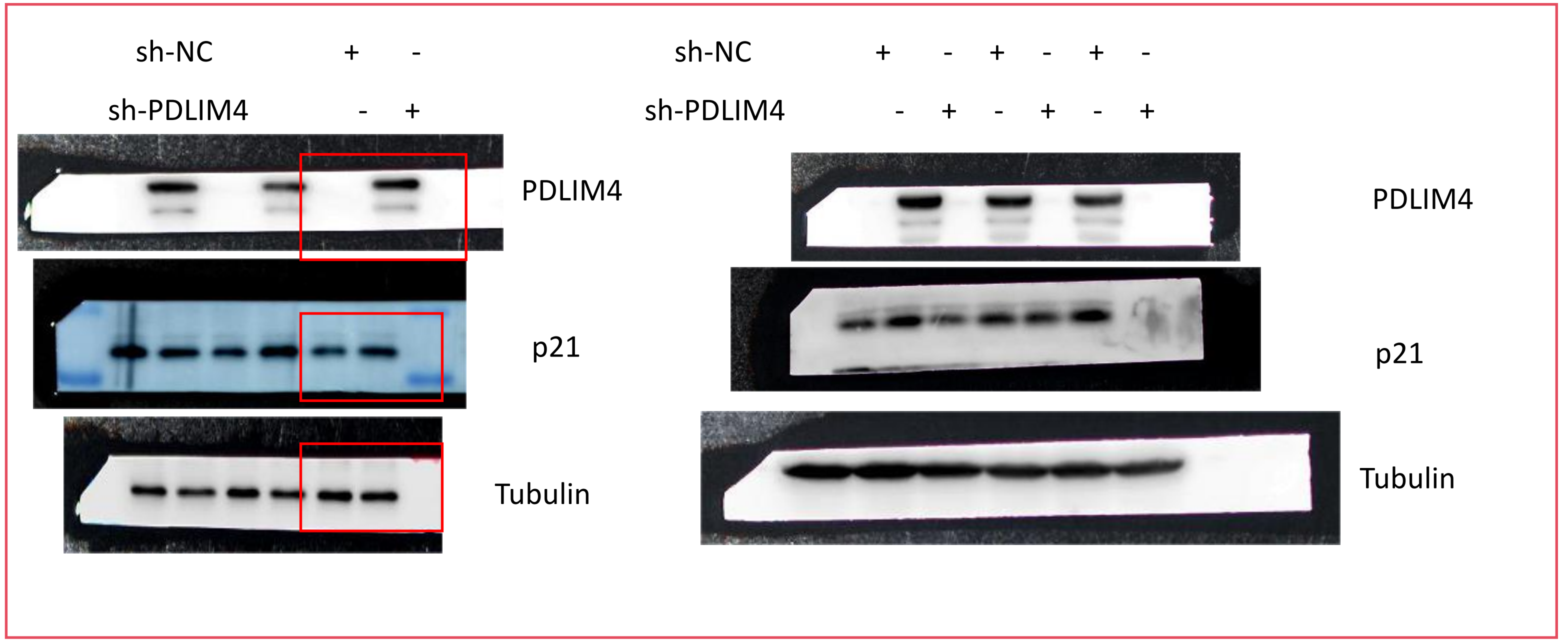

E

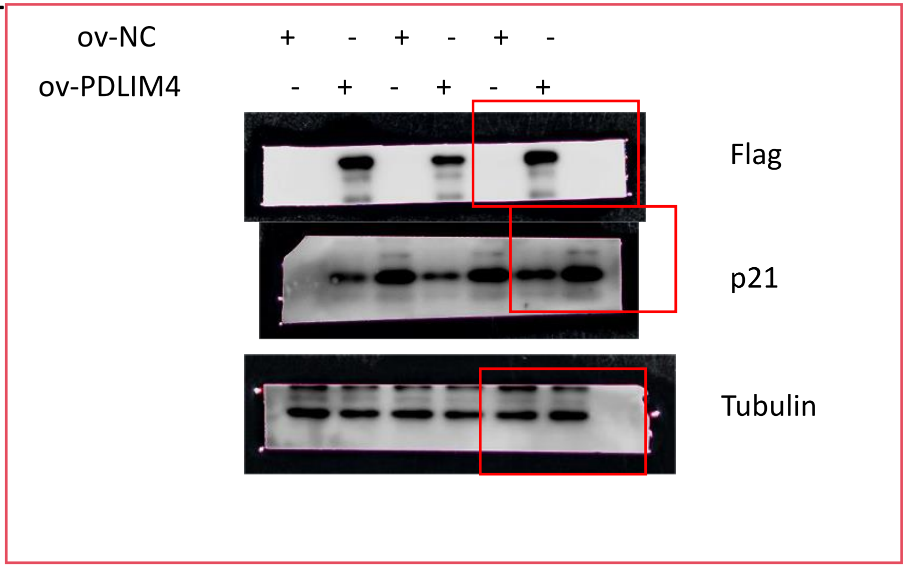

H
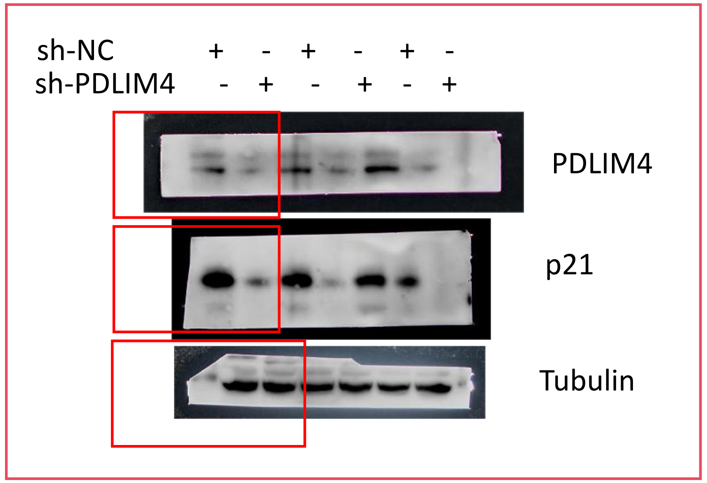
sh-NC + - + - + -
sh-PDLIM4 - + - + - +
PDLIM4
p21
Tubulin

J
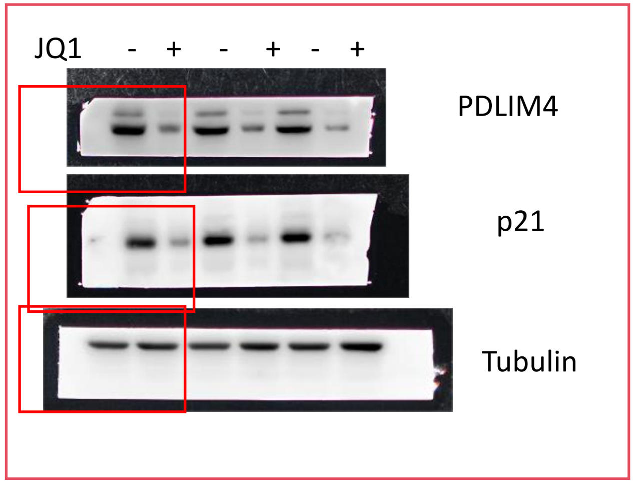
JQ1 - + - + - +
PDLIM4
p21
Tubulin

L
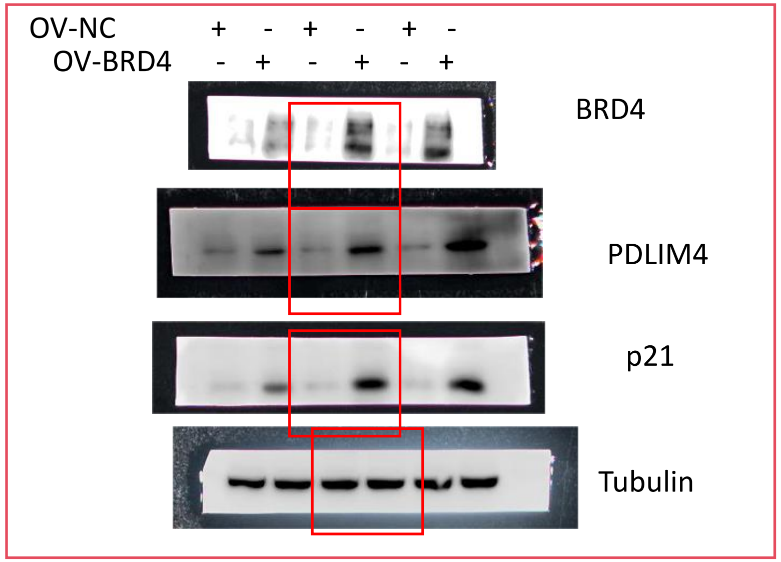
OV-NC + - + - + -
OV-BRD4 - + - + - +
BRD4
PDLIM4
p21
Tubulin

N
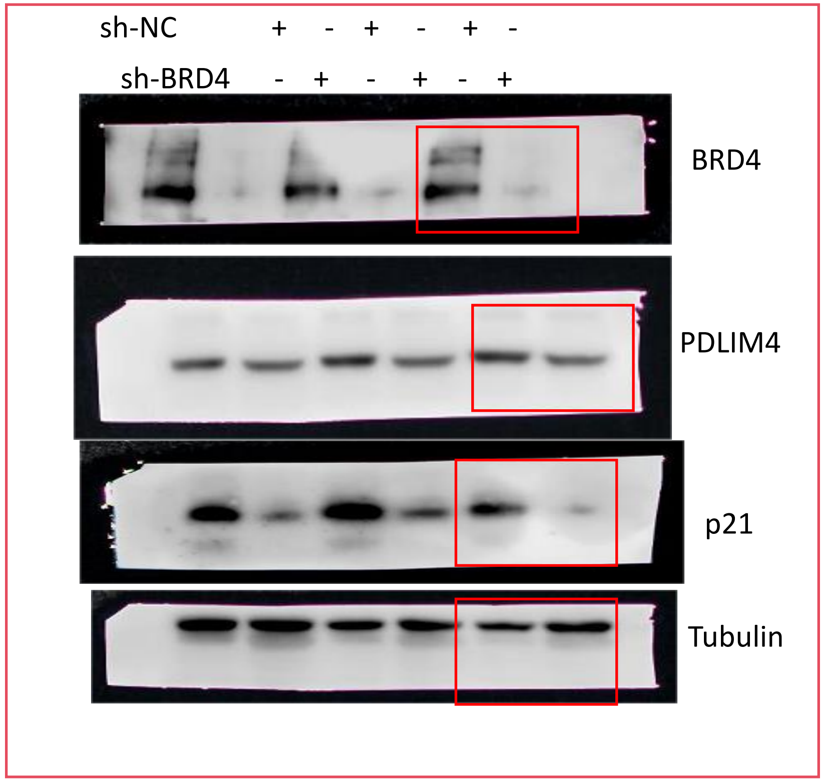
sh-NC + - + - + -
sh-BRD4 - + - + - +
BRD4
PDLIM4
p21
Tubulin

Q
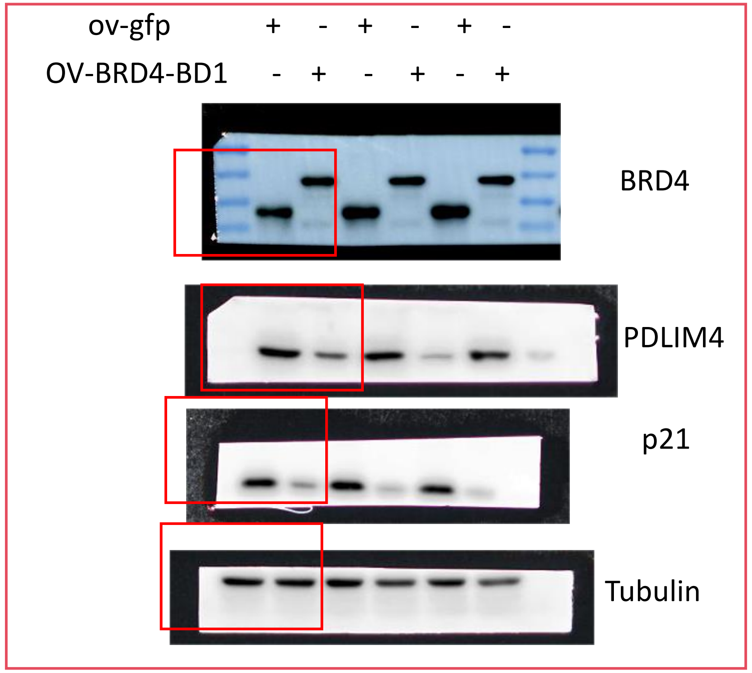
ov-gfp + - + - + -
OV-BRD4-BD1 - + - + - +
BRD4
PDLIM4
p21
Tubulin

T

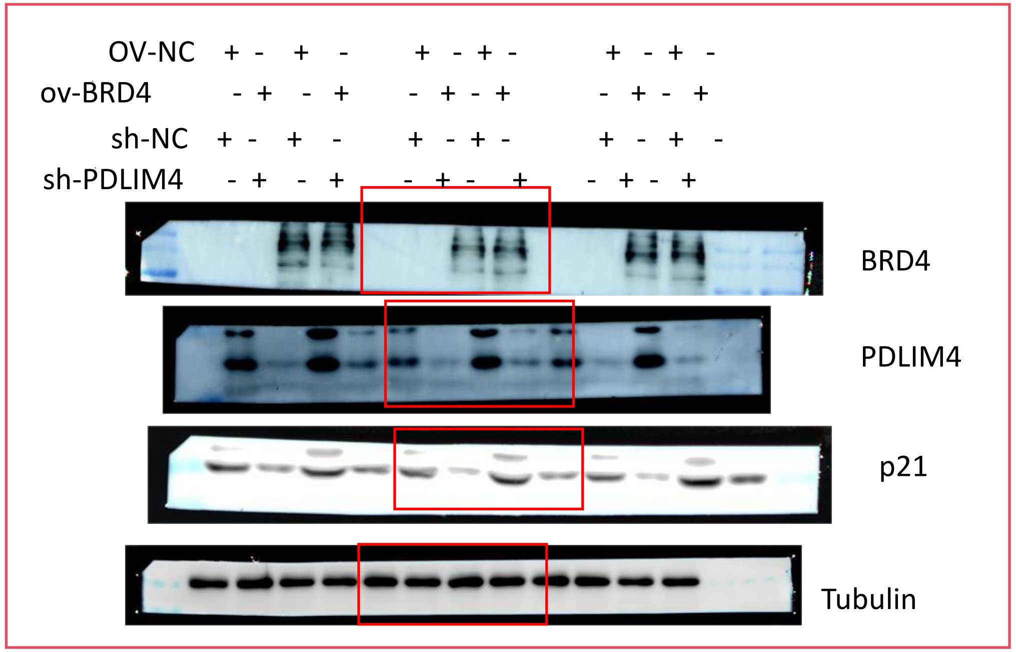
OV-NC + - + - + - + - + - + -
ov-BRD4 - + - + - + - + - + - +
sh-NC + - + - + - + - + - + -
sh-PDLIM4 - + - + - + - + - + - +
BRD4
PDLIM4
p21
Tubulin

V

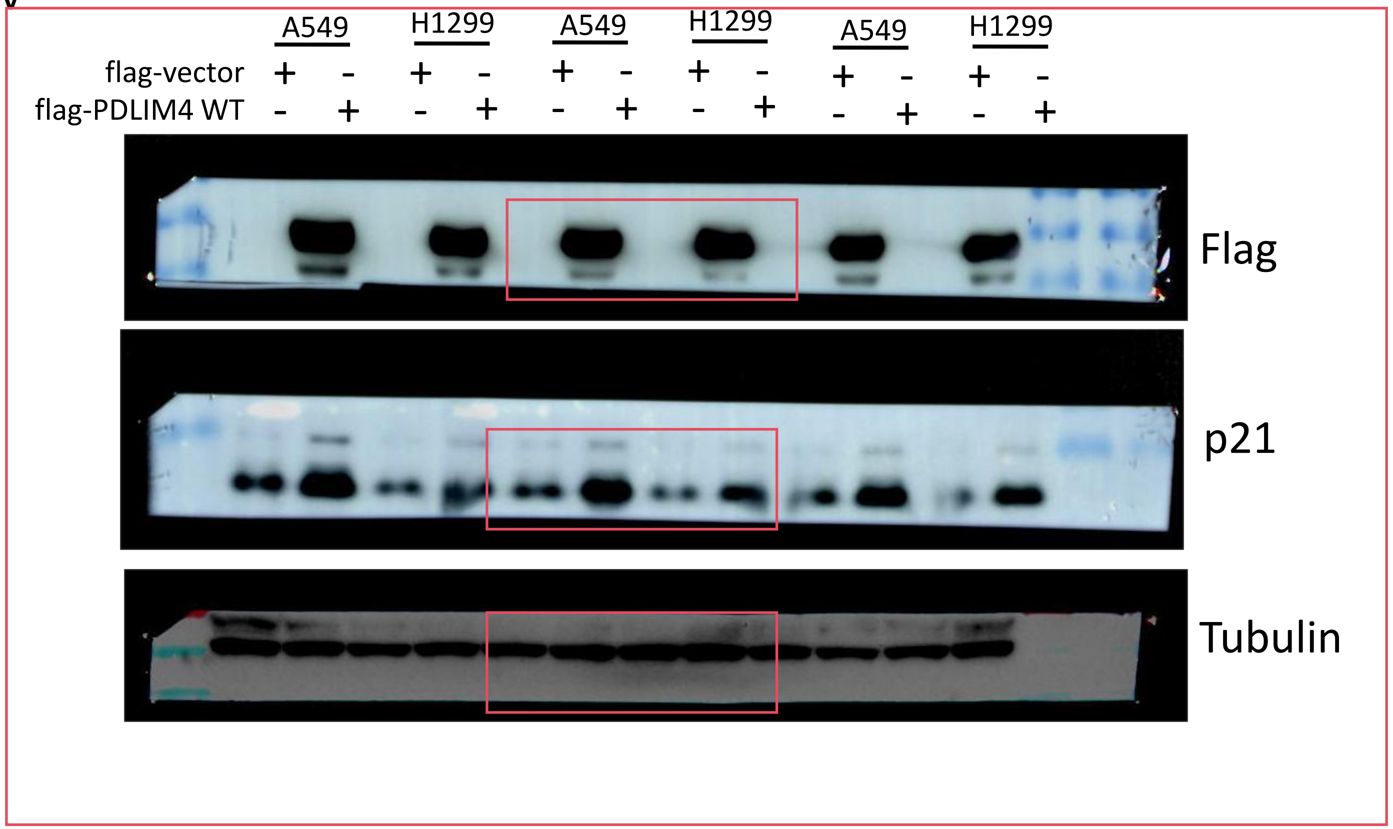
A549
H1299
A549
H1299
A549
H1299
flag-vector + - + - + - + - + - + -
flag-PDLIM4 WT - + - + - + - + - + - +
Flag
p21
Tubulin

X

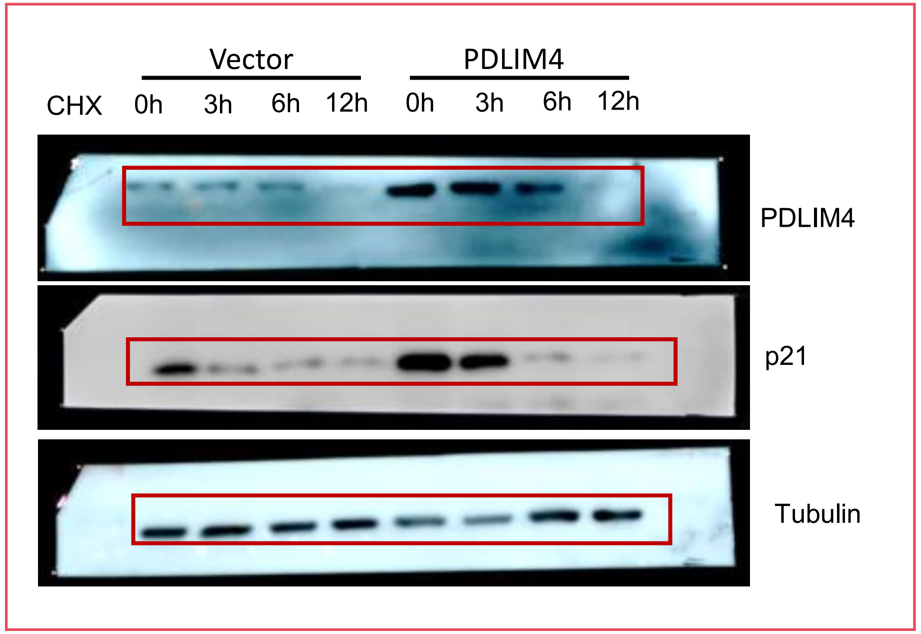
Vector
PDLIM4
CHX 0h 3h 6h 12h 0h 3h 6h 12h
PDLIM4
p21
Tubulin

Supplemental data

figure 6

A

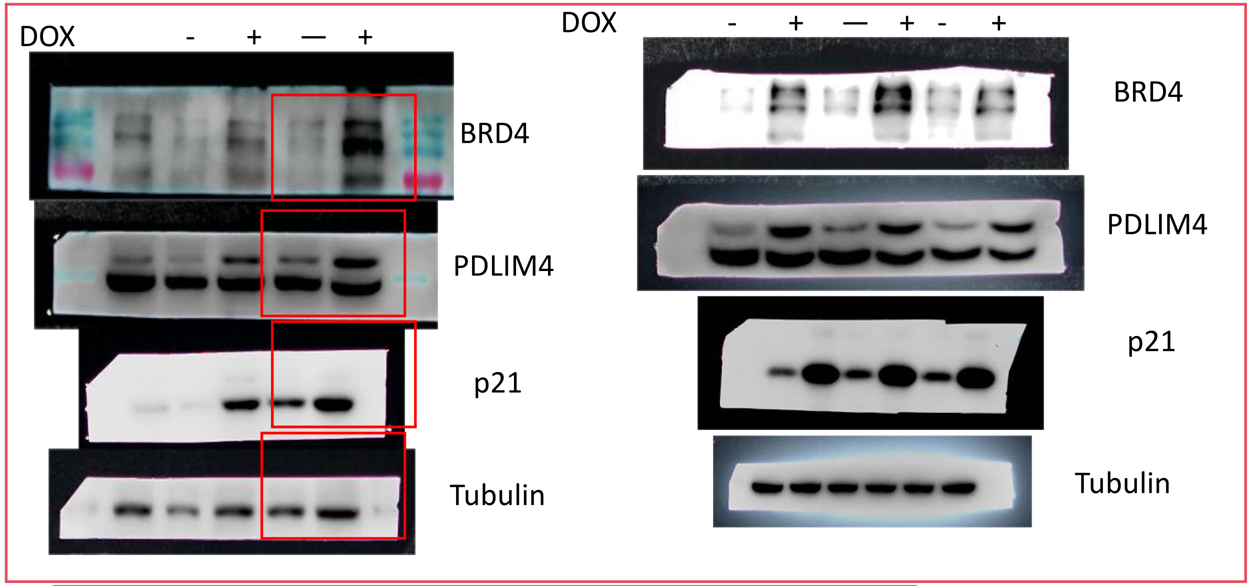

C

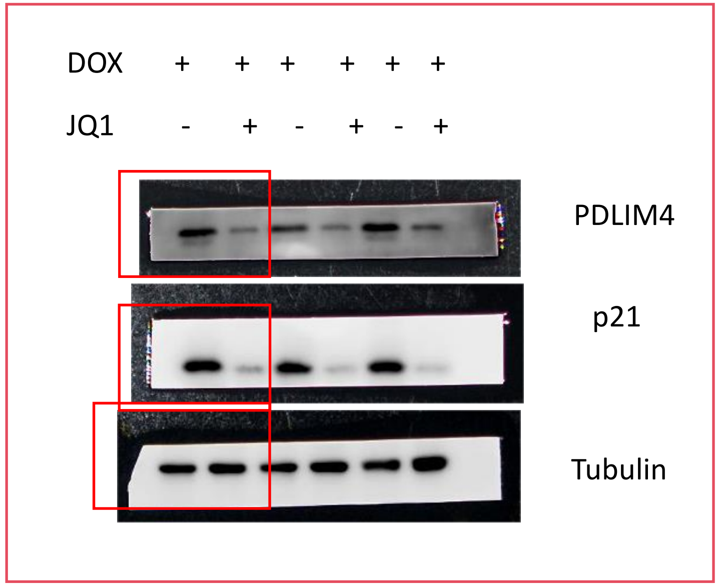

E

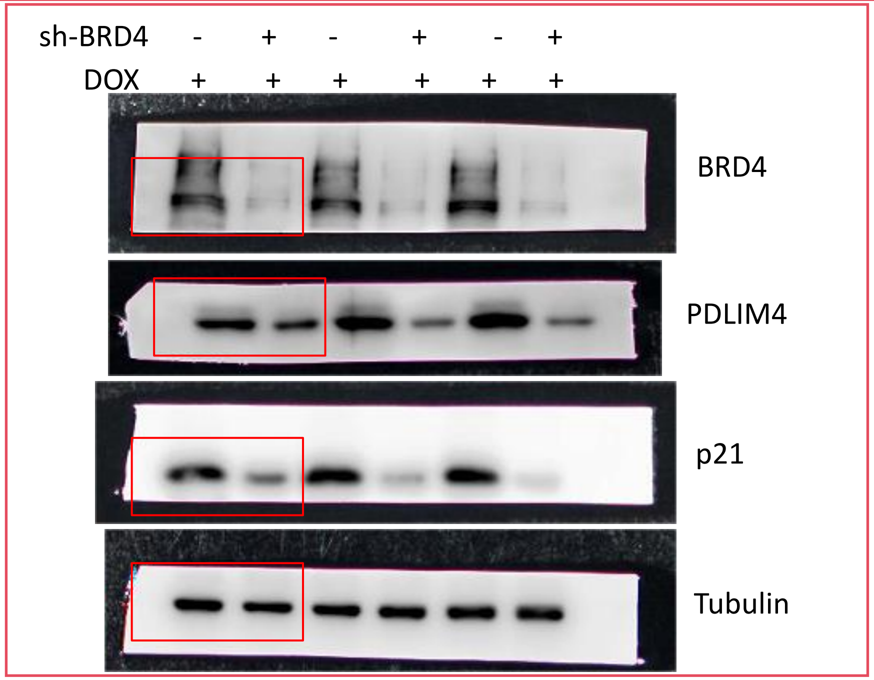

G

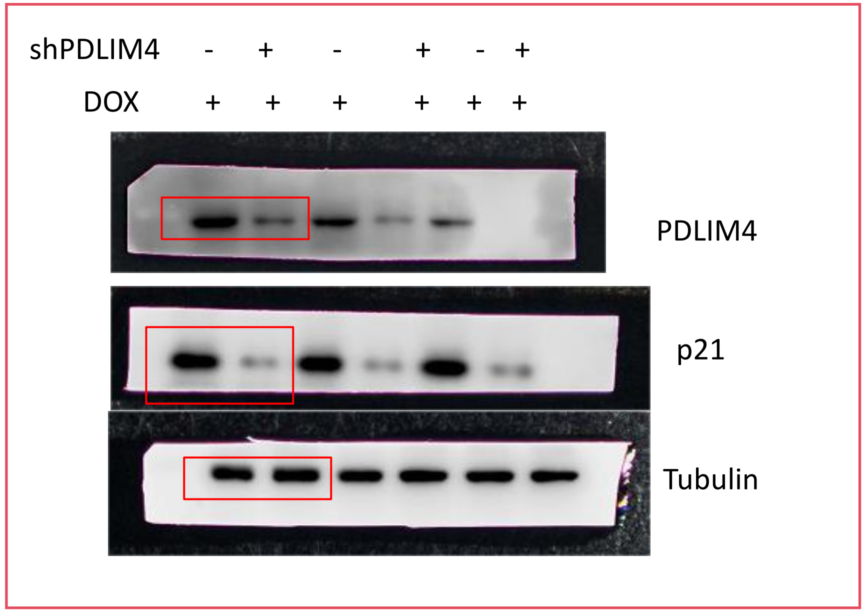

L

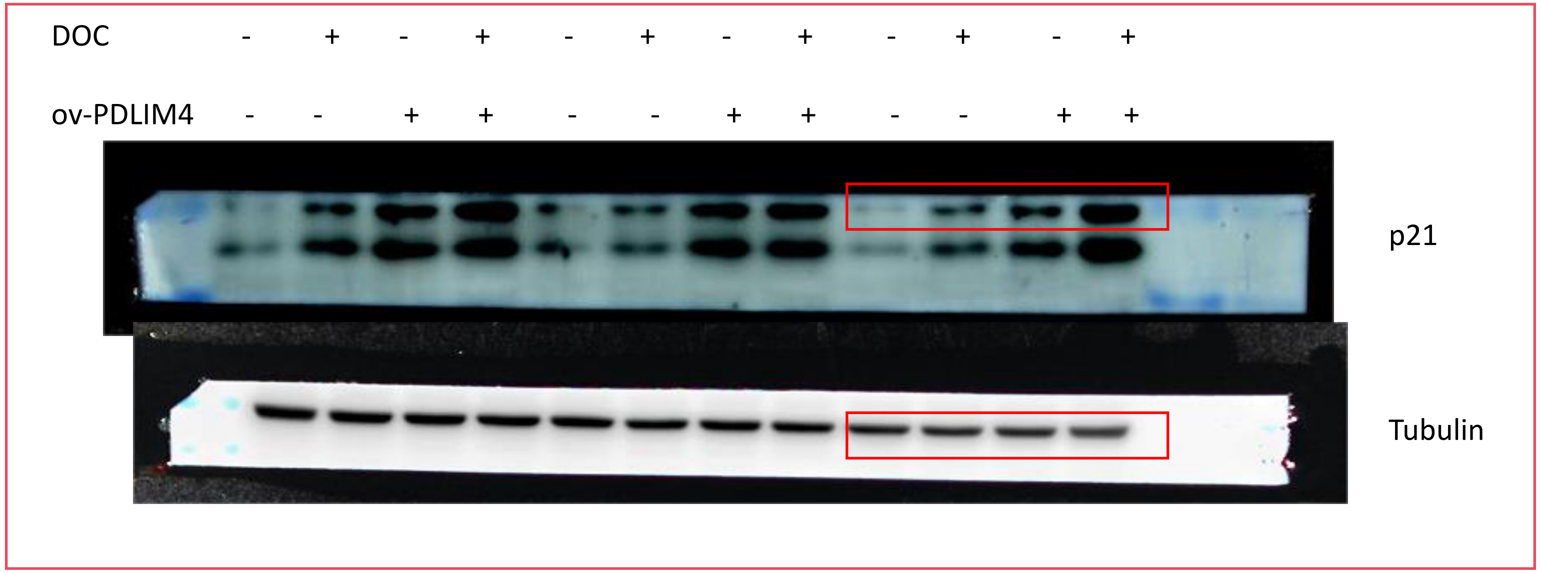

Supplemental data

figure 7

A

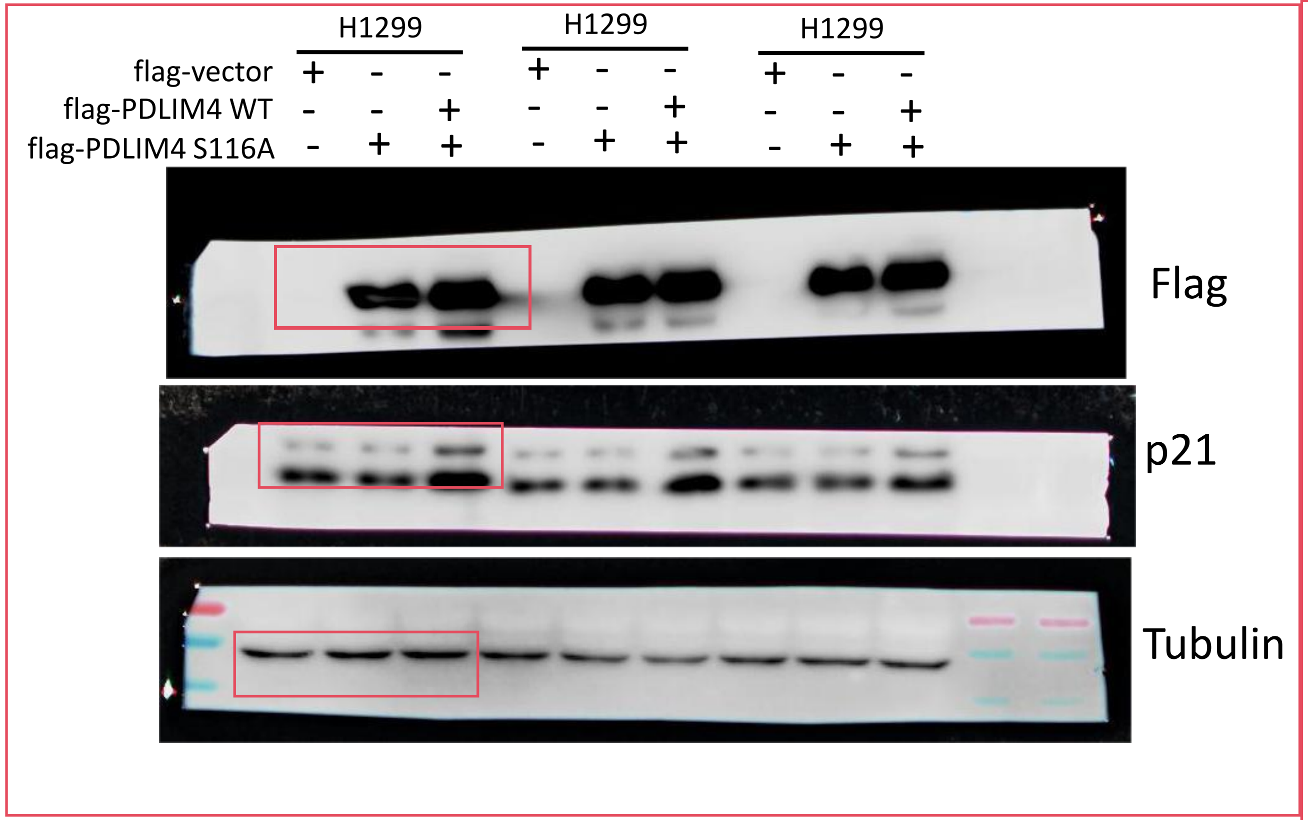

B

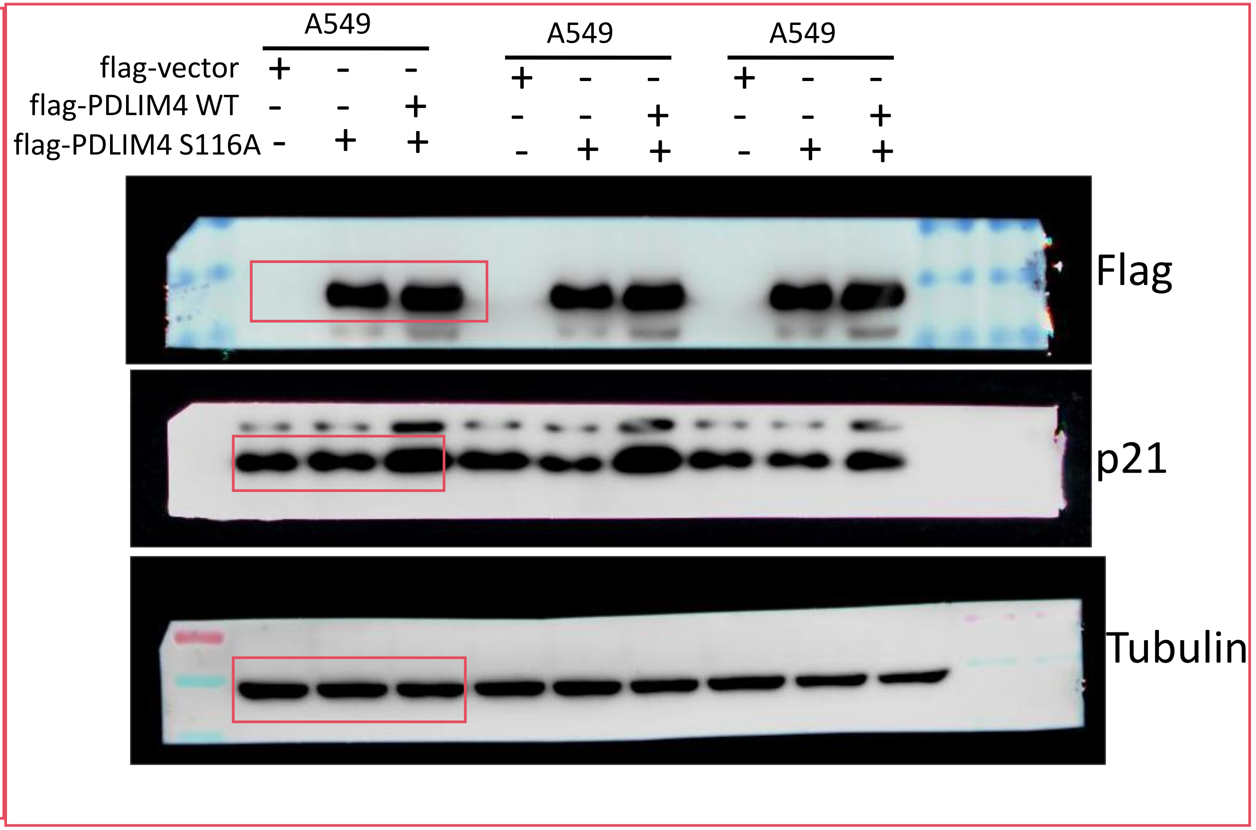

Supplement: Supplementary file 1 — Additional file 1: Figure S1. Stable overexpression or knockdown PDLIM4 cells were constructed. Figure S2. PDLIM4 inhibits proliferation and arrests cell cycle in LUAD cells. Figure S3. BRD4 controls the expression of PDLIM4. Figure S4. PDLIM4 co-localizes with p21 at various period during cell cycle. Figure S5. The regulation of the BRD4/PDLIM4/p21 axis in LUAD cells. Figure S6. BRD4-PDLIM4-p21 pathway sensitizes cells to chemotherapy. Figure S7. Rescue experiment. Table S1. All shRNA and primer sequences used in this paper. Table S2. Sequences of homo primers used for RT-PCR. Original Data. [file 12915_2026_2511_MOESM1_ESM.pdf]
